# Reactivity of Acetylbarbituric Thiosemicarbazone Derivatives with Silver(I) Nitrate and the Influence of Substituents at Nitrogen Atom 4N on the Bonding and Nuclearity of the Resulting Silver(I) Complexes

**DOI:** 10.1021/acsomega.5c05539

**Published:** 2025-09-12

**Authors:** Alfonso Castiñeiras, Nuria Fernández-Hermida, Antonio Frontera, Isabel García-Santos, Lourdes Gómez-Rodríguez

**Affiliations:** † Department of Inorganic Chemistry, Faculty of Pharmacy, University of Santiago de Compostela, Santiago de Compostela 15782, Spain; ‡ Department de Química, 16745 Universitat de les Illes Balears, Crta. de Valldemossa km 7.5, Palma de Mallorca 07122, Spain

## Abstract

The reactions of silver­(I) nitrate with N-substituted thiosemicarbazones derived from 5-acetylbarbituric acid (H_2_Acb4R, *R* = NH_2_, NHCH_3_, N­(CH_3_)_2_, piperidine (Pip), or hexamethylenimine (hexim) in the presence of triphenylphosphine and triethylamine form a series of silver­(I) complexes with various structural motifs and different coordination geometries at the metal, with the composition [Ag­(PPh_3_)_2_(HAcb4NH_2_)] (**1**), [Ag­(PPh_3_)_3_(HAcb4NH_2_)]·EtOH·H_2_O (**2**), Ag­(PPh_3_)­(H_2_Acb4NHM)­(NO_3_)·2H_2_O (**3**), [Ag­(PPh_3_)_3_(HAcb4NHM)]·2EtOH (**4**), Ag­(PPh_3_)­(H_2_Acb4NHM)_2_(NO_3_)·3H_2_O (**5**), [Ag­(PPh_3_)_3_(HAcb4NDM)]·3EtOH (**6**), [Ag­(PPh_3_)_3_(HAcb4NDM)]·2DMSO·H_2_O (**7**), [Ag­(PPh_3_)­(HAcb4Pip)]_3_·4.5H_2_O (**8**) and [Ag­(PPh_3_)_3_(HAcb4Hexim)]·3EtOH·3H_2_O (**9**), and {[Ag­(H_2_O)_14_]­(NO_3_)}­n, [Ag­(PPh_3_)_4_]­(NO_3_)·EtOH, [AgCl­(PPh_3_)]_4_ as byproducts. In many cases, polycrystalline samples of these compounds were characterized using elemental analysis, FT-IR, UV–Visible, ^1^H, ^13^C­{^1^H} and ^31^P­{^1^H} NMR spectroscopy, FAB-MS, and single-crystal X-ray crystallography. The spectroscopic properties and structures of these compounds, except **1**, **3**, and **5**, are discussed. Various hydrogen-bonding interactions, such as N–H···O, O–H···O, C–H···O, and π–π stacking interactions, contribute to the crystal packing. The influence of these weak interactions has been addressed with the help of Hirshfeld surface analyses and further analyzed using DFT calculations and Bader’s theory of atoms-in-molecules.

## Graphical abstract

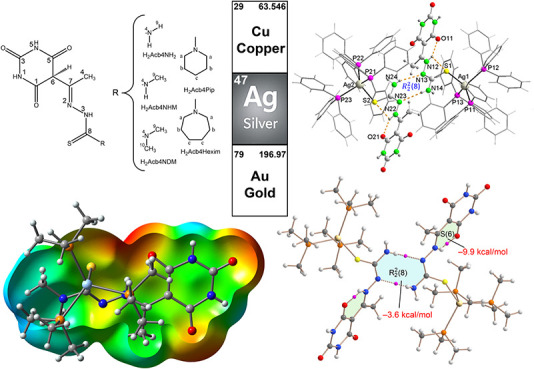

## 1 Introduction

In recent years, complexes formed by transition metal ions coordinated by ligands containing the thioamide group have attracted research interest because many exhibit pharmacological activity, including diverse biological properties such as antitumor, antifungal, antibacterial, antiviral and anticancer properties. This is particularly evident when aldehyde derivatives containing heterocycles are combined with thiosemicarbazides, resulting in the synthesis of novel thioamides that exhibit enhanced pharmacological activity.^1^ Among the compounds under consideration, thiosemicarbazones (TSCs) are particularly noteworthy due to their structural diversity chemical properties.^2,3^

The remarkable versatility of thiosemicarbazones has enabled the exploration of a diverse array of biological activities. This is achieved by introducing structural changes through novel chemical substitutions and coordinating with transition metal ions. This offers a potential avenue for synthesizing new compounds with pharmacological activity. These ligands facilitate the formation of complexes that exhibit enhanced bioactivities, or, in the case of the free ligand, endow it with specific characteristics due to their varied coordination modes with transition metal ions. As it is well-known, these compounds have a structure of N-NH-C­(S)-NR_2_, which allows them to behave in coordination chemistry as monodentate, ambidentate or multifunctional donor ligands, through their thiocarbonyl sulfur and azomethine nitrogen atoms, which can bind to a metal center in different coordination modes, presenting thione-thiol tautomerism; therefore, they act as neutral thione and monodeprotonated thiolate ligands, to form mononuclear, dinuclear or polynuclear compounds with different structural features, such as S or N monodentate, μ_2_-S, N-bridges, chelate-N,S, μ_2_-N,S-bridge, μ_2_-N,S-(η^2^-S) bridge, or μ_3_-N,S-(η^2^-S, η^1^-N) bridge, among others.^4^

In the field of silver­(I) coordination chemistry, the metal has the capacity to adopt a variety of coordination numbers, including two, three, four, and six. This phenomenon leads to the formation of different structural types. According to Pearson’s terminology, the soft Ag^+^ ion exhibits a natural predilection for soft bases, particularly ligands containing sulfur as the primary donor atom. In sum, silver­(I) complexes with this type of ligand have a wide range of applications in medicine, analytical chemistry, and the polymer industry. A significant proportion of the biomedical applications and uses of silver­(I) complexes are associated with their antibacterial properties, which appear to involve interaction with DNA.^5^ From an application perspective, silver currently occupies a position of paramount importance among metals. Prior to the advent of antibiotics, silver­(I) salts were employed as antiseptic, antibacterial, and anti-inflammatory. However, contemporary research in the field of silver­(I) has been predominantly focused on the development of coordination compounds, clusters, and nanomaterials,^6^ these substances possess noteworthy medicinal and industrial values as antimicrobial^7^ and anti-inflammatory^8^ or anticancer agents.^9^ Moreover, specific silver­(I) complexes have demonstrated remarkable efficacy in the context of homogeneous catalysis, facilitating the preparation of novel compounds that facilitate the activation of C–C and C–N bonds.^10,11^ Due to their high biocidal activity, silver complexes are suggested as agents with multiple applications for agricultural purposes as well as other sectors.^12^

The atomically precise control of the geometry and size of silver­(I) complexes based on the Ag–S bond is especially attractive because of the importance of structure–property relationships in constructing new materials for diverse applications such as catalysis,^13^ sensors,^14^ and luminescence.^15^

Another interesting aspect of silver­(I) coordination chemistry concerns complexes with phosphines. The incorporation of these organophosphorus substances as sigma donors and pi acceptors allows the formation of stable complexes with many metal ions, including silver­(I).^16^ Coordination compounds of silver­(I) salts with tertiary phosphines present a diversity of structural types, which has led to an expansion in the investigation of these complexes in addition to their potential application as active pharmaceutical ingredients (APIs), including anticancer agents for the diagnosis and prevention of cancer and its treatment, based on the analogy of Ag­(I) salts with those of Au­(I). However, until recently, this type of study has been limited to certain analogs of cationic lipophilic complexes,^17,18^ which generally consist of the metal ion chelated between bidentate phosphines creating a tetrahedral environment. They have found some application in homogeneous catalysis and also as antitumor compounds. While little has been published on the interaction of diphosphines with silver and their applications, even less has been published on the use of monophosphines, so work in this field is of interest.

The assembly, geometry, and size of the resulting silver complexes are often influenced by auxiliary ligands. There are some articles describing metal complexes combining thioamide and triphenylphosphine ligands;^19,20^ however, their number is negligible compared to metal complexes containing only thioamides and much lower when considering those containing thiosemicarbazones.

In light of the above, it is evident that there is a clear need in the art to explore the potential of mixed complexes of thiosemicarbazones and silver­(l) monophosphines, in order to understand, first, the factors governing their structural aspects and, second, to study the molecular mechanisms through which these complexes achieve potential applications.

Based on this background, we decided to delve deeper into the relationship between the molecular structures of heterocyclic silver­(I) complexes with mixed thioamide (thiosemicarbazone)/triphenylphosphine ligands. In this work we report the synthesis of nine new silver­(I) complexes, and crystalline byproducts, featuring specific combinations of N-substituted thiosemicarbazones derived from 5-acetylbarbituric acid (or thioamidates), shown in Chart [Fig chart1], and a variable number of PPh_3_ ligands in their coordination sphere, in the presence (or absence) of a nitrate ion. The structural effects of the nature of the thioamide ligands and the different coordination environments of the complexes are also studied. The R groups of these thiosemicarbazones were selected for a number of reasons, primarily, to allow for comparison of the structures of the compounds. Additionally, the R groups were chosen due to their potential involvement in intramolecular or intermolecular interactions. Finally, the R groups were considered because of their probable influence on the hydrophilicity and lipophilicity of the prepared complexes, which is great importance for their potential pharmaceutical activity.

**1 chart1:**
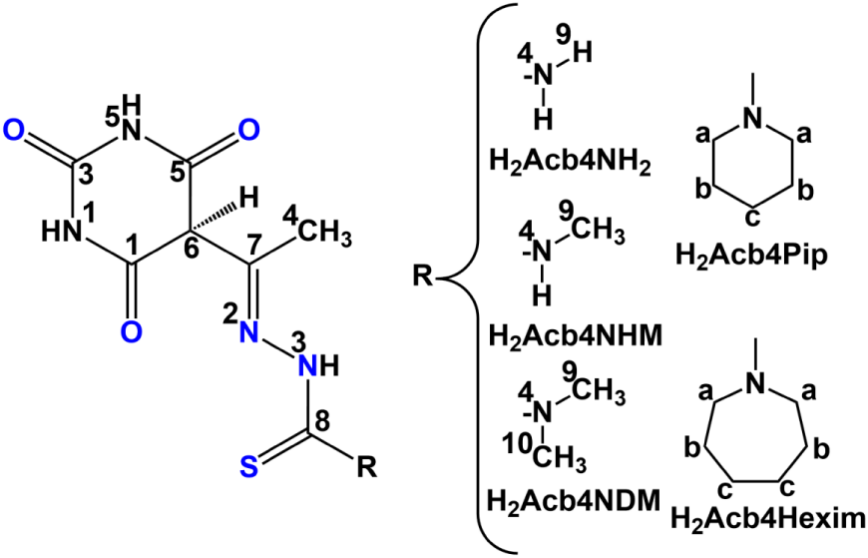
Used thiosemicarbazones derived from 5-acetylbarbituric acid, with atom-labeling for NMR analysis

## 2 Experimental Section

### 2.1 Physical Measurements

All reagents and solvents were commercial products that were used as received, without further purification, but the reactions involving silver salts were carried out at room temperature and in the absence of light. Melting points were determined on a Büchi melting point apparatus and are uncorrected. Microanalyses (C, H, N, and S) were carried out using a Carlo-Erba 1108 elemental analyzer. ^1^H, ^13^C­{^1^H} and ^31^P­{^1^H} NMR spectra were obtained as DMSO-*d*_6_ and MeOH-*d*_4_ solutions with a Bruker AMX 300 spectrometer. IR spectra were recorded as KBr disks (4000–400 cm^–1^) with a Bruker IFS-66v spectrometer. Mass spectra were obtained using a Micromass AUTOSPEC spectrometer (nitrobenzyl alcohol matrix) for FAB^+^. The UV–vis spectra were recorded in the MeOH solvent with a 10^–4^ M concentration using UV–vis Kontron Instruments Uvikon 810P Spectrophotometer with 1 cm quartz cell, in the range 250–600 nm.

### 2.2 Synthesis and Crystallization of 5-Acetylbarbituric Hydrazine-1-carbothioamides (H_2_Acb4R, Chart I)

The thiosemicarbazones were prepared according to literature procedures by condensation reactions between 5-acetylbarbituric acid and the *N* ^4^-unsubstituted/substituted thiosemicarbazides.^21^

### 2.3 Synthesis and Crystallization of Complexes

In the general procedure for the preparation of the complexes, a few drops of triethylamine were added to a solution obtained by adding 0.12 mmol of the corresponding thiosemicarbazone in 15 mL of absolute ethanol, which resulted in a yellow solution. Subsequently, 0.12 mmol of AgNO_3_ was added dropwise which stirring. This addition was maintained for 24 h. Thereafter, a solution of 0.12 mmol of triphenylphosphine in 5–10 mL of absolute ethanol was added, and the mixture was stirred for at least 6 days. Finally, the resulting suspension was filtered or subjected to centrifugation, and the solid residue was washed with small portions of absolute ethanol.

#### 2.3.1 Reactions with 5-Acetylbarbituric Hydrazine-1-carbothioamide (H_2_Acb4NH_2_)

After a period of 1 month, single crystals of **{[Ag­(H**_**2**_ **O)**_**14**_ **]­(NO**_**3**_)}_**n**_, suitable for X-ray diffraction studies, emerged in the mother liquor and were removed manually. Additionally, a powdered solid, showing a whitish appearance, was filtered off and identified through elemental analysis as [Ag­(PPh_3_)_2_(HAcb4NH_2_)] (**1**).

##### 2.3.1.1 Data for [Ag­(PPh_3_)_2_(HAcb4NH_2_)] (**1**):

Yield: 0.028g (27%), mp 220 °C. Elemental analysis found: C, 59.3; H, 4.2; N, 7.8; S, 3.4. Calc. for C_43_H_38_AgN_5_O_3_P_2_S (872.67): C, 59.0; H, 4.4; N, 8.0; S, 3.7%. IR (ν_max_/cm^–1^): 3431 ν­(OH); 3053 ν­(NH); 1383, 1710, 1614 ν­(C=O)+δ­(NH); 1479 ν­(CN+CC); 1477–997 ν­(phenyl); 1309 ν­(CS+CC); 1069 ν­(NN); 1094 ν_s_(P-CPh); 783 ν­(CS); 524, 492 ν_as_(P–C_Ph_); 438, 429 ν­(Ag–P); and 253 ν­(Ag–S).

The slow evaporation of the mother liquor at room temperature, which resulted from filtration of a white solid, led to the formation of two types of single crystals that were suitable for X-ray analysis. These were identified as [Ag­(PPh_3_)_4_]­(NO_3_)·EtOH and [Ag­(PPh_3_)_3_(HAcb4NH_2_)]·EtOH·H_2_O (**2**).

##### 2.3.1.2 Data for [Ag­(PPh_3_)_4_]­(NO_3_)·EtOH:

Elemental analysis found: C, 69.3; H, 5.2; N, 0.8. Calc. for C_74_H_66_AgNO_4_P_4_ (1265.08): C, 70.5; H, 5.3; N, 1.1%. IR (ν_max_/cm^–1^): 3441 ν­(OH); 3053–2975 ν­(CH); 1477–997 ν­(phenyl); 1400 ν­(NO_3_); 1092 ν_s_(P–C_Ph_); 541, 512, 492 ν_as_(P–C_Ph_); 437, 398 ν­(Ag–P). FAB^+^ MS [*m*/*z*, assignment]: 895 [Ag­(PPh_3_)_3_]^+^; 631(42) [Ag­(PPh_3_)_2_]^+^; 369(46) [Ag­(PPh_3_)]^+ 1^H NMR (DMSO-*d*_6_, ppm): 7.11–7.59 (PPh_3_) ^13^C­{^1^H} NMR (DMSO-*d*_6_, ppm): 133.9, 132.6, 130.5, 129.4 (PPh_3_). ^31^P­{^1^H} NMR (DMSO-*d*_6_, 25 °C, ppm): 45.6; 29.6; 5.8. (DMSO-*d*_6_, −60 °C, ppm): 44.6, 35.4, 8.1–6.9.

##### 2.3.1.3 Data for [Ag­(PPh_3_)_3_(HAcb4NH_2_)]·EtOH·H_2_O (**2**):

Elemental analysis found: C, 63.3; H, 5.2; N, 5.8; S, 3.4. Calc. for C_63_H_61_AgN_5_O_5_P_3_S (1201.04): C, 63.0; H, 5.1; N, 5.8; S, 3.7%. IR (ν_max_/cm^–1^): 3436 ν­(OH); 3175, 3052 ν­(NH); 1383, 1707, 1619 ν­(C=O)+δ­(NH); 1479 ν­(CN+CC); 1454–997 ν­(phenyl); 1355, 1309 ν­(CS+CC); 1070 ν­(NN); 1093 ν_s_(P–C_Ph_); 784 ν­(CS); 513ν_as_(P–C_Ph_).

#### 2.3.2 Reactions with *N*-Methyl-(5-acetylbarbituric)­hydrazine-1-carbothioamide (H_2_Acb4NHM)

The brown powdery solid was identified by elemental analysis as Ag­(PPh_3_)­(H_2_Acb4NHM)­(NO_3_)·2H_2_O (**3**) and the slow concentration of the mother liquor at room temperature afforded colorless plate crystals suitable for a single-crystal X-ray analysis of [Ag­(PPh_3_)_3_(HAcb4NHM)]·2EtOH (**4**). The same synthesis was carried out AgNO_3_:H_2_Acb4NHM:PPh_3_ with a 1/2/1 molar ratio instead of 1/1/1, obtaining a solid of formula Ag­(PPh_3_)­(H_2_Acb4NHM)_2_(NO_3_)·3H_2_O (**5**), and from mother liquor new crystals of [Ag­(PPh_3_)_3_(HAcb4NHM)]·2EtOH (**4**).

##### 2.3.2.1 Data for Ag­(PPh_3_)­(H_2_Acb4NHM)­(NO_3_)·2H_2_O (**3**):

Yield: 0.014 g (16%), mp >250 °C. Elemental analysis Found: C, 43.4; H, 4.0; N, 12.1; S, 4.4. Calc. for C_26_H_30_AgN_6_O_8_PS (725.46): C, 43.0; H, 4.2; N, 11.6; S, 4.4%. IR (ν_max_/cm^–1^): 3383 ν­(OH); 3312–3003 ν­(NH); 1706, 1619 ν­(C=O)+δ­(NH); 1519, ν­(CN+CC); 1456–998 ν­(phenyl); 1398 ν­(NO), 1354, 1280, 1230 ν­(CS+CC); 1095 ν_s_(P–C_Ph_); 1064 ν­(NN); 783 ν­(CS); 525, 490 ν_as_(P–C_Ph_); 435, 421 ν­(Ag–P); 251 ν­(Ag–S). ^1^H NMR (DMSO-*d*_6_, ppm): 14.47 (1H, N2H); 10.16 (1H, N1H); 10.16 (1H, N5H); 2.57 (3H, CH_3_); 6.59 (1H,N4H); 2.63 (H,N4Me); 7.27–7.50 (PPh_3_). ^31^P­{^1^H} NMR (DMSO-*d*_6_, ppm): 13.12.

##### 2.3.2.2 Data for [Ag (PPh_3_)_3_(HAcb4NHM)]·2EtOH (**4**):

Yield: 0.012 g (8%), mp 150 °C. Elemental analysis Found: C, 63.7; H, 4.9; N, 6.0; S, 2.6. Calc. for C_82_H_56_AgN_5_O_5_P_3_S (1243.12): C, 63.7; H, 5.4; N, 5.6; S, 2.6%. IR (ν_max_/cm^–1^): 3441 ν­(OH); 3330–3003 ν­(NH); 1699, 1629 ν­(C=O)+δ­(NH); 1497 ν­(CN+CC); 1479–998 ν­(phenyl); 1384, 1353, 1265, 1225 ν­(CS+CC); 1069 ν­(NN); 1093 ν_s_(P–C_Ph_); 804 ν­(CS); 512, 489 ν_as_(P–C_Ph_); 428 ν­(Ag–P); 256 ν­(Ag–S). FAB^+^ MS [*m*/*z*, assignment]: 631(32) [Ag (PPh_3_)_2_]^+^; 369(16) [Ag­(PPh_3_)]^+^; 258(2) [HAcb4NHCH_3_]^+^.

##### 2.3.2.3 Data for Ag­(PPh_3_)­(H_2_Acb4NHM)_2_(NO_3_)·3­(H_2_O) (**5**):

Yield: 0.047 g (39%), mp >250 °C. Elemental analysis Found: C, 40.3; H, 4.3; N, 15.6; S, 6.8. Calc. for C_34_H_43_AgN_11_O_12_PS_2_ (1000.75): C, 40.8; H, 4.3; N, 15.4; S, 6.4%. IR (ν_max_/cm^–1^): 3409 ν­(OH); 3195–3052 ν­(NH); 1702, 1621 ν­(C=O)+δ­(NH); 1519 ν­(CN+CC); 1456–1036 ν­(phenyl); 1397 ν­(NO), 1385, 1354, 1277, 1227 ν­(CS+CC); 1072 ν­(NN); 1094 ν_s_(P–C_Ph_); 781 ν­(CS); 527, 498 ν_as_(P–C_Ph_); 428 ν­(Ag–P); 262 ν­(Ag–S). ^1^H NMR (DMSO-*d*_6_, ppm): 14.38 (1H, N2H); 10.11 (1H, N1H); 10.11 (1H, N5H); 2.59 (3H, CH_3_); 6.39 (1H,N4H); 2.64 (H,N4Me); 7.23–7.47 (PPh_3_) ^31^P­{^1^H} NMR (DMSO-*d*_6_, ppm): 13.33.

#### 2.3.3 Reactions with *N*,*N*-Dimethyl-(5-acetylbarbituric)­hydrazine-1-carbothioamide (H_2_Acb4NDM)

A few colorless plate crystals of [Ag­(PPh_3_)_3_(HAcb4NDM]·3EtOH (**6**) suitable for a single crystal X-ray analysis were obtained by slow evaporation of mother liquor at room temperature and some colorless plate-like crystals suitable for single crystal X-ray diffraction, whose molecular formula has been given as [AgCl­(PPh_3_)]_4_. Furthermore, upon dissolution of these crystals in DMSO-*d*_6_, prepared for NMR studies, after slow evaporation at room temperature for a few days, new colorless prismatic crystals with formula [Ag­(PPh_3_)_3_(HAcb4NDM)]·2DMSO·H_2_O (**7**) were formed in the NMR tube.

##### 2.3.3.1 Data for [Ag­(PPh_3_)_3_(HAcb4NDM)]·3EtOH (**6**):

Yield: 0.033 g (22%), mp 150 °C. Elemental analysis found: C, 63.6; H, 5.5; N, 5.9; S, 2.5. Calc. for C_69_H_75_AgN_5_O_6_P_3_S (1303.22): C, 63.6; H, 5.8; N, 5.4; S, 2.5%. IR (ν_max_/cm^–1^): 3421 ν­(OH); 3176–3003 ν­(NH); 1705, ν­(C=O)+δ­(NH); 1616, 1585 ν­(CN+CC); 1479–998 ν­(phenyl); 1380, 1310, 1261 ν­(CS+CC); 1070 ν­(NN); 1093 ν_s_(P–C_Ph_); 848 ν­(CS); 517, 495 ν_as_(P–C_Ph_); 439 ν­(Ag–P); 252 ν­(Ag–S). FAB^+^ MS [*m*/*z*, assignment]: 631(41) [Ag­(PPh_3_)_2_]^+^; 369(8) [Ag­(PPh_3_)]^+^; 640(5), [Ag­(PPh_3_)­(HAcb4NDM)]^+^. ^1^H NMR (DMSO-*d*_6_, ppm): 12.40 (1H, N2H); 10.03 (1H, N1H); 10.03 (1H, N5H); 2.48 (3H, CH_3_); 2.96 (6H, N4CH_3_); 7.18–7.45 (PPh_3_). ^13^C­{^1^H} NMR (DMSO-*d*_6_, ppm): 185.53 (C5); 165.04 (C3); 162.51 (C1, C6); 150.18 (C7); 80.94 (C2); 16.78 (C4); 45.9 (CH_3_); 133.48, 130.15, 128.12, 129.04 (PPh_3_). ^31^P­{^1^H}P NMR (DMSO-*d*_6_, ppm): 7.13. UV–vis (λ_max_, nm): 363.

#### 2.3.4 Reaction with *N*-Piperidine-(5-acetylbarbituric)­hydrazine-1-carbothioamide (H_2_Acb4Pip)

A few yellow plate crystals suitable for a single-crystal X-ray analysis of formula [Ag­(PPh_3_)­(HAcb4Pip)]_3_·4.5H_2_O (**8**) were obtained by slow evaporation of mother liquor at room temperature.

##### 2.3.4.1 Data for [Ag­(PPh_3_)­(HAcb4Pip)]_3_·4.5H_2_O (**8**):

Yield: 0.019 g (8%), mp 250 °C. Elemental analysis Found: C, 50.7; H, 4.5; N, 9.8; S, 4.2. Calc. for C_90_H_105_Ag_3_N_15_O_13.5_P_3_S_3_ (2125.61): C, 50.8; H, 5.0; N, 9.9; S, 4.5%. IR (ν_max_/cm^–1^): 3428 ν­(OH); 3183, 3051 ν­(NH); 1711, 1659, ν­(C=O)+δ­(NH); 1611 ν­(CN+CC); 1437–1024 ν­(phenyl); 1384, 1357, 1242 ν­(CS+ CC); 1024 ν­(NN); 1095 ν_s_(P–C_Ph_); 823 ν­(CS); 522, 509 ν_as_(P–C_Ph_); 438, 414 ν­(Ag–P); 279 ν­(Ag–S). FAB^+^ MS [*m*/*z*, assignment]; 1469(3) [Ag_3_(L)_2_(PPh_3_)_2_]^+^; 1050(4) [Ag_2_(L)­(PPh_3_)_2_]^+^; 788(2) [Ag_2_(L)­(PPh_3_)]^+^; 681(5) [Ag­(HAcb4Pip)­(PPh_3_)]^+^; 631(35) [Ag­(PPh_3_)_2_]^+^; 418.(3) [Ag­(HAcb4Pip)]^+^; 369(56) [Ag­(PPh_3_)]^+^. ^1^H NMR (DMSO-*d*_6_, ppm): 14.56 (1H, N2H); 10.22 (1H, N1H); 10.22 (1H, N5H); 1.33 (4H,Hb); 1.46 (4H, Hc); 2.52 (3H, Me); 7.24–7.50 (PPh_3_). ^13^C­{^1^H} NMR (DMSO-*d*_6_, ppm): 165.21 (C3); 159.00 (C1, C6); 150.30 (C7); 86.44 (C2); 16.85 (C4); 48.11 (Ca); 25.26 (Cb); 24.55 (*Cc*); 133.55, 131.44, 130., 129.27 (PPh_3_). ^31^P­{^1^H} NMR (DMSO-*d*_6_, ppm): 14.02. UV–vis (λ_max_, nm); 364.

#### 2.3.5 Reaction with *N*-Hexamethyleneiminyl-(5-acetylbarbituric)­hydrazine-1-carbothioamide (H_2_Acb4Hexim)

A few yellow plate crystals of [Ag­(PPh_3_)_3_(HAcb4Hexim)]·3EtOH·3H_2_O (**9**) suitable for a single-crystal X-ray analysis were obtained by slow evaporation of mother liquor at room temperature.

##### 2.3.5.1 Data for [Ag­(PPh_3_)_3_(HAcb4Hexim)]·3EtOH·3H_2_O (**9**):

Yield: 0.049 g (31%), mp 135 °C. Elemental analysis Found: C, 63.0; H, 6.5; N, 4.7; S, 2.5. Calc. for C_73_H_87_AgN_5_O_9_P_3_S (1409.44): C, 62.1; H, 6.2; N, 5.0; S, 2.3%. IR (ν_max_/cm^–1^): 3423 ν­(OH); 3173, 3002 ν­(NH); 1710, 1620 ν­(C=O)+δ­(NH); 1476 ν­(CN+CC); 1455–998 ν­(phenyl); 1384, 1268 ν­(CS+CC); 1036 ν­(NN); 1095 ν_s_(P–C_Ph_); 804 ν­(CS); 518, 490 ν_as_(P–C_Ph_); 412 ν­(Ag–P); 269 ν­(Ag–S). FAB^+^ MS [*m*/*z*, assignment]: 694(8) [Ag­(HAcb4Hexim)­(PPh_3_)]^+^; 631(75) [Ag (PPh_3_)_2_]^+^; 432(1) [Ag­(HAcb4Hexim)]^+^; 369(67) [Ag­(PPh_3_)]^+^. ^1^H NMR (MeOH-*d*_4_, ppm): 14.49 (1H, N2H); 10.0 (1H, N1H); 10.0 (1H, N5H); 3.63 (4H, Ha); 1.60 (4H, Hc); 2.40 (3H, Me); 7.08–7.50 (PPh_3_). ^31^P­{^1^H} NMR (MeOH-*d*_4_, ppm): 10.82, 34.5. UV–vis (λ_max_, nm): 368.

### 2.4 Crystal Structure Determination and Refinement

Diffraction data were obtained at 100(2) K, using Bruker X8 Kappa APEXII or Bruker SMART CCD 1000 diffractometers from crystals mounted on glass fibers and Mo*K*α radiation (λ = 0.71073 Å). The data were processed with APEX2 ^22^ and corrected for absorption using a multiscan type.^23^ The structures were solved by direct methods^24^ which revealed the positions of all non-hydrogen atoms. These were refined on *F* ^2^ by a full-matrix least-squares procedure using anisotropic displacement parameters.^25^ All hydrogen atoms were located on difference maps, and the positions of O–H and N–H hydrogen atoms were refined (others were included as riders); the isotropic displacement parameters of H atoms were constrained to 1.2/1.5 *U*_eq_ of the carrier atoms. Molecular graphics were generated using DIAMOND.^26^ Crystal data, experimental details, and refinement results are summarized in Tables 1 and 2a,b.

**Table 1:** Crystallographic and Structural Data for Cited Compounds

Compound	{[Ag(H_2_O)_14_](NO_3_)}_n_	[Ag(PPh_3_)_4_](NO_3_)·EtOH	[AgCl(PPh_3_)]_4_
Empirical formula	H_28_AgN_2_O_20_	C_74_H_66_AgNO_4_P_4_	C_72_H_60_Ag_4_Cl_4_P_4_
Formula weight	484.11	1265.02	1622.36
Crystal system	Orthorhombic	Monoclinic	Orthorhombic
Space group	*Pbca* (No. 60)	*C*2*/c* (No. 15)	*Pbcn* (No. 60)
Unit cell dimensions			
*a*/Å	6.9670(3)	17.5559(10)	17.8022(7)
*b*/Å	14.4797(5)	20.8342(12)	20.5515(8)
*c*/Å	19.3143(7)	20.1627(12)	18.0794(5)
α/°	90	90	90
β/°	90	106.834(3)	90
γ/°	90	90	90
Volume/Å^–3^	1948.43(13)	7058.7(7)	6614.6(4)
Z	4	4	4
Calc. density/Mg/m^3^	1.650	1.190	1.629
Absorp. coefc./mm^–1^	1.122	0.422	1.467
*F*(000)	996	2624	3232
Crystal size	0.43 × 0.23 × 0.05	0.24 × 0.15 × 0.10	0.23 × 0.11 × 0.09
θ range/°	2.11–26.42	1.55–26.02	1.51–26.02
Limiting indices/*h,k,l*	0/8, 0/18, 0/24	–21/20, 0/25, 0/24	0/21, 0/25, 0/22
Refl. collect/unique, *R*_int_	2000/2000, 0.0527	6947/6947, 0.0788	6527/6527, 0.0627
Max./min transm.	1.000/0.527	1.000/0.824	1.000 and 0.862
Data/parameters	2000/118	6947/404	6527/379
Goodness-of-fit on *F* ^2^	1.067	1.025	1.061
Final *R* indices	*R*_1_ = 0.0349	*R*_1_ = 0.0603	*R*_1_ = 0.0394
	*wR*_2_ = 0.1199	*wR*_2_ = 0.1494	*wR*_2_ = 0.0561
*R* indices (all data)	*R*_1_ = 0.0417	*R*_1_ = 0.0957	*R*_1_ = 0.0733
	*wR*_2_ = 0.1264	*wR*_2_ = 0.1617	*wR*_2_ = 0.0675
Largest dif. peak/hole, e.Å^–3^	0.354/–0.786	0.437/–1.456	0.714/–0.558
CCDC number	2,454,019	2,454,020	2,454,021

**Table 2:** Crystallographic and Structural Data for Cited Compounds

Compound	[Ag(PPh_3_)_3_(HAbc4NH_2_)]·EtOH·H_2_O (**2**)	[Ag(PPh_3_)_3_(HAbc4NHM)]·2EtOH (**4**)	[Ag(PPh_3_)_3_(HAbc4NHM)]·2EtOH (**4a**)
Empirical formula	C_63_H_61_AgN_5_O_5_P_3_S	C_66_H_67_AgN_5_O_5_P_3_S	C_66_H_67_AgN_5_O_5_P_3_S
Formula weight	1201.01	1243.09	1243.08
Crystal system	Triclinic	Triclinic	Triclinic
Space group	*P*1̅ (No.2)	*P*1̅ (No.2)	*P*1̅ (No.2)
Unit cell dimensions			
*a*/Å	13.9323(6)	12.9006(3)	12.8969(3)
*b*/Å	18.4058(9)	13.3277(3)	13.3233(3)
*c*/Å	27.1167(17)	18.6431(5)	18.6500(4)
α/°	100.786(3)	73.380(2)	73.358(1)
β/°	95.691(3)	84.259(2)	84.278(1)
γ/°	112.036(2)	77.618(2)	77.617(1)
Volume/Å^–3^	6221.3(6)	2997.49(13)	2996.44(12)
*Z*	4	2	2
Calc. density/Mg/m^3^	1.282	1.377	1.378
Absorp. coefc./mm^–1^	0.485	0.506	0.506
*F*(000)	2488	1292	1292
Crystal size	0.24 × 0.10 × 0.10	0.41 × 0.17 × 0.07	0.49 × 0.18 × 0.05
θ range/°	0.78–25.35	1.62–26.02	1.62–26.02
Limiting indices/*h,k,l*	– 16/16, – 22/21, 0/32	– 15/15, – 15/16, 0/23	– 15/15, – 15/16, 0/23
Refl. collect/unique, *R*_int_	22765/22765, 0.0694	11775/11775, 0.0661,	11757/11757, 0.0602
Max./min transm.	1.000/0.912	1.000/0.855	1.000/0.909
Data/parameters	22765/1311	11775/730	11757/730
Goodness-of-fit on *F* ^2^	1.036	1.036	1.026
Final *R* indices	*R*_1_ = 0.0951, *wR*_2_ = 0.2473	*R*_1_ = 0.0587, *wR*_2_ = 0.1042	*R*_1_ = 0.0433, *wR*_2_ = 0.0739
*R* indices (all data)	*R*_1_ = 0.1594, *wR*_2_ = 0.2940	*R*_1_ = 0.0941, *wR*_2_ = 0.1154	*R*_1_ = 0.0690, *wR*_2_ = 0.0830
Largest dif. peak/hole, e.Å^–3^	3.411/–2.033	0.819/–1.017	0.879/–0.862
CCDC number	2,454,022	2,454,013	2454014

Compound	[Ag(PPh_3_)_3_(HAbc4NDM)]·3EtOH (**6**)	[Ag(PPh_3_)_3_(HAbc4NDM)]·2DMSO·H_2_O (**7**)	[Ag(PPh_3_)(HAcb4Pip)]_3_·4.5H_2_O (**8**)	[Ag(PPh_3_)_3_(HAcb4Hexim)]·3EtOH·3H_2_O (**9**)
Empirical formula	C_69_H_75_AgN_5_O_6_P_3_S	C_67_H_71_AgN_5_O_6_P_3_S_3_	C_90_H_102_Ag_3_N_15_O_13.5_P_3_S_3_	C_73_H_86_AgN_5_O_9_P_3_S
Formula weight	1303.18	1339.24	2122.56	1410.30
Crystal system	Triclinic	Triclinic	Monoclinic	Triclinic
Space group	*P*1̅ (No.2)	*P*1̅ (No.2)	*P*2_1_/*c* (No. 14)	*P*1̅ (No.2)
Unit cell dimensions				
*a*/Å	13.0891(5)	13.4013(3)	25.989(6)	13.6018(5)
*b*/Å	14.0879(4)	14.2832(3)	19.867(4)	13.8762(5)
*c*/Å	19.5506(7)	18.2919(4)	20.650(4)	20.0603(7)
α/°	109.460(2)	67.6220(10)	90	102.503(2)
β/°	96.281(2)	88.5090(10)	90.742(3)	103.453(2)
γ/°	103.173(2)	79.0640(10)	90	95.068(2)
Volume/Å^–3^	3241.6(2)	3174.72(12)	10661(4)	3556.4(2)
*Z*	2	2	4	2
Calc. density/Mg/m^3^	1.335	1.401	1.322	1.317
Absorp. coefc./mm^–1^	0.472	0.548	0.711	0.439
*F*(000)	1360	1392	4356	1478
Crystal size	0.21 × 0.20 × 0.07	0.45 × 0.34 × 0.15	0.43 × 0.10 × 0.06	0.19 × 0.19 × 0.02
θ range/°	1.58–26.02	1.26–26.02	1.42–22.09	1.56–23.26
Limiting indices/*h,k,l*	–16/15, – 17/16, 0/24	–16/16, – 15/17, 0/22	–27/27, 0/20, 0/21	–15/14, – 15/15, 0/22
Refl. collect/unique	12764/12764, 0.0612	12515/12515, 0.0652	13171/13171, 0.0561	10207/10207, 0.0748
Max./min transm.	1.000–0.938	1.000–0.865	1.000–0.774	1.000–0.861
Data/parameters	12764/766	12515/766	13171/1128	10207/752
Goodness-of-fit on *F* ^2^	1.046	1.028	1.093	1.056
Final *R* indices	*R*_1_ = 0.0560, *wR*_2_ = 0.1327	*R*_1_ = 0.0450, *wR*_2_ = 0.0844	*R*_1_ = 0.0695, *wR*_2_ = 0.1896	*R*_1_ = 0.0917, *wR*_2_ = 0.2385
*R* indices (all data)	*R*_1_ = 0.0882, *wR*_2_ = 0.1505	*R*_1_ = 0.0665, *wR*_2_ = 0.0933	*R*_1_ = 0.0967, *wR*_2_ = 0.2071	*R*_1_ = 0.1401, *wR*_2_ = 0.2781
Largest dif. peak/hole, e.Å^–3^	1.886/–1.218	0.522/–0.892	2.141/–0.842	3.839/–1.310
CCDC number	2,454,015	2,454,016	2,454,017	2,454,018

### 2.5 Theoretical Methods

The calculations of noncovalent interactions were carried out using Gaussian-16 ^27^ program at the PBE0-D3/def2-TZVP level of theory.^28–30^ The Grimme’s D3 dispersion correction has been used in the calculations^28^ since it is adequate for the correct evaluations of noncovalent interactions. To gain insight into the noncovalent interactions observed in the solid state, we performed DFT calculations on molecular clusters extracted from the crystallographic structures. These finite assemblies reproduce the key H-bonded interactions identified in the crystal lattice. The geometries were taken directly from the X-ray structures, and only the hydrogen positions involved in H-bonds were optimized to better reflect realistic interaction geometries. To improve computational efficiency, simplified models of the original compounds were used in the theoretical study. For instance, a supramolecular dimer of compound **8** comprises over 400 atoms. To reduce computational cost without significantly affecting the key noncovalent interactions, the triphenylphosphine ligands were substituted with trimethylphosphine. This modification is expected to have a negligible impact on the hydrogen-bonding interactions that define, for instance, the $R_{2}^{2}$ ­(8) motifs. The interaction energies were computed by calculating using the approach developed by Espinosa et al.^31^ The QTAIM analysis^32^ has been computed at the same level of theory by means of the AIMAll program.^33^

## 3 Results and Discussion

### 3.1 Synthesis and Characterization

In general, the reactions of Ag­(NO_3_) with thiosemicarbazones derived from 5-acetylbarbituric acid, in the presence of an excess of triethylamine to induce its deprotonation, gave insoluble products which could not be crystallized by the usual methods nor easily identified. However, when such reactions were carried out under the same conditions but in the presence of triphenylphosphine, series of Ag­(I) compounds with different nuclearities were obtained. For example, the reaction between 5-acetylbarbituric hydrazine-1-carbothioamide and Ag­(NO_3_) in a 1:1 molar ratio in the presence of PPh_3_ gives compounds with different phosphine contents of formulas such as Ag­(PPh_3_)_2_(HAcb4NH_2_)], [Ag­(PPh_3_)_3_(HAcb4NH_2_)]·EtOH·H_2_O and [Ag­(PPh_3_)_4_]­(NO_3_)·EtOH, and {[Ag­(H_2_O)_14_]­(NO_3_)}_n_ as byproduct. Something similar occurs in the reaction of *N*-methyl-(5-acetylbarbituric)­hydrazine-1-carbothioamide in AgNO_3_:H_2_Acb4NHM:PPh_3_, using 1/1/1 and 1/2/1 molar ratios. However, this behavior was not observed in reactions with 4N-disubstituted 5-acetylbarbituric thiosemicarbazones. The coexistence of several species with different PPh_3_ contents has previously been observed in Ag­(I) compounds with sulfanyl carboxylates.^34^

Slow evaporation of the mother liquors at room temperature and in the absence of light allowed obtaining crystals of mononuclear primary complexes, [Ag­(PPh_3_)_3_(HAcb4NH_2_)]·EtOH·H_2_O, [Ag­(PPh_3_)_3_(HAcb4NHM)]·2EtOH, [Ag­(PPh_3_)_3_(HAcb4NDM)]·3EtOH and [Ag­(PPh_3_)_3_(HAcb4Hexim)]·3EtOH·3H_2_O, and trinuclear [Ag­(PPh_3_)­(HAcb4Pip)]_3_·4.5H_2_O, suitable for structural analysis by single crystal X-ray diffraction, and [Ag­(PPh_3_)_2_(HAcb4NH_2_)], [Ag­(PPh_3_)­(H_2_Acb4NHM)]­(NO_3_)·2H_2_O and [Ag­(PPh_3_)­(H_2_Acb4NHM)_2_]­(NO_3_)·3H_2_O as secondary products, as well as [Ag­(PPh_3_)_3_(HAcb4NDM)]·2DMSO·H_2_O from solutions of [Ag­(PPh_3_)_3_(HAcb4NDM]·3EtOH in DMSO-*d*_6_, prepared for an NMR study of this compound, together with {[Ag­(H_2_O)_14_]­(NO_3_)}_n_, [Ag­(PPh_3_)_4_]­(NO_3_)·EtOH and [AgCl­(PPh_3_)]_4_, as minor products.

Polycrystalline samples of compounds were characterized using elemental analysis and infrared spectroscopy. Four of them (**4**, **6**, **8**, and **9**) were also characterized by mass spectrometry. However, the low solubility of the complexes in common solvents prevented characterization by ^1^H, ^13^C­{^1^H}, and ^31^P­{^1^H} NMR spectroscopy, except for compounds **3**, **5**, **6**, **8**, and **9**. See Electronic Supporting Information.

The presence of signals of different fragments with PPh_3_ and the corresponding thiosemicarbazone in the FAB^+^ mass spectra of the complexes derived from 4N-mono- or -disubstituted thiosemicarbazones indicates easy cleavage of Ag–P and Ag–S bonds. Furthermore, the appearance of peaks at *m*/*z* 1469, 1050 and 788, corresponding to [Ag_3_(L)_2_(PPh_3_)_2_]^+^, [Ag_2_(L)­(PPh_3_)_2_]^+^ and [Ag_2_(L)­(PPh_3_)]^+^, respectively, in [Ag­(PPh_3_)­(HAcb4Pip)]_3_ 4.5H_2_O suggests the formation of polynuclear species (Figures S1 and S2).

In the complexes **6**, **8**, and **9**, the electronic spectra display a single band between 360 and 370 nm, which can be attributed to *n*-π* ligand transitions.

### 3.2 FT-IR Spectra

The FTIR spectra of Ag­(I)-tsc complexes showed bands in the ranges of 3420, 3324, 3181, and 3023 cm^–1^ (due to OH, – NH_2_ and – NH, respectively). Appearance of the characteristic bands of ν­(C=S) and ν­(C=N) vibrational modes at 780–850 and 1476–1611 cm^–1^ respectively, indicates the presence of the thiosemicarbazone ligands. Shifting of ν­(C=S) and ν­(C=N) bands to lower energy in the complex compared to the free ligand (830 and 1570–1627 cm^–1^, respectively) confirmed that the tsc ligands are coordinated to silver­(I) by S-thiolate donor atom.^7^

In the IR spectra of the complexes, a weak band at 1093 cm^–1^ has been attributed to the symmetric ν_s_(P–C) stretching modes, and two or three strong bands between 525 and 490 cm^–1^ have been ascribed to the antisymmetric ν_as_(P–C) stretching modes, indicating coordination of PPh_3_ via the P atom to the silver­(I) ion.^35^ The bands in free triphenylphosphine are observed at 1089, 512, 492, and 489 cm^–1^, respectively.^36^ Furthermore, in the far-infrared spectra of the complexes, a band at 430–410 cm^–1^ has been assigned to ν­(Ag–P) and a weak band between 270 and 250 cm^–1^ has been attributed to ν­(Ag–S).^37^

Finally, the ν­(N–O) asymmetric stretching mode of the nitrate anion appeared at 1398 cm^–1^ in the 4*N*-methyl derivative complexes **3** and **5,** and in [Ag­(PPh_3_)_4_]­(NO_3_)·EtOH.^38^ In general, the complexes containing the nitrate ion exhibit the asymmetric N–O stretching modes of NO_3_^–^ as a medium-intensity band within the range of approximately 1400–1300 cm^–1^. The presence of a single band indicates the existence of the ionic nitrate, NO_3_^–^,^39^ whereas the splitting of this band at 1490–1639 cm^–1^ and 1270–1290 cm^–1^ unlocks the coordination of the nitrate as an unidentate ligand ONO_2_^–^. In both scenarios, the strong absorptions due to the thiosemicarbazone and phosphine ligands frequently overlap the N–O stretching vibrations of the nitrates.^40^ Consequently, the coordination mode cannot be determined with certainty (Figures S3–S6).

### 3.3 NMR Spectra

Nuclear magnetic resonance (NMR) spectra of the complexes with sufficient solubility in DMSO were recorded, and the resulting data are presented in the experimental section and in the figures, as detailed in the electronic Figures S7–S13. The presence of the individual ligands in each of the complexes was confirmed by the signals observed in the ^1^H, ^13^C­{^1^H} and ^31^P­{^1^H} NMR spectra. In the ^1^H NMR spectra in DMSO, the N2H signal of the thiosemicarbazone ligands could be identified, occurring between 14.38 and 14.56 ppm in the 4*N*-methyl, piperidyl and hexamethyl complexes. A downfield shift with respect to the position of the free 5-acetylbarbituric thiosemicarbazones (13.1–13.6 ppm) was observed, with an upfield shift reaching 12.40 ppm in the 4N-dimethyl complex. Moreover, the signal attributed to the protons of the acetyl group, which in the 4N-disubstituted thiosemicarbazones occurs at 16.1–16.60 ppm, is also shifted upfield in the complexes. The observed deshielding of these signals provides evidence that the ligands are undergoing coordination. In the ^13^C­{^1^H} NMR of the coordinated thiosemicarbazones, the signals of the carbon groups can be clearly discerned at 150.18 and 150.33 ppm, as well as at 162.31 and 159.00 ppm. Additionally, the thiolate carbon can be identified at 185.53 ppm. The shift of this last signal to a lower field is a consequence of coordination through the sulfur atom of the thiocarbonyl group. The ^31^P­{^1^H} NMR spectra of the complexes exhibited a single peak, attributable to PPh_3_ coordinated, within the range of 7.13 to 14.02 ppm in DMSO (−3.64 for free PPh_3_). This peak was comparable to those observed in previous literature reports on silver­(I) triphenylphosphine derivatives.^34^ A second signal at 34.5 ppm emerged in the ^31^P­{^1^H} NMR spectrum of N-hexamethyleneiminyl-(5-acetylbarbituric)­hydrazine-1-carbothioamide, which can be attributed to the formation of the triphenylphosphine oxide.^41^

The room temperature ^31^P­{^1^H} NMR spectra show a singlet at 5.3–9.0 ppm and this is due to the coordinated PPh_3_ ligand; a weak singlet near to 31.5 ppm indicates the formation of triphenylphosphine oxide^42,43^ generated by the oxidation of some of the released triphenylphosphine. The same phenomenon was previously found for [Ag­(PPh_3_)_3_(Hfspa)].^44^

### 3.4 Molecular Structures and Supramolecular Analysis

The solid-phase molecular structure of [Ag­(PPh_3_)_2_(HAcb4NDH)] (**1**) was investigated using analytical and IR spectral data, which allowed estimation of the coordination of the Ag­(I) ion. It was determined that the Ag­(I) ion is coordinated by two phosphorus atoms from two triphenylphosphine molecules and the thiolate sulfur atom from the monodeprotonated thiosemicarbazone [HAbc4NH_2_]^−^, completing a trigonal coordination around the metal center (Chart [Fig chart2]a). Furthermore, there is probable interaction between silver­(I) and the azomethine nitrogen atom, which, if strong enough, would give rise to a distorted tetrahedral coordination geometry (Chart [Fig chart2]b). Conversely, if the Ag···N interaction were weak, the geometry should be described as trigonal pyramidal rather than trigonal planar.^45,46^ Examples of tetrahedral structures with the AgP_2_SN^45^ core or trigonal structures with the AgP_2_S core^47,48^ have been published. In addition, there is evidence from the literature of a 3-coordinate cationic silver­(I) complex, with a trigonal planar structure consisting of an AgNP_2_ core.

**2 chart2:**
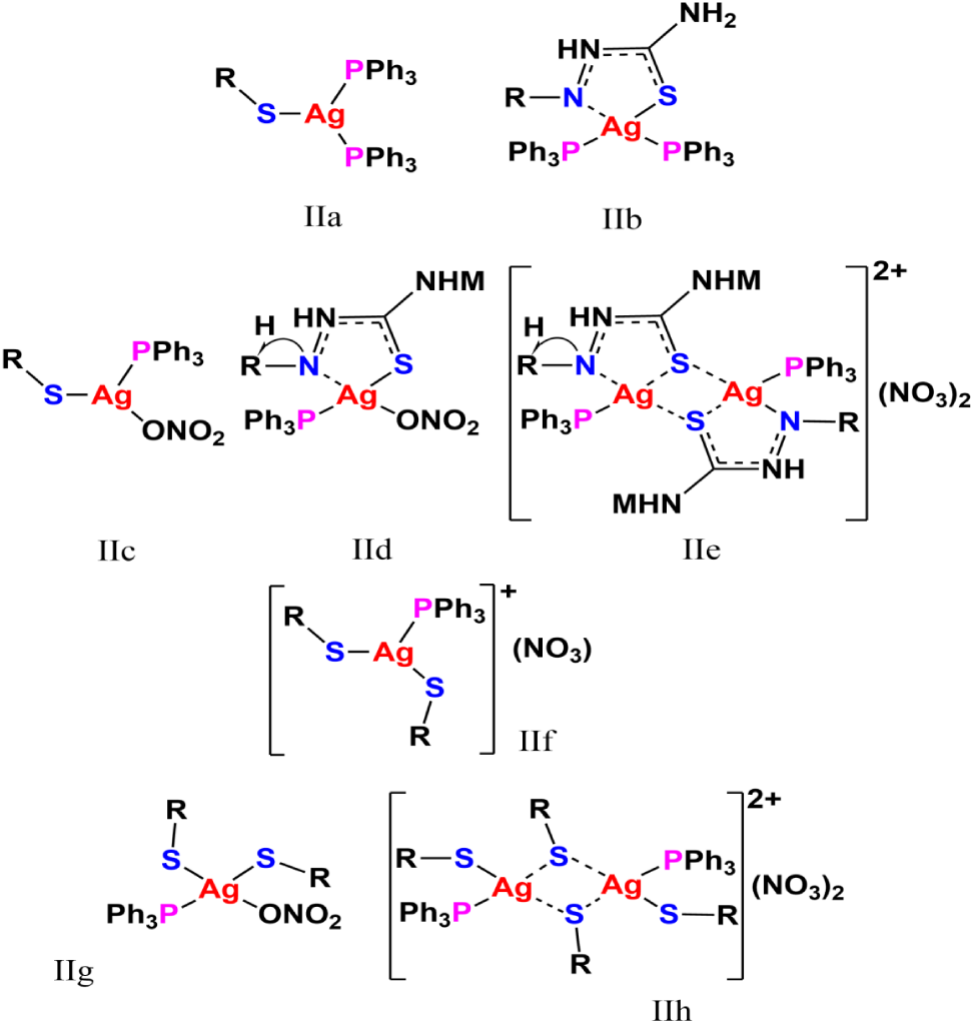
Schematic depiction of potential coordination geometry for complexes **1**, **3** and **5**

When it comes to describing the coordination geometry of Ag­(PPh_3_)­(H_2_Acb4NHM)­(NO_3_)·2H_2_O (**3**), we should consider two proposals. One of these involves the silver­(I) ion bonded to a thiocarbonyl sulfur atom of a neutral thiosemicarbazone molecule, to a phosphorus atom of a triphenylphosphine ligand, and to an oxygen atom of the weakly coordinated nitrate anion, forming a trigonal AgPSO core (Chart [Fig chart2]c). Alternatively, the metal center can be considered in a distorted tetrahedral AgPOS,N environment, taking into account the interaction between the silver­(I) ion and the azomethine nitrogen atom of the thiosemicarbazone (Chart [Fig chart2]d).^40^ In addition, a proposal is made based on the formation of cationic units [Ag­(PPh_3_)­(H_2_AbcNHM)]_2_^2+^, which could be formed from monomers connected by Ag–S bonds (Chart [Fig chart2]e).^49^ However, in these last two proposals the thiosemicarbazone should be found in its keto-neutral tautomeric form^21^ and, consequently, the ^1^H NMR spectrum should present a signal at 2.5 ppm corresponding to C6H (Chart I), which is not observed.

In compound Ag­(PPh_3_)­(H_2_Acb4NHM)_2_(NO_3_)·3H_2_O (**5**), the silver­(I) ion can be coordinately bonded by two thiocarbonyl sulfur atoms from two neutral thiosemicarbazones and one phosphorus atom. This would correspond to a cationic complex with a trigonal coordination geometry and an NO_3_^–^ outside the coordination sphere (Chart [Fig chart2]f). Alternatively, the nitrate may be weakly O-coordinated in a distorted tetrahedral coordination environment (Chart [Fig chart2]g).^46^ Finally, a cationic dimer structure can also be considered, where each silver atom is coordinated to two thione sulfur atoms from two different neutral thiosemicarbazones, forming bridges between two Ag^+^ ions, with the formation of an Ag_2_S_2_ core. Furthermore, each metal center completes its distorted AgS_3_P tetrahedral coordination with the phosphorus atom from one triphenylphosphine and the sulfur atom from another thiosemicarbazone (Chart [Fig chart2]h).^49^

#### 3.4.1 Structures of {[Ag­(H_2_O)_14_]­(NO_3_)}_n_, [Ag­(PPh_3_)_4_]­(NO_3_)·EtOH, and [AgCl­(PPh_3_)]_4_

The crystallographic and refinement data are compiled in Table 1, while a selection of bond distances and angles is presented in Table S1. The structure of **{[Ag­(H**_**2**_ **O)**_**14**_ **]­(NO**_**3**_)}_**n**_ has been determined to crystallize in the orthorhombic system, space group *Pbca*. The asymmetric unit is constituted by two Ag^+^ ions, with an occupancy factor of 0.25, by seven coordinated water molecules, one of which acts as a bridge between both silver ions, and by a nitrate ion that remains bound to one of the molecules by means of a hydrogen bond O6–H···O11 (Figure 1a). The oxygen atom of this molecule, O6, in turn, forms a hydrogen bond as acceptor with another water molecule coordinated to the second Ag^+^ ion, O2–H···O6, forming a homosynthon of graph set $R_{1}^{1}$ ­(6). The coordination around each Ag^+^ ion is distorted octahedral (Figure 1b), and the Ag–O bond distances range between 2.3513(15) and 2.4735(15) Å, while the *trans* O–Ag–O bond angles range from 163.69(6)° to 177.85(6)°, and the *cis* ones vary between 80.70(5)° and 98.37(5)°.

**Figure 1:**
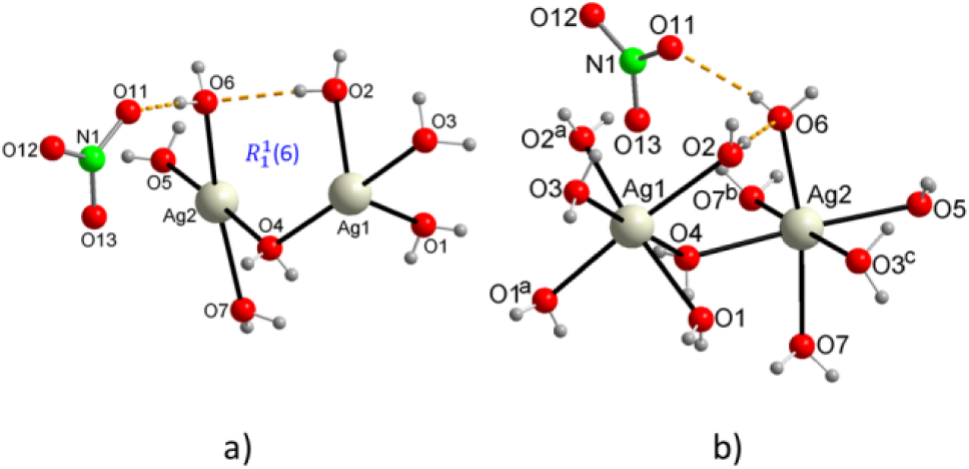
(a) The asymmetrical unit, as well as, (b) the local coordination environment surrounding silver in {[Ag­(H_2_O)_14_]­(NO_3_)}_n_.

In the crystal packing, the [Ag­(OH_2_)_6_]^+^ octahedral of Ag1 ions share two continuous vertices and are symmetrically related to the nearest neighboring octahedral in the direction of the crystallographic axis “a”, forming chains in this direction (Figure 2a), where the Ag1–Ag1 distances are 3.5221(3) Å. The [Ag­(OH_2_)_6_]^+^ octahedral of the Ag2 ions also form centrosymmetric dimer units, where the Ag2–Ag2 distances are 3.3691(14) Å, and they join the Ag1 dimers by simple OH_2_ bridges in the direction parallel to the “c” axis, with Ag1–Ag2 distances of 4.3262 (10) Å, forming sheets parallel to the “ac” plane, stacked at distances of 7.2398(5) Å. In each sheet there are numerous hydrogen bonds that reinforce its structure (Figure 2a). In addition, the interlayer space is occupied by NO_3_^–^ ions, whose oxygen atoms are hydrogen bond acceptors with the water molecules of the upper and lower layers, like sandwiches (Figure 2b), thus maintaining a very rigid 3D structure.

**Figure 2:**
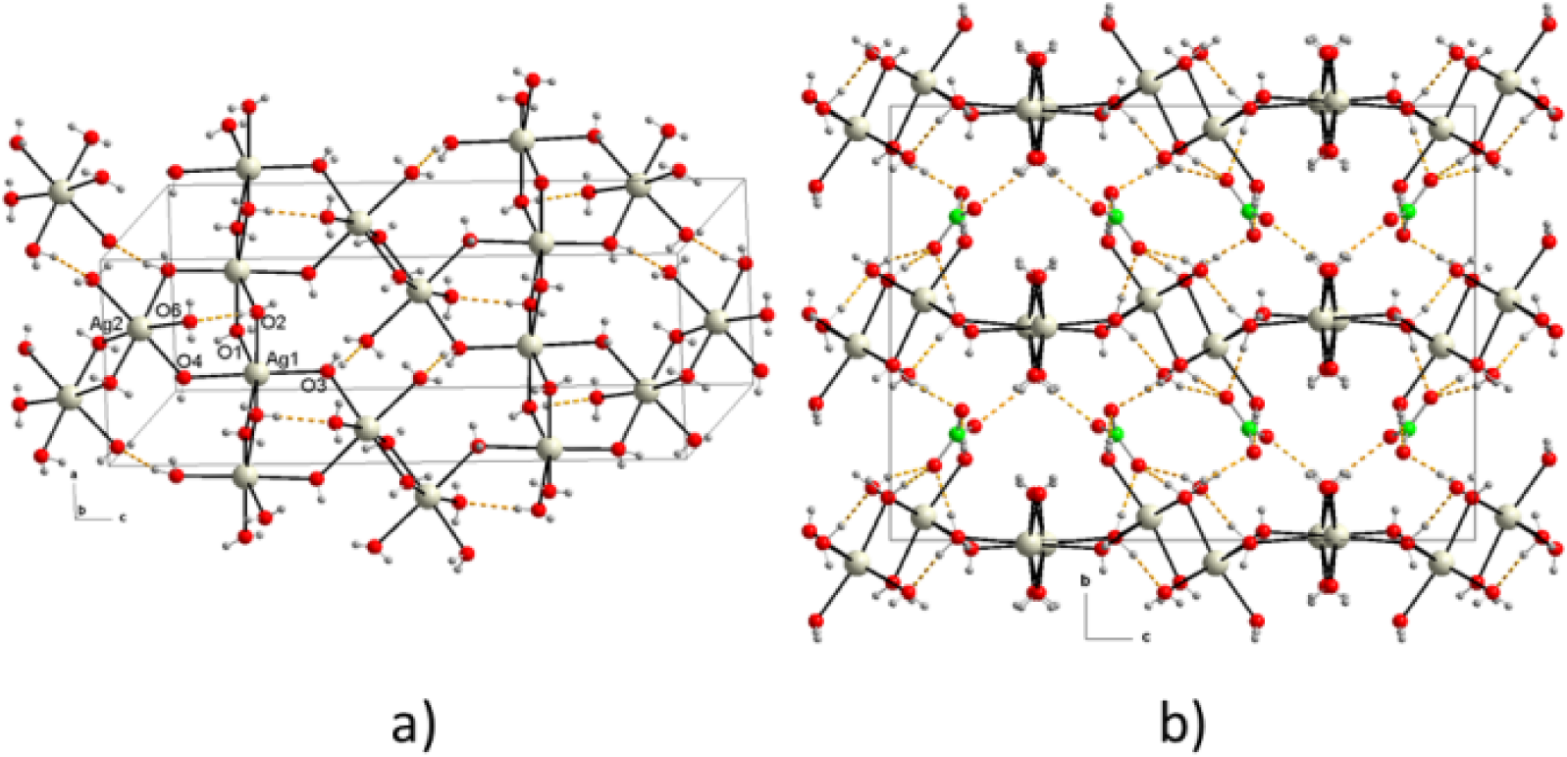
Packing diagram of {[Ag­(H_2_O)_14_]­(NO_3_)}_n_, (a) perpendicular to the [1 0 1] plane, and (b) perpendicular to the [0 1 1] plane.

The structure of **[Ag­(PPh**_**3**_)_**4**_ **]­(NO**_**3**_ **)·EtOH** has been determined. This solvated salt is a solvopolymorph of [Ag­(PPh_3_)_4_]­(NO_3_)^50^ and is isostructural with a series of compounds containing the [Ag­(PPh_3_)_4_]^+^ cation associated with a monobasic anion, such as BF_4_−],^51,52^ ClO_4_–^53^ ReO_4_^–^,^54^ PF_6_^–^,^55^ SbF_6_^–^,^56,57^ SO_3_CF_3_^–^,^58^ CF_3_CO_2_^–^,^59^ the majority of which are known to crystallize in the *R*3̅ space group. The compound under current study is isotypic with [Ag­(PPh_3_)_4_]­(HCO_3_)·2EtOH·3H_2_O^60^ and crystallizes in the monoclinic space group *C*2/*c*. Silver ions are located in special positions, on a 2-fold rotation axis, coordinated by two independent PPh_3_, so that two other symmetrically related phosphines complete the tetrahedral coordination environment of the cations. The nitrogen atom of the nitrate anion and the oxygen atom of the ethanol solvate are located in special positions, also on a 2-fold rotation axis. Consequently, both species are disordered about two symmetrically related positions, with occupancy factors of 50%, for both the oxygen atoms of the nitrate and for the carbon and hydrogen atoms of the ethanol (Figure 3a). The angular environment surrounding Ag^+^ ions is a good approximation to the ideal tetrahedron, with P–Ag–P angles ranging from 108.12° between symmetrically independent phosphines to 110.03–110.45° between symmetrically related ones. The Ag–P bond distances range from 2.6001(12) Å to 2.6148(11) Å and are nearly identical to those found in [Ag­(PPh_3_)_4_]­(HCO_3_) 2EtOH 3H_2_O,^60^ but significantly shorter than those in [Ag­(PPh_3_)_4_]­(NO_3_)^50^ and most other isomorphs. In crystal packing, classical hydrogen bonds have not been found, but cations, anions and solvent molecules are involved in weak nonclassical C–H···O hydrogen bonds.

**Figure 3:**
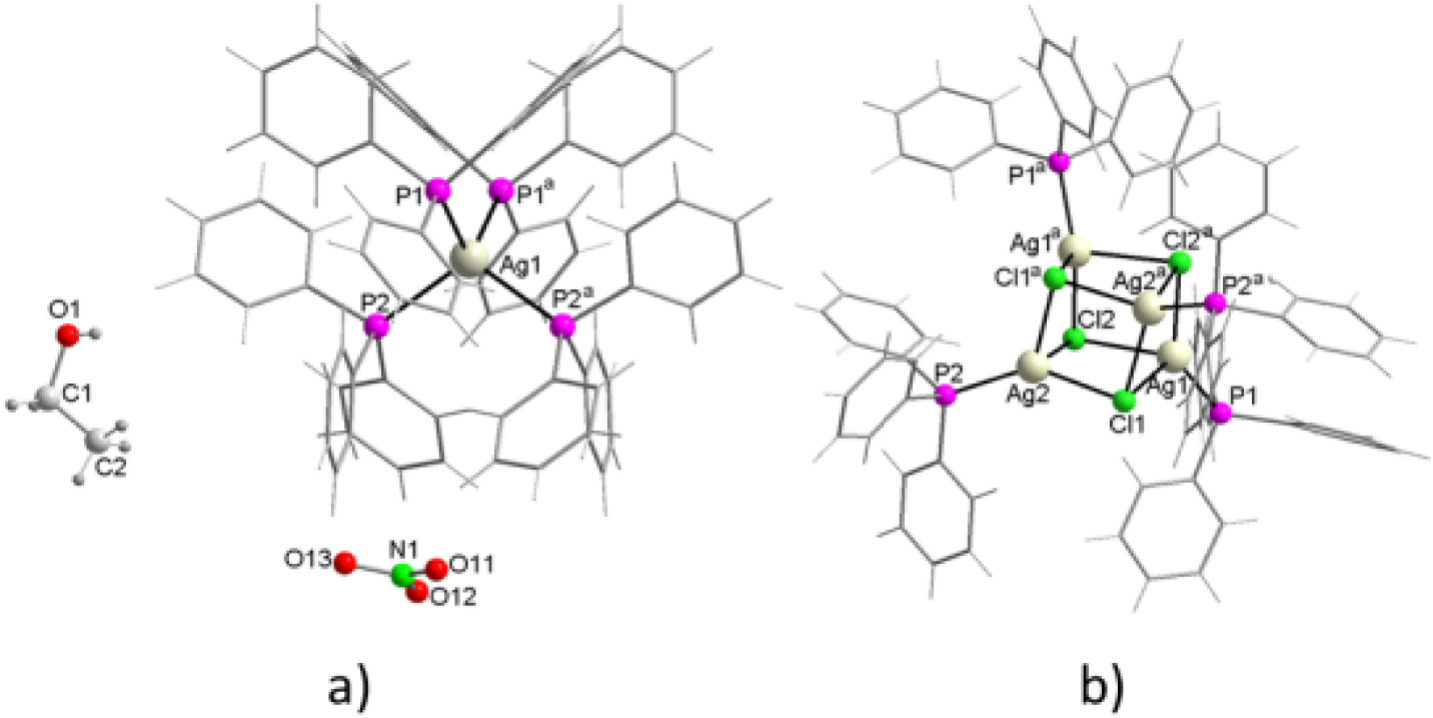
(a) Molecular structure of [Ag­(PPh_3_)_4_]­(NO_3_)·EtOH, (b) molecular structure of [AgCl­(PPh_3_)]_4_.

During the crystallization process of Ag­(I) complexes with N,N-dimethyl-(5-acetylbarbituric)­hydrazine-1-carbothioamide, the formation of colorless crystals in the form of plates between the crystals of the complex [Ag­(PPh_3_)_3_(HAcb4NDM)]·3EtOH was observed. The molecular structure of these crystals, as determined by X-ray diffraction analysis, revealed the presence of **[AgCl­(PPh**_**3**_)]_**4**_ (Figure 3b). It is hypothesized that these crystals originated from the initial AgNO_3_ sample, which was contaminated with chlorides. It should be noted that this complex was previously generated from the reaction of silver chloride with triphenylphosphine and described^61^ and its structure has also been described on a few other occasions.^62,63^

#### 3.4.2 Structures of [Ag (PPh_3_)_3_(HAcb4NHM)]·2EtOH] (**4**), [Ag­(PPh_3_)_3_(HAcb4NDM)]·3EtOH (**6**), [Ag­(PPh_3_)_3_(HAcb4NDM)]·2DMSO·H_2_O (**7**), and [Ag­(PPh_3_)_3_(HAcb4Hexim)]·3EtOH·3H_2_O (**9**)

Compounds **4**, **6**, **7** and **9** are isostructural, although the y differ in the presence of different crystallization molecules in their respective networks. The crystallographic data for these compounds are given in Table 2. All the complexes are mononuclear and in each the silver ion is coordinated by three phosphorus atoms from three triphenylphosphines and by the thiolate sulfur atom of the corresponding monodeprotonated thiosemicarbazone, resulting in a distorted tetrahedral coordination around the silver ion (Figure 4). The coordination environment does not vary significantly from one complex to another.

**Figure 4:**
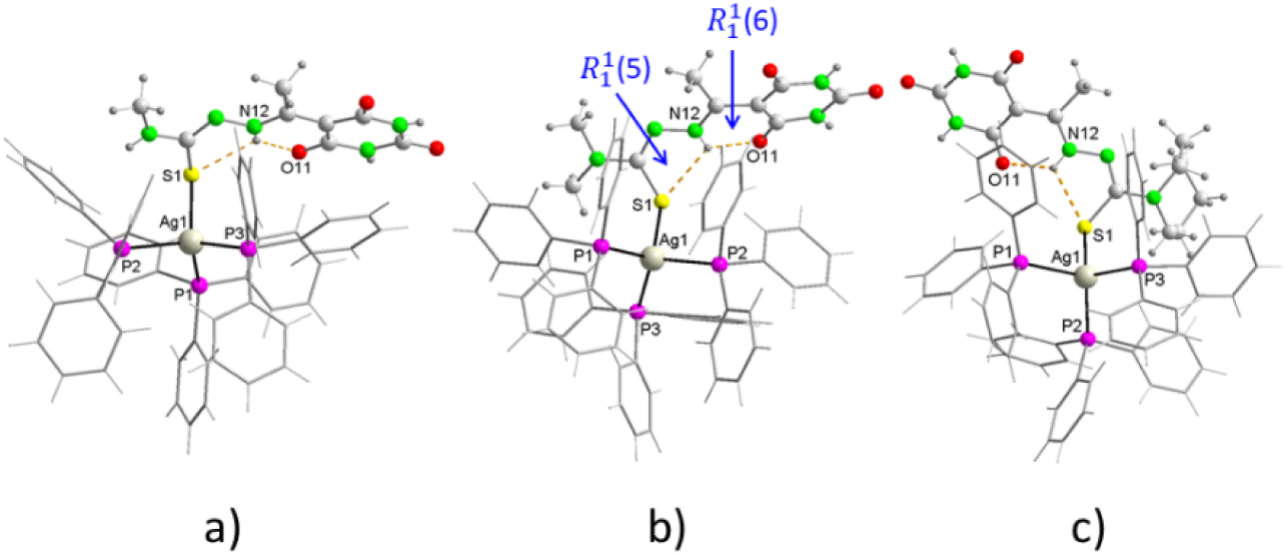
Molecular structure of (a) [Ag­(PPh_3_)_3_(HAcb4NHM)], (b) [Ag­(PPh_3_)_3_(HAcb4NDM)] and (c) [Ag­(PPh_3_)_3_(HAcb4Hexim)].

The Ag–P distances, between 2.5067(12) and 2.5483(12) Å, are lower than the average of 2.558(4) and those of Ag–S, between 2.6123(11) and 2.6136(12) Å, are higher than the average of 2.591(2) Å found in the Cambridge Structural Database (CSD) for 45 complexes with the AgSP_3_ core. The exception is complex **9**, whose values are 2.566(3) and 2.648(2) Å for Ag–P and Ag–S respectively, probably due to some steric hindrance of the hexamethylenimine ring on the 4N atom. The distortion of the AgSP_3_ core can be better understood by considering the P–Ag–P and P–Ag–S bond angles with values between 108.75(3)° and 116.25(3)° respectively, close to the ideal tetrahedral angle of 109.5°. In any case, as in most complexes with the AgSP_3_ core, all P–Ag–P angles are greater than 109.5° and two P–Ag–S angles are smaller and the third greater than 109.5° (Table 3).

**Table 3:** Selected Bond Lengths and Angles for Cited Compounds

[Ag(PPh_3_)_3_(HAbc4NHM)]·2EtOH **(4)**		[Ag(PPh_3_)_3_(HAbc4NHM)]·2EtOH **(4a)**	
Distances [Å]			
Ag(1)-P(3)	2.5070(11)	Ag(1)-P(3)	2.5083(8)
Ag(1)-P(1)	2.5362(12)	Ag(1)-P(1)	2.5355(8)
Ag(1)-P(2)	2.5438(11)	Ag(1)-P(2)	2.5407(8)
Ag(1)-S(1)	2.6123(11)	Ag(1)-S(1)	2.6137(8)
Angles [°]			
P(3)-Ag(1)-P(1)	113.86(4)	P(3)-Ag(1)-P(1)	113.91(3)
P(3)-Ag(1)-P(2)	112.25(4)	P(3)-Ag(1)-P(2)	112.23(3)
P(1)-Ag(1)-P(2)	113.24(4)	P(1)-Ag(1)-P(2)	113.23(3)
P(3)-Ag(1)-S(1)	114.94(3)	P(3)-Ag(1)-S(1)	114.97(3)
P(1)-Ag(1)-S(1)	93.29(4)	P(1)-Ag(1)-S(1)	93.26(3)
P(2)-Ag(1)-S(1)	107.84(4)	P(2)-Ag(1)-S(1)	107.81(3)

[Ag(PPh_3_)_3_(HAbc4NDM)]·3EtOH (6)		[Ag(PPh_3_)_3_(HAbc4NDM)]·2DMSO·H_2_O **(7)**		[Ag(PPh_3_)_3_(HAcb4Hexim)]·3EtOH·3H_2_O **(9)**	
Distances [Å]					
Ag(1)-P(1)	2.5067(12)	Ag(1)-P(1)	2.5120(8)	Ag(1)-P(3)	2.542(3)
Ag(1)-P(2)	2.5442(12)	Ag(1)-P(3)	2.5428(9)	Ag(1)-P(1)	2.573(3)
Ag(1)-P(3)	2.5483(12)	Ag(1)-P(2)	2.5472(9)	Ag(1)-P(2)	2.582(3)
Ag(1)-S(1)	2.6336(12)	Ag(1)-S(1)	2.6329(8)	Ag(1)-S(1)	2.648(2)
Angles [°]					
P(1)-Ag(1)-P(2)	111.14(4)	P(1)-Ag(1)-P(3)	116.25(3)	P(3)-Ag(1)-P(1)	113.98(8)
P(1)-Ag(1)-P(3)	113.73(4)	P(1)-Ag(1)-P(2)	108.75(3)	P(3)-Ag(1)-P(2)	115.06(8)
P(2)-Ag(1)-P(3)	115.10(4)	P(3)-Ag(1)-P(2)	114.51(3)	P(1)-Ag(1)-P(2)	112.60(8)
P(1)-Ag(1)-S(1)	113.31(4)	P(1)-Ag(1)-S(1)	115.33(3)	P(3)-Ag(1)-S(1)	113.04(8)
P(2)-Ag(1)-S(1)	107.37(4)	P(3)-Ag(1)-S(1)	96.64(3)	P(1)-Ag(1)-S(1)	97.63(8)
P(3)-Ag(1)-S(1)	95.10(4)	P(2)-Ag(1)-S(1)	104.47(3)	P(2)-Ag(1)-S(1)	102.62(8)

A comparison of the geometric parameters of the coordinated 5-acetylbarbituric thiosemicarbazone (H_2_AcbtscR) ligands with those of the free ligands reveals minor and inconsequential variations in the lengths and bond angles. An exception to this is the carbon–sulfur distances, which are appreciably longer in the complexes. This is due to the transformation of such bonds from an average of 1.677 Å, corresponding to the thione form (C=S), to 1.745 Å of the thiolate form (C–S) of the monodeprotonated thiosemicarbazones (Table 4). The other exception is related to the molecular conformation of the H_2_Acbtsc. In the known structures, H2Acb4NDH is found to be planar within the error margins, whereas in H_2_Acb4NHM and H_2_Acb4Hexim the midplanes of the 2,4,6-pyrimidinetrione ring and the thiosemicarbazone fragment are rotated relative to each other to dihedral angles of 57.6(1) and 46.7(2)°, respectively.^21^ In the complexes, however, these angles range between 8.9 (4) and 35.4(1)°, including the values found in all the structures (Table 4).

**Table 4:** Significant Geometrical Parameters of Free Thiosemicarbazone Ligands and in Complexes

	C=S [Å]		Diedral Angle [°]^a^	
	Complex	TSC free	Complex	TSC free
{[Ag(PPh_3_)_3_(HAcb4NH_2_)]·EtOH·H_2_O}_2_ (**2**)	1.728(10)	1.677(3)	24.3(3)	2.8(1)
	1.733(9)		27.2(2)	
[Ag(PPh_3_)_3_(HAcb4NM)]·2EtOH (**4**, **4a**)	1.735(3)	1.671(3)	18.7(1)	57.6(1)
[Ag(PPh_3_)_3_(HAcb4NDM)]·3EtOH (**6**)	1.741(5)		33.1(1)	
[Ag(PPh_3_)_3_(HAcb4NDM)]·2DMSO·H_2_O (**7**)	1.743(3)		35.4(1)	
[Ag(PPh_3_)(HAcb4Pip)]_3_·4.5H_2_O (**8**)	1.780(9)		8.3(6)	
	1.742(10)		10.4(6)	
	1.755(12)		8.9(4)	
[Ag(PPh_3_)_3_(HAcb4Hexim)]·3EtOH·3H_2_O (**9**)	1.750(9)	1.683(5)	25.0(1)	46.7(1)

a Dihedral angle between the mean planes of the 2,4,6-pyrimidinetrione ring and the thiosemicarbazone fragment.

Crystal packing of these compounds involves two intramolecular hydrogen bonds (Table 5). In these cases, the N–H bond of the azomethine nitrogen atom of the thiosemicarbazone moiety, N12, functions as a double donor toward the nearest carbonyl oxygen. atom of the 2,4,6-pyrimidinetrione ring, O11, and the thiolate sulfur atom, giving rise to synthons of graph set $R_{1}^{1}$ ­(6) and $R_{1}^{1}$ ­(5), respectively (Figure 5). In addition, the N–H of the barbiturate ring forms two strong intermolecular hydrogen bonds N–H···O with the oxygen atom of the second carbonyl of a thiosemicarbazone ligand molecule of a nearest neighboring complex, O13, and the oxygen atom of some water of crystallization molecules (Table 5). Intermolecular hydrogen bonds O–H···O are also present between crystallization molecules and between these and the oxygen atom of the third carbonyl of the barbiturate ring, O15, as well as some weak bonds (C–H···O, C–H···S and C–H···N).^64^ Ultimately, in all compounds, the complex molecules and the crystallization molecules assemble together in an infinite 1D framework (Figure 5) by N–H···O, O–H···O, C–H···N, and C–H···O hydrogen bonds, and in some cases, π-π interactions are also present.^65^ In **5** and **7**, the π–π interactions are intramolecular and are formed between the barbiturate ring and a phenyl ring **5**, or between phenyl rings **7** (Table 6).

**Figure 5:**
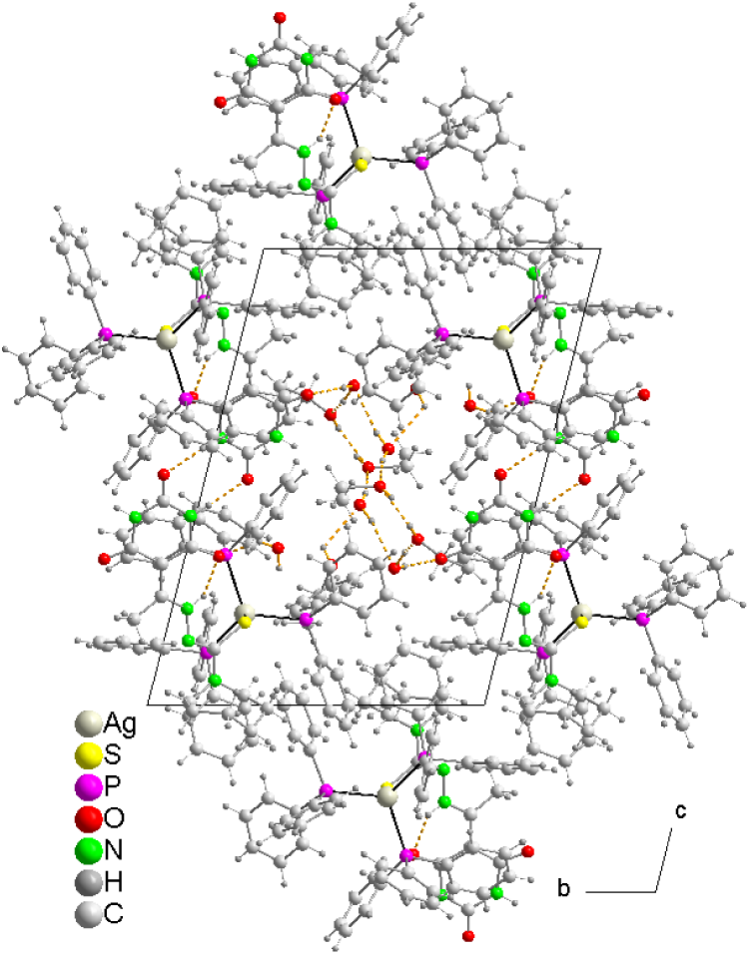
Packing diagram perpendicular to the [011] plane of **8**.

**Table 5:** Hydrogen Bond Parameters [Å, °] for the Compounds Indicated^a^

Compound	D–H···A	D–H	H···A	D···A	∠DHA	Symmetry code
**2**	N(12)–H(12)···S(1)	0.88	2.50	2.920(9)	111.9	
	N(12)–H(12)···O(15)	0.88	1.89	2.599(10)	136.8	
	N(14)-H(14B)···N(23)	0.71	2.45	3.139(10)	164.7	
	N(15)–H(15)···O(23)^a^	0.88	1.95	2.816(12)	168.9	(a) x+1,y+1,z
	N(22)–H(22)···S(2)	0.88	2.52	2.898(8)	107.8	
	N(22)–H(22)···O(25)	0.88	1.89	2.598(9)	135.9	
	N(24)-H(24A)···N(13)	0.76	2.42	3.078(11)	144.2	
	N(25)–H(25)···O(13)^b^	0.88	2.03	2.892(11)	166.8	(b) x-1,y-1,z
**4**	N(11)–H(11)···O(13)^a^	0.83	2.05	2.864(3)	170.0	(a) -x,-y+3,-z
	N(12)–H(12)···S(1)	0.84	2.49	2.927(3)	112.9	
	N(12)–H(12)···O(11)	0.84	1.94	2.613(3)	135.8	
	N(15)–H(15)···O(1)^b^	0.81	2.00	2.807(3)	170.3	(b) -x+1,-y+2,-z
	O(1)–H(1)···O(15)^c^	0.83	1.99	2.780(3)	159.1	(c) x, y-1, z
	O(2)–H(2)···O(11)^e^	0.78	2.10	2.819(3)	152.6	(e) -x, -y + 2, -z + 1
**6**	N(11)–H(11)···O(2)^a^	0.88	1.94	2.807(5)	167.7	(a) -x + 1, -y, -z + 1
	N(12)–H(12)···S(1)	0.88	2.45	2.892(4)	130.0	
	N(12)–H(12)···O(11)	0.88	1.96	2.616(5)	111.0	
	N(15)–H(15)···O(13)^b^	0.88	1.95	2.817(5)	168.2	(b) -x+2,-y,-z+1
	O(1)–H(1)···O(15)^c^	0.84	1.96	2.760(5)	159.3	(c) x-1, y+1, z
	O(2)–H(2)···O(3)^d^	0.84	1.85	2.686(6)	172.2	(d) -x+1, -y+1, -z+1
**7**	N(11)–H(11)···O(3)^a^	0.87	1.90	2.775(3)	179.1	(a) -x+1, -y, -z+1
	N(12)–H(12)···S(1)	0.92	2.49	2.927(3)	113.1	
	N(12)–H(12)···O(11)	0.92	2.00	2.660(3)	127.1	
	N(15)–H(15)···O(13)^b^	0.95	1.93	2.878(3)	173.0	(b) -x+1,-y,-z
	O(3)-H(30A)···O(1)^d^	0.85	1.94	2.780(3)	172.3	(d) -x,-y+1, -z+1
	O(3)-H(30B)···S(10)^e^	0.80	2.88	3.563(2)	144.0	(e) x,y-1, z+1
	O(3)-H(30B)···O(1)^e^	0.80	2.06	2.854(3)	173.0	(e) x,y-1, z+1
**8**	N(11)–H(11)···O(21)	0.88	2.04	2.862(9)	154.5	
	N(12)–H(12)···S(1)	0.88	2.48	2.935(7)	112.8	
	N(12)–H(12)···O(11)	0.88	1.85	2.561(9)	137.0	
	N(15)–H(15)···O(25)^a^	0.88	1.89	2.766(9)	172.3	(a) -x+2, y+1/2, -z+1/2
	N(21)–H(21)···O(13)	0.88	2.05	2.898(10)	161.1	
	N(22)–H(22)···S(2)	0.88	2.43	2.888(7)	112.7	
	N(22)–H(22)···O(21)	0.88	1.94	2.622(9)	133.0	
	N(25)–H(25)···O(15)^b^	0.88	2.05	2.917(9)	169.4	(b) -x+2, y-1/2, -z+1/2
	N(31)–H(31)···O(1)^c^	0.88	1.96	2.837(15)	172.8	(c) -x+1, y+1/2, -z+1/2
	N(32)–H(32)···S(3)	0.88	2.37	2.847(12)	114.3	
	N(32)–H(32)···O(31)	0.88	1.90	2.589(14)	134.2	
	N(35)–H(35)···O(33)^d^	0.88	2.00	2.874(16)	169.0	(d) -x+1, -y+1, -z+1
**9**	N(11)–H(11) ···O(13)^a^	0.88	1.95	2.826(10)	171.4	(a) -x+1, -y+2, -z+1
	N(12)–H(12)···S(1)	0.88	2.43	2.894(9)	114.8	
	N(12)–H(12)···O(11)	0.88	2.02	2.645(10)	127.0	
	O(1)–H(1)···O(3)	0.84	2.19	2.965(11)	153.4	
	O(2)–H(2)···O(6)^b^	0.84	2.34	2.797(15)	114.5	(b) -x+1, -y+1, -z+1
	O(4)-H(40B)···O(11)	0.83	2.15	2.833(13)	139.4	
	O(4)-H(40B)···O(13)^a^	0.83	2.59	3.081(13)	118.5	(a) -x+1, -y+2, -z+1
	O(5)-H(50A)···O(15)^c^	0.92	1.91	2.778(15)	155.7	(c) x,y-1,z
	O(5)-H(50B)···O(1)	0.96	1.84	2.718(16)	151.1	
	O(6)-H(60A)···O(5)^b^	0.91	2.47	3.367(19)	169.4	(b) -x+1, -y+1, -z+1
	O(6)-H(60B)···O(3)	0.89	1.79	2.673(16)	168.8	

a The letters in brackets refer to the symmetry codes shown in the text and figures.

**Table 6:** Intra- and Intermolecular π···π Interaction Parameters (Å, °)^a^

Compound	π···π	Cg(I)···Cg(J)	α	CgI–Perp/CgJ–Perp	Slippage
**4**	Cg(1)···Cg(8)	3.653(2)	15.3(2)	3.249(1)/3.568(2)	0.786
	Phenyl rings defined by atoms C(1): N11/C11/C16/C15/N15/C13; C(8): C57/C58/C59/C60/C61/C62				
**7**	Cg(2)···Cg(6)	3.891(2)	15.8(2)	3.31(1)/3.723(1)	1.131
	Phenyl rings defined by atoms C(2): C21/C22/C23/C24/C25/C26; C(6): C45/C46/C47/C48/C49/C50				
**8**	Cg(4)···Cg(13)	3.8270(9)	23	3.2445/3.7631	
	Cg(6)···Cg(14)	3.8538(9)	9	3.1211/3.4340	1.749
	Cg(12)···Cg(12)^f^	3.8620(9)	0	3.3939/3.3939	1.843
	Phenyl rings defined by atoms C(4): N21/C21/C26/C25/N25/C23; C(6): N31/C31/C36/C35/N35/C33; C(12): C65/C66/C67/C68/C69/C70; C(13): C71/C72/C73/C74/C75/C76; C(14): C77/C78/C79/C80/C81/C82. Symmetry code, f: -x+2,-y+1,-z.				

a Cg­(I)···Cg­(J): centroid–centroid distance; α: dihedral angle between planes I and J; CgI–Perp/CgJ–Perp: perpendicular distances between the planes.

#### 3.4.3 Structure of {[Ag­(PPh_3_)_3_(HAcb4NH_2_)]·EtOH·H_2_O}_2_ (**2**)

In essence, this compound’s structure is analogous to those previously discussed, with the notable distinction that thiosemicarbazone is 4N unsubstituted, implying the presence of two additional N–H bonds capable of functioning as potential hydrogen bond donors. In this case, the asymmetric unit consists of two symmetrically independent complex molecules linked by two N14–H···N23 (N14···N23, 2.920 Å) and N24–H···N13 (N24···N13, 3.078 Å) These bonds (Table 5) form a homosynthon of graph set $R_{2}^{2}$ ­(8) (Figure 6), so, in a sense, it can be considered a dinuclear species with an Ag···Ag distance of 10.589(4)­Å. In the coordination environment of Ag^+^ ions, the Ag–P distances (av. 2.552 Å) are somewhat longer than those found in the mononuclear complexes (av. 2.538 Å), while the Ag–S distances (av. 2.612 Å) are slightly shorter (2.628 Å) (Table 7). In contrast, when we compare the geometrical parameters of both coordinated thiosemicarbazones with those of the mononuclear complexes (Table 4), it is evident that the S=C distances are the shortest. This indicates that the partial double bond character has decreased less with respect to the free ligand, and that the planarity of the ligands is higher (Table 4).

**Figure 6:**
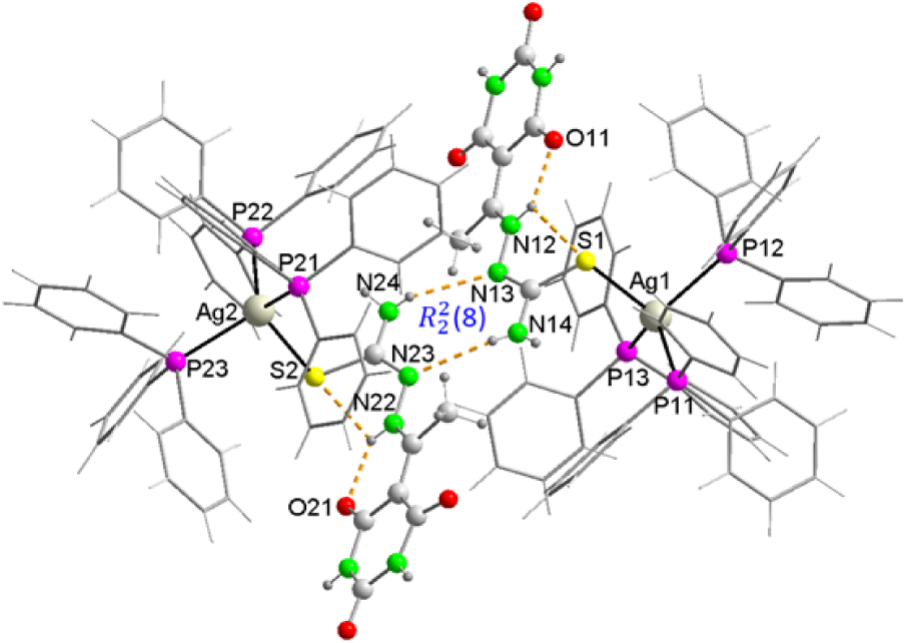
Molecular structure of [Ag­(PPh_3_)_3_(HAcb4NDH)]_2_, showing the intra- and intermolecular hydrogen bonding and the homosynthon of the graph set $R_{2}^{2}$ ­(8).

**Table 7:** Selected Bond Lengths and Angles for Cited Compounds

[Ag(PPh_3_)_3_(HAbc4NH_2_)]·EtOH·H_2_O (**2**)		[Ag(PPh_3_)(HAcb4Pip)]_3_·4.5H_2_O (**8**)	
Distances [Å]			
Ag(1)-P(13)	2.533(3)	Ag(1)-P(1)	2.387(3)
Ag(1)-P(12)	2.540(3)	Ag(1)-S(3)	2.467(3)
Ag(1)-P(11)	2.547(2)	Ag(1)-S(1)	2.474(2)
Ag(1)-S(1)	2.615(3)	Ag(2)-P(2)	2.413(2)
Ag(2)-P(21)	2.568(2)	Ag(2)-S(2)	2.516(2)
Ag(2)-P(22)	2.572(2)	Ag(2)-S(1)	2.566(2)
Ag(2)-S(2)	2.609(2)	Ag(2)-O(11)	2.710(6)
Ag(2)-P(23)	2.609(2)	Ag(3)-P(3)	2.398(2)
Ag(1)···Ag(2)	10.598(4)	Ag(3)-S(3)	2.508(3)
		Ag(3)-S(2)	2.541(2)
		S(1)-S(3)	3.763(4)
		S(1)-S(2)	4.131(3)
		S(2)-S(3)	3.841(4)
		Ag(1)-Ag(2)	4.1503(11)
		Ag(1)-Ag(3)	4.3192(12)
		Ag(2)-Ag(3)	4.6159(12)
Angles [°]			
P(13)-Ag(1)-P(12)		P(1)-Ag(1)-S(3)	129.88(14)
P(13)-Ag(1)-P(11)		P(1)-Ag(1)-S(1)	130.67(9)
P(12)-Ag(1)-P(11)		S(3)-Ag(1)-S(1)	99.23(13)
P(13)-Ag(1)-S(1)		P(2)-Ag(2)-S(2)	123.91(8)
P(12)-Ag(1)-S(1)		P(2)-Ag(2)-S(1)	125.98(8)
P(11)-Ag(1)-S(1)		S(2)-Ag(2)-S(1)	108.74(7)
P(21)-Ag(2)-P(22)		P(2)-Ag(2)-O(11)	113.40(14)
P(21)-Ag(2)-S(2)		S(2)-Ag(2)-O(11)	80.86(13)
P(22)-Ag(2)-S(2)		S(1)-Ag(2)-O(11)	84.13(14)
P(21)-Ag(2)-P(23)		P(3)-Ag(3)-S(3)	123.63(9)
P(22)-Ag(2)-P(23)		P(3)-Ag(3)-S(2)	131.19(8)
S(2)-Ag(2)-P(23)		S(3)-Ag(3)-S(2)	99.06(10)
		Ag(1)-S(1)-Ag(2)	110.86(9)
		Ag(2)-S(2)-Ag(3)	131.78(9)
		Ag(1)-S(3)-Ag(3)	120.51(13)

In the crystal structure, the dimer is linked to two nearest neighbors through a supramolecular heterosynthon graph set $R_{2}^{2}$ ­(8) (Figure 7) formed between the para carbonyl of the 2,4,6-pyrimidinetrione ring and a neighboring amino group, with distances N15···O23^a^ of 2.82 Å and N25···O13^b^ of 2.89 Å (*a* = 1 + x, 1 + y, z; *b* = −1 + x, −1 + y, z) (Table 5), forming chains in the direction of the bisector of the angle formed between the crystallographic a and b axes, where both supramolecular synthons form robust building blocks in the form of homosynthon – heterosynthon – homosynthon −. In such packing, methanol and water molecules of crystallization cooperate in the formation of a 3D network by forming some weak hydrogen bonds of the type C–H···O.

**Figure 7:**
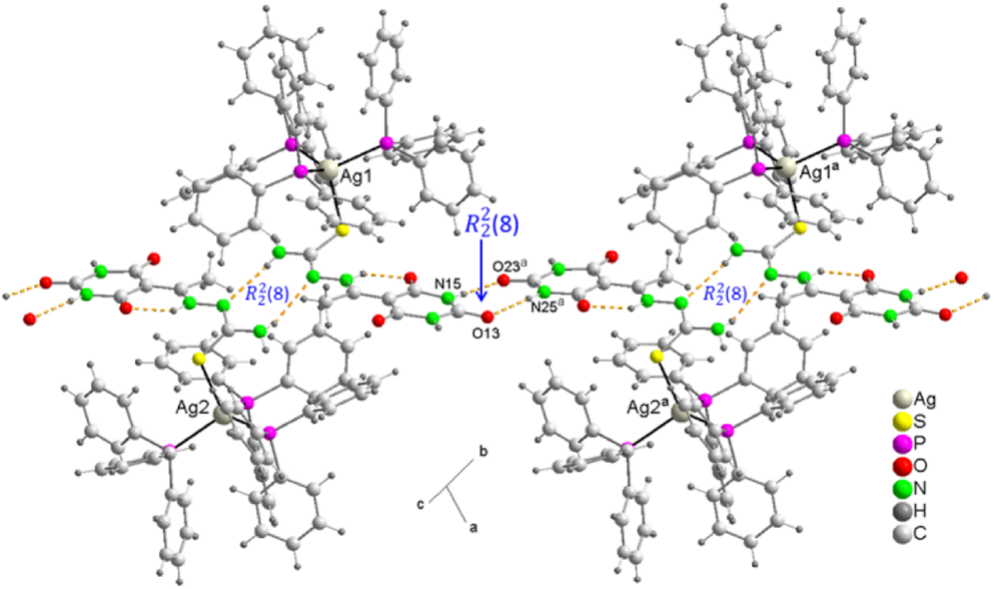
An ab-face projection of a plane formed by hydrogen-bonded molecules in the crystal structure of **2**, showing the supramolecular homo and heterosynthons of the graph set $R_{2}^{2}$ ­(8).

#### 3.4.4 Structure of [Ag­(PPh_3_)­(HAcb4NPip)]_3_·4.5H_2_O (**8**)

Crystallographic analysis indicates that the complex **8** crystallizes in the monoclinic system *P*2_1_/*c* with four and a half water molecules. It is a neutral trinuclear complex of silver and thiolate, where the asymmetric unit, in addition to the water molecules, comprises a complex molecule where the S atoms of three monodeprotonated N-piperidine-(5-acetylbarbituric)­hydrazine-1-carbothioamide ligands bridge three Ag^+^ ions, forming a six-membered Ag_3_S_3_ ring in a twist boat conformation (Figure 8a).

**Figure 8:**
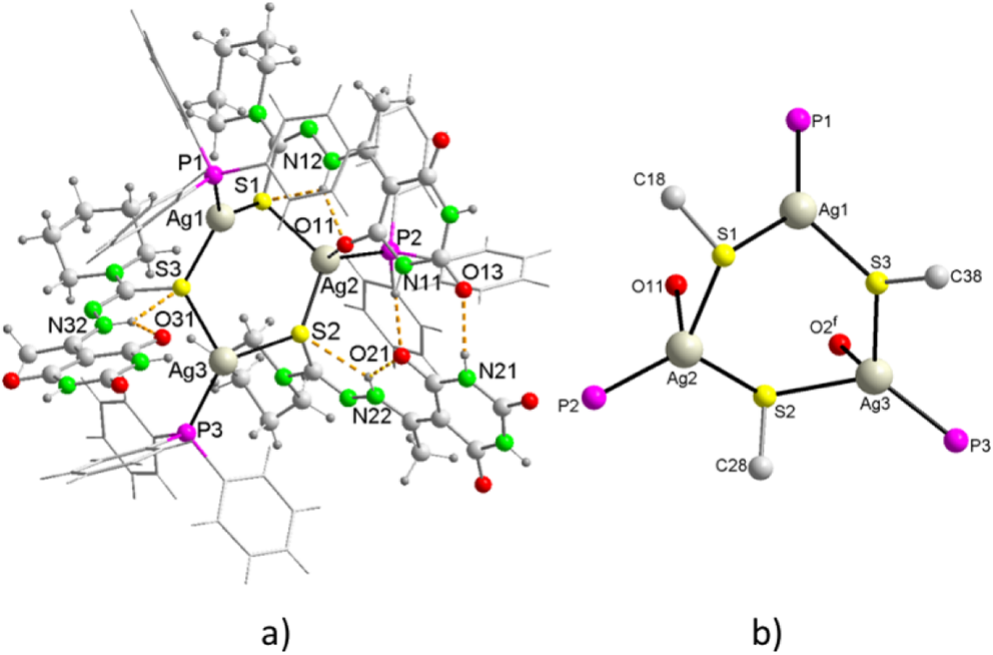
(a) Molecular structure of [Ag­(PPh_3_)­(HAcb4NPip)]_3_ showing the intramolecular hydrogen bonding. Lattice water molecules are omitted for clarity. (b) Trinuclear Ag­(I) cluster core depicting μ-S, terminal O atoms and terminal P atoms.

In this ring, the Ag1 atom is coordinated by two sulfur atoms of two thiosemicarbazones and a P atom of a phosphine, giving rise to a distorted planar trigonal coordination (P–Ag–S, 130.67 and 129.88°; S–Ag–S, 99.23°). Ag2 in the Ag_3_S_3_ ring adopts a distorted tetrahedral geometry defined by a P atom, two μ-S atoms (P–Ag–S, 125.98 and 123.91°; S–Ag–S, 108.73°, P–Ag–O11, 113.39°), and an O atom of a carbonyl in ortho position to the thiosemicarbazone chain in the 2,4,6-pyrimidinetrione ring, at an Ag–O distance of 2.709 Å, which is significantly higher than the average Ag–O distance of 2.446 Å found in the CCDC database. Ag3 exhibits a distorted tetrahedral coordination, analogous to that of Ag2 (P–Ag–S, 131.19 and 123.62°; S–Ag–S, 99.06°, P–Ag–O2, 10 4 0.04 Å), where the oxygen atom O2, corresponding to a crystallization molecule, is at a distance of 3.268 Å from Ag, of the same order of magnitude as the sum of the van der Waals radii of both atoms (Figure 8b). The bond lengths are within the expected ranges, although they are dependent on the coordination mode of the Ag^+^ ions. It is evident that the highest coordination number corresponds to the longest lengths, as observed in the Ag–P bonds, around 2.387(3) Å for planar trigonal coordination, 2.413(2) Å for tetrahedral coordination and 2.398(2) Å for pseudotetrahedral^66^ coordination (Table 7). With regard to Ag–Ag distances, while in certain compounds exhibiting the Ag_3_S_3_ ring it has been observed that they fall within a range close to 3 Å for d^10^–d^10^ attractive interactions, in the compound under study such distances exceed 4 Å, thereby precluding this type of interaction.

In thiosemicarbazones, the bond lengths are also within the expected values, and the S–C distances reflect their thiolate character, exhibiting two hydrogen bonds N–H···S and N–H···O with formation of graph set synthons $R_{1}^{1}$ ­(5) and $R_{1}^{1}$ ­(6). These hydrogen bonds contribute to the planarity of the molecules, as evidenced by the dihedral angle between the mean planes of the 2,4,6-pyrimidinetrione ring and the thiosemicarbazone fragment, which ranges from 8 to 10° (Table 4). Furthermore, thiosemicarbazones that act as thiolate bridges between Ag1–Ag2 and Ag2–Ag3 form an intramolecular homosynthon of graph set $R_{2}^{2}$ ­(8) (Figure 9), which reinforces the pseudotetrahedral coordination of Ag3. Each molecule is bonded to three nearest neighbors by forming supramolecular homosynthons of the amide–amide type of graph set $R_{2}^{2}$ ­(8), giving rise to a 2D network parallel to the ab plane (Figure 9). A similar phenomenon has been observed in [Ag_3_(SMes)_3_(dppb)_2_] (Mes = mesityl; dppb = 1,4-bis­(diphenylphosphino)­butane).^67^ In this case, however, the bridges between Ag_3_S_3_P_3_ subunits are established through the diphosphine ligands. These sheets are kept stacked along the *c* axis. In the 3D network, the layers are associated with each other through hydrogen bonds involving some water crystallization molecules and weak interactions including C–H···N and C–H···O hydrogen bonds as well as π-π stacking interactions of some phenyl rings of PPh_3_ and 2,4,6-pyrimidinetrione rings of thiosemicarbazones (Table 6 and Figure 10).

**Figure 9:**
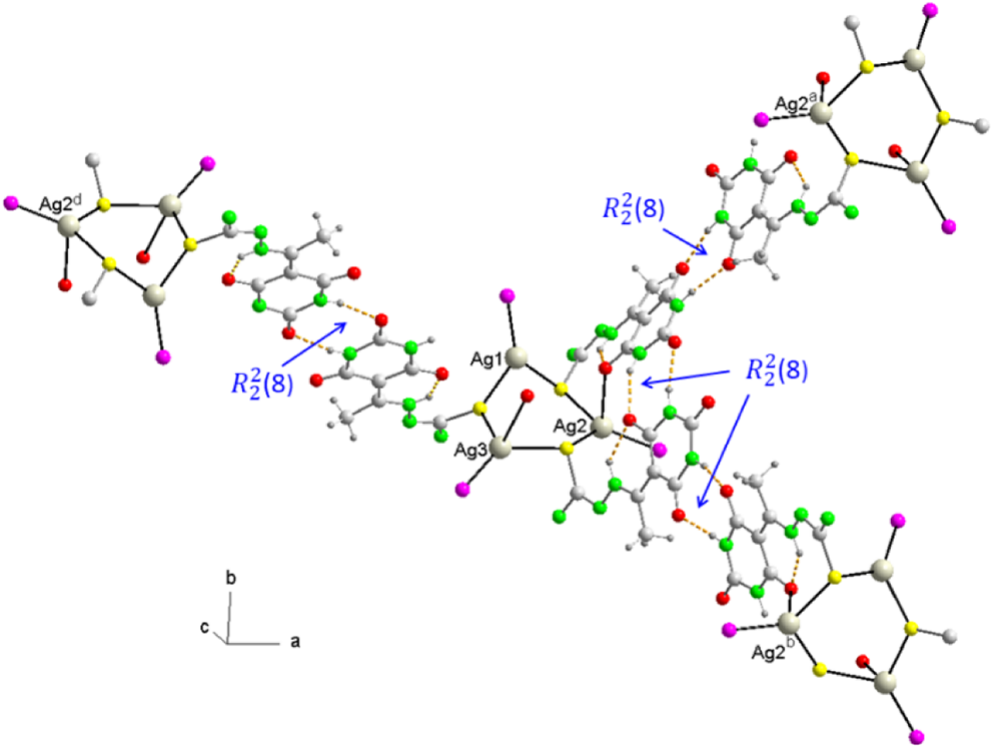
Representation of subunits Ag_3_S_3_P_3_ in a section of 2D layer of **8** showing the supramolecular heterosynthons $R_{2}^{2}$ ­(8). Symmetry codes: a, -x + 2, y + 1/2, -z + 1/2; b, -x + 2, y - 1/2, -z + 1/2; d, -x + 1, -y + 1, -z + 1.

**Figure 10:**
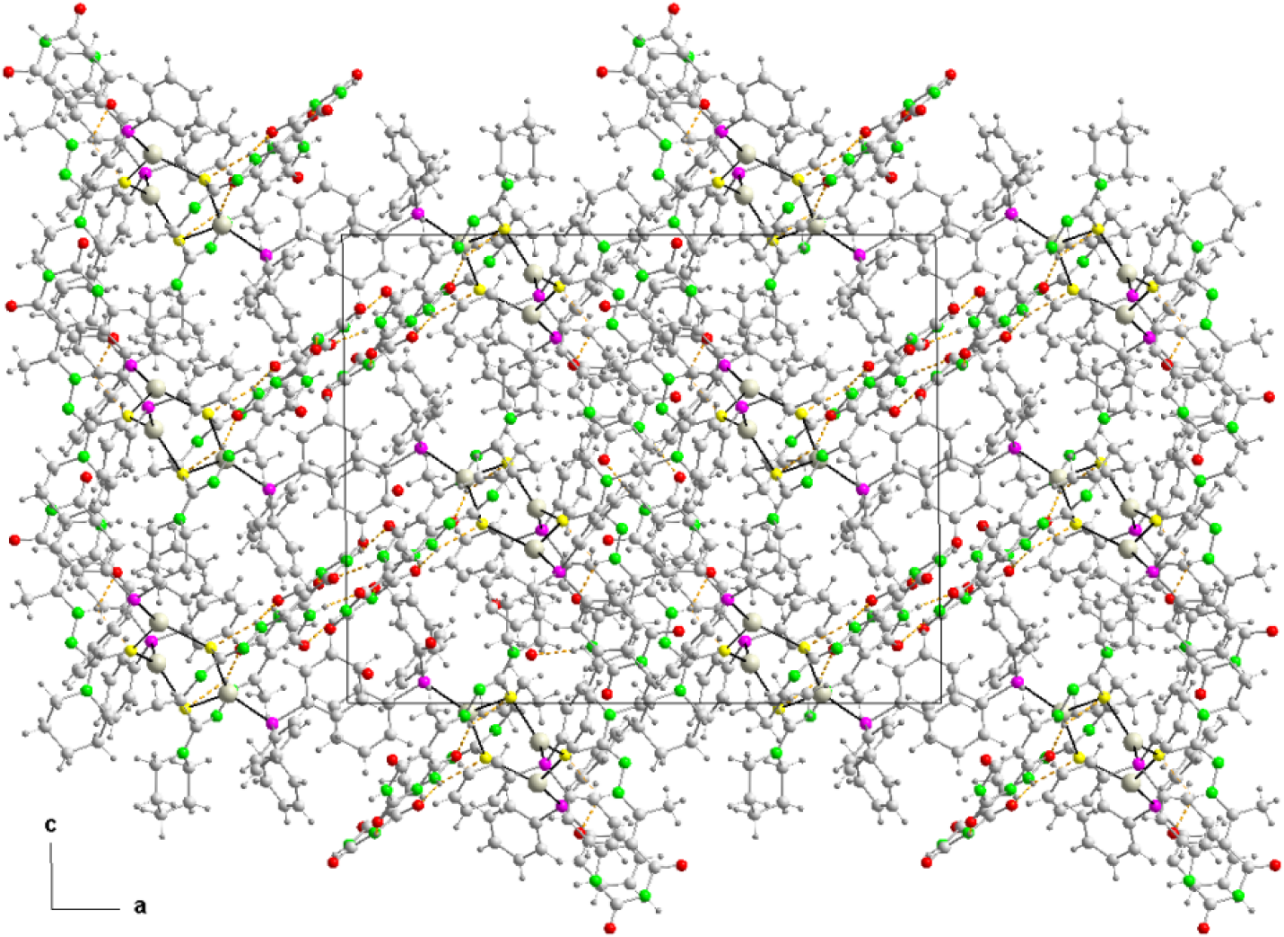
Projection of the 3D network of **8** in the (101) plane.

### 3.5 Theoretical Study

In the previous sections, we have discussed in detail the reactivity of silver­(I) nitrate with acetylbarbituric thiosemicarbazone derivatives, with particular emphasis on the influence of substituents at the nitrogen atom N4. Solid-state analysis of the resulting compounds highlights the crucial role of $R_{2}^{2}$ ­(8) hydrogen-bonding motifs in determining their crystal packing.

The DFT study presented here focuses on the analysis and comparison of these $R_{2}^{2}$ ­(8) motifs across several crystal structures. In particular, for compound **2**, we compare the $R_{2}^{2}$ ­(8) motif formed by the thiosemicarbazide moiety (via NH···N hydrogen bonds) with that formed between barbituric acid rings (via NH···O hydrogen bonds). In compounds **4**, **6**, **7**, and **9**, we examine $R_{2}^{2}$ ­(8) motifs involving different hydrogen-bond donor groups: N1–H (compounds **6** and **9**) and N5–H (compounds **6** and **7**). Atom numbering is provided in Chart I for reference. Additionally, in compound **8**, we compare the intramolecular and intermolecular $R_{2}^{2}$ ­(8) motifs as depicted in Figure 9.

For computational efficiency, reduced models of the original compounds were employed in the theoretical study. For example, a full dimeric assembly of compound **8** contains over 400 atoms. To simplify calculations, triphenylphosphine ligands were replaced with trimethylphosphine, a substitution expected to have minimal impact on the hydrogen-bonding interactions within the $R_{2}^{2}$ ­(8) motifs.

We first computed the molecular electrostatic potential (MEP) surfaces for the mononuclear complexes [Ag­(PMe_3_)_3_(HAcb4NH_2_)], [Ag­(PMe_3_)_3_(HAcb4NHM)], [Ag­(PMe_3_)_3_(HAcb4NDM)], and [Ag­(PPh_3_)_3_(HAcb4Hexim)], used as representative models for compounds **2**, **4**, **6**, **7**, and **9**, respectively. These MEP surfaces, shown in Figure 11, reveal that the oxygen atoms of the barbituric ring possess strongly negative electrostatic potentials, confirming their excellent hydrogen-bond acceptor character. This is consistent with the formal negative charge on the ring following deprotonation, which is predominantly localized on the oxygen atom that forms an intramolecular hydrogen bond with the protonated nitrogen of the thiosemicarbazide group.

**Figure 11:**
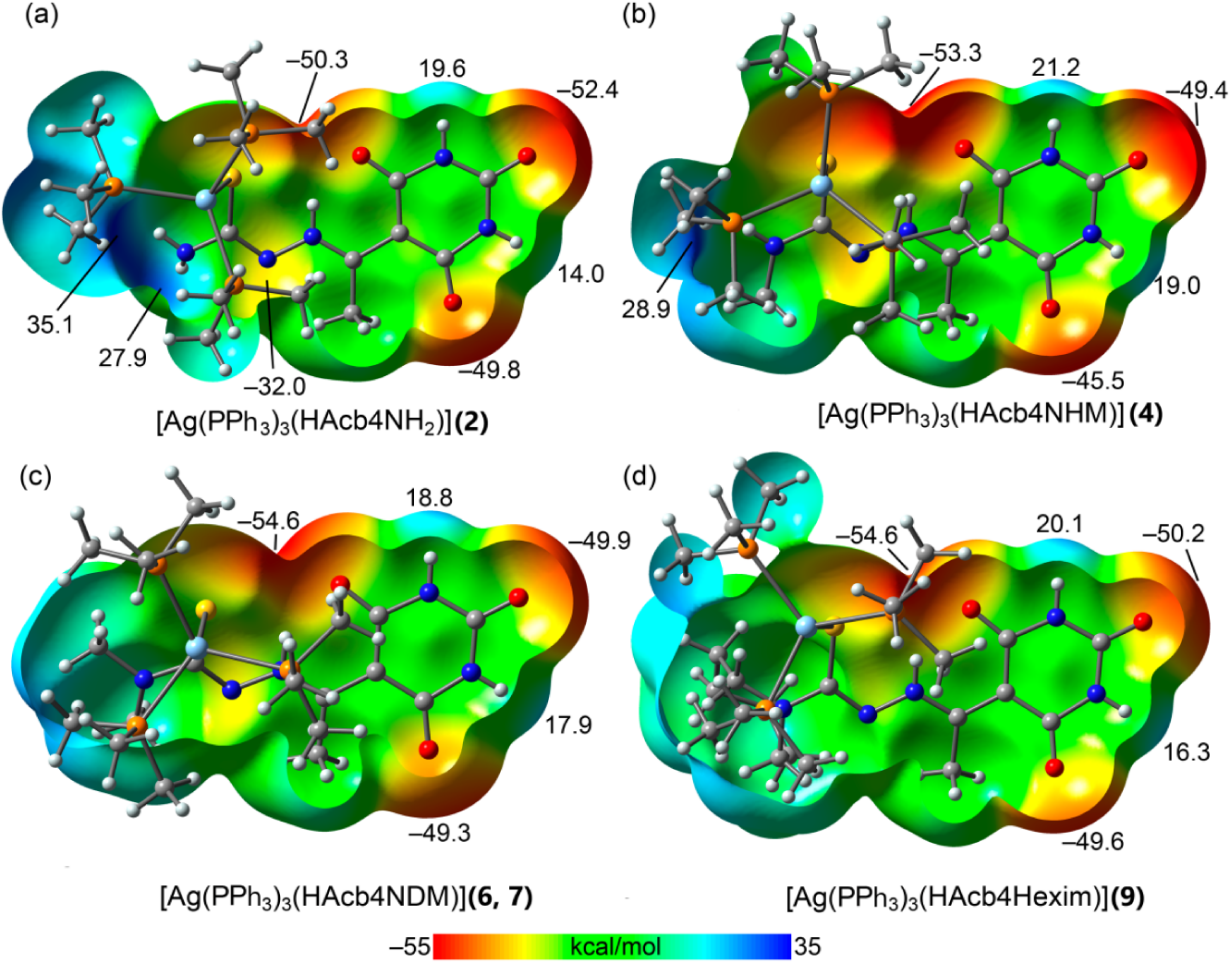
MEP open surfaces of models of [Ag­(PPh_3_)_3_(HAcb4NH_2_)] (a), [Ag­(PPh_3_)_3_(HAcb4NHM)] (b), [Ag­(PPh_3_)_3_(HAcb4NDM)] (c), and [Ag­(PPh_3_)_3_(HAcb4Hexim)] (d) at the PBE0-D3/def2-TZVP level of theory. Values at selected points in kcal/mol. For computational economy phenyl groups were substituted by methyl groups.

In contrast, the oxygen atom positioned near the methyl group is the least electron-rich, with MEP values ranging from −45.5 to −49.8 kcal/mol. In good agreement with these values, this oxygen atom does not participate in the formation of any $R_{2}^{2}$ ­(8) motifs in the mononuclear complexes. The MEP values at the NH groups of the barbituric ring are modest (14.0–21.2 kcal/mol), which can be attributed to the anionic nature of the ring.

For the [Ag­(PMe_3_)_3_(HAcb4NH_2_)] and [Ag­(PMe_3_)_3_(HAcb4NHM)] complexes, models for compounds **2** and **4**, the MEP maximum is located at the amino end of the thiosemicarbazide moiety. Additionally, the MEP at the carbazide nitrogen is also negative (−32.0 kcal/mol; see Figure 11a,b), supporting the formation of $R_{2}^{2}$ ­(8) motifs in compound **2** via two reciprocal NH···N hydrogen bonds. The relatively lower MEP at the nitrogen atom, compared to the oxygen atom of the barbituric ring, also explains the longer H···N distances observed in the NH···N $R_{2}^{2}$ ­(8) motifs versus the NH···O analogues.

Figure 12 presents the MEP surface of the trinuclear complex [Ag­(PMe_3_)_3_(HAcb4Pip)]_3_, used as a model for compound **8**. In this system, one barbituric acid unit is isolated, while the other two are engaged in an intramolecular $R_{2}^{2}$ ­(8) motif. The MEP values of the isolated barbituric acid are generally lower than those observed in the mononuclear complexes, suggesting that the trinuclear

**Figure 12:**
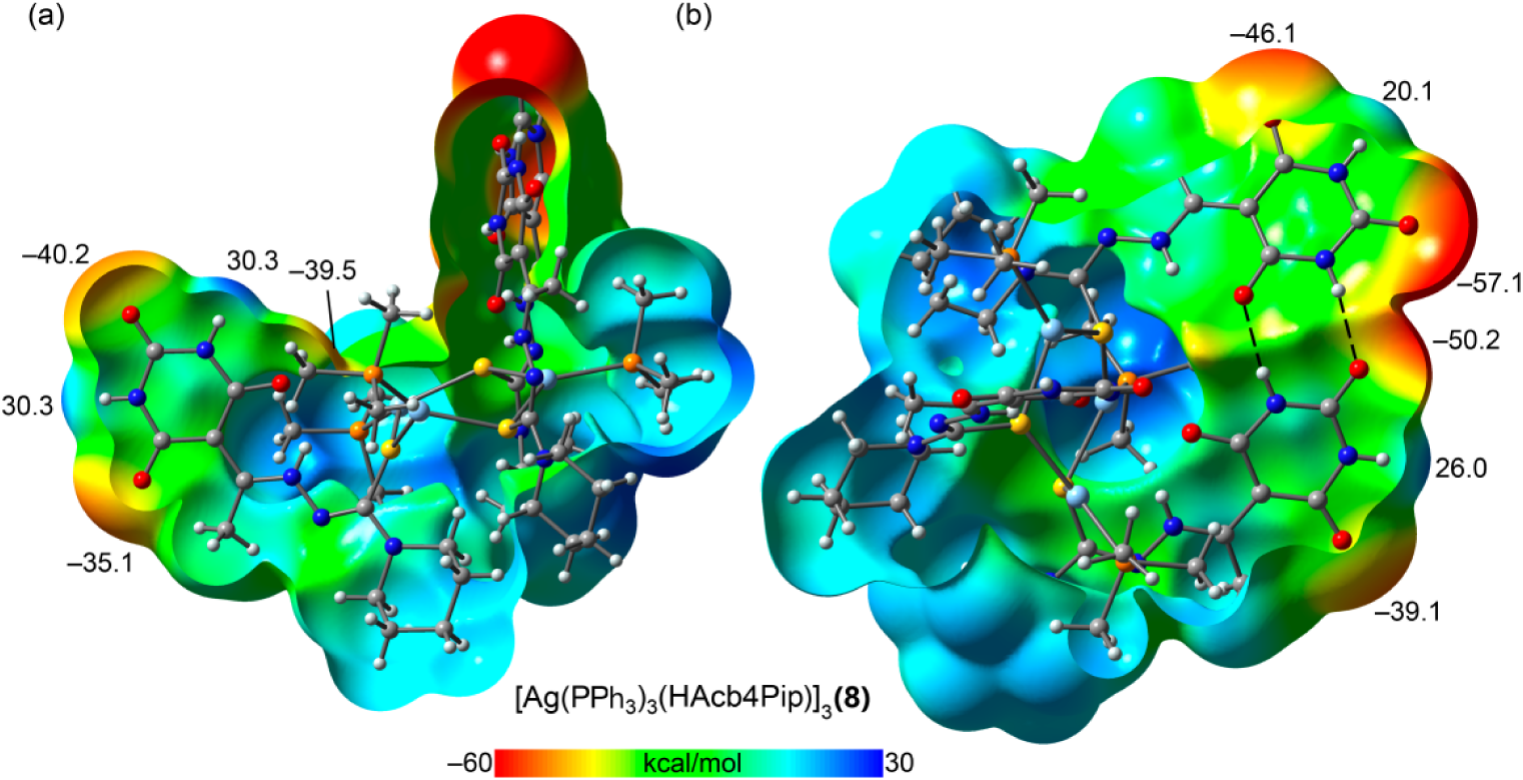
Two views of the MEP open surface of [Ag­(PPh_3_)_3_(HAcb4Pip)]_3_ at the PBE0-D3/def2-TZVP level of theory, showing the isolated barbituric (a) and the intramolecular assembly (b). Values at selected points in kcal/mol. For computational economy phenyl groups were substituted by methyl groups.

In contrast, the barbituric acid units participating in the intramolecular $R_{2}^{2}$ ­(8) motif display enhanced electrostatic potential at key interaction sites, increased H-bond acceptor ability at certain oxygen atoms and greater donor ability at specific NH groups (see Figure 12b). This observation aligns well with the solid-state structure of compound **8** (Figure 9), where the intramolecularly connected barbituric rings further contribute to the hydrogen-bonding network by forming two additional $R_{2}^{2}$ ­(8) motifs, thereby reinforcing the extended supramolecular architecture.

Both $R_{2}^{2}$ ­(8) motifs in the model of compound **2** were analyzed using the Quantum Theory of Atoms in Molecules (QTAIM). As shown in Figure 13, each hydrogen bond is characterized by a bond critical point (BCP, depicted as a small pink sphere) and a bond path (dashed bond) connecting the N–H donor to either O or N acceptor atoms, thereby confirming the formation of the $R_{2}^{2}$ ­(8) motifs (highlighted in blue). Additionally, the analysis reveals the presence of an intramolecular NH···O hydrogen bond, which defines a six-membered supramolecular ring (highlighted in pale green).

**Figure 13:**
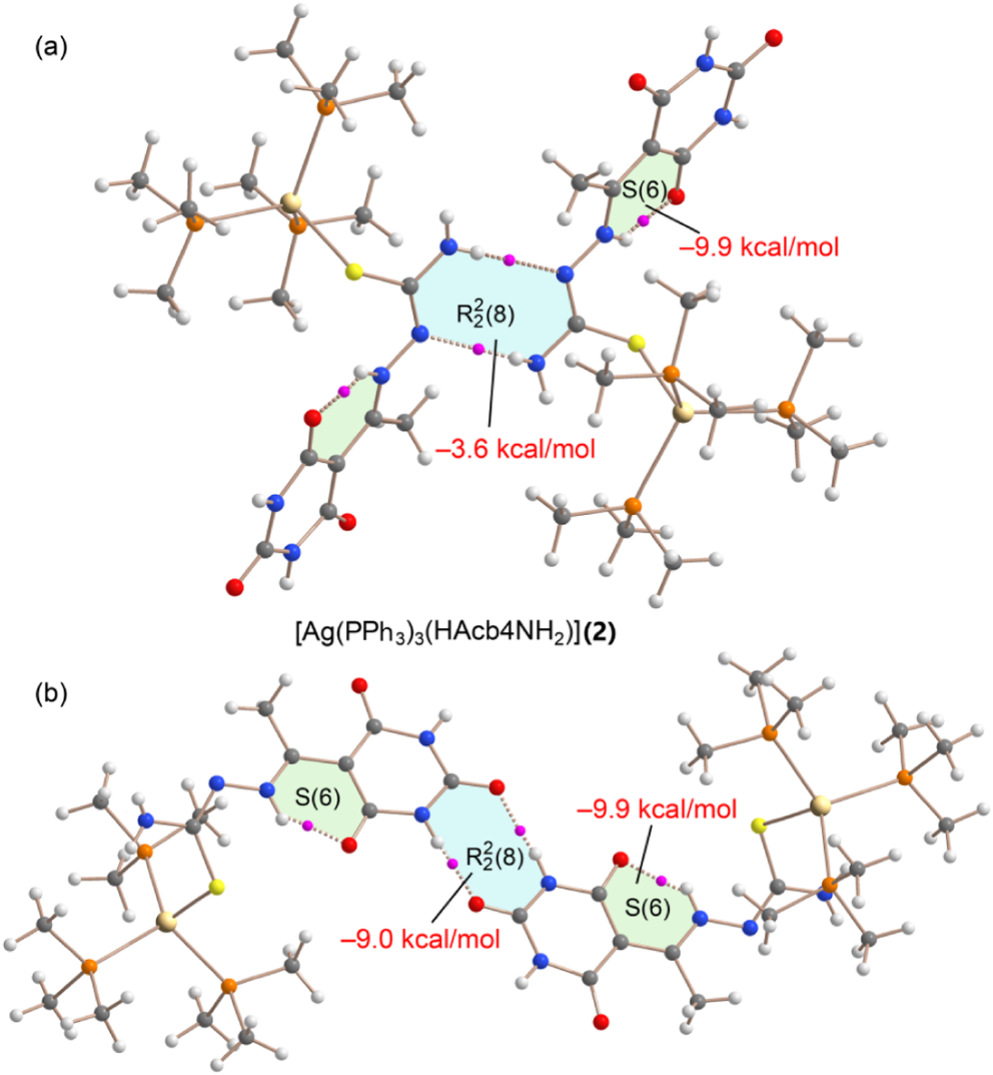
QTAIM analysis of NH···N (a) and NH···O (b) $R_{2}^{2}$ ­(8) motifs of models of compound **2**. The strength of the H-bonds constituting the binding motifs are indicated. BCPs in pink and bond paths as dashed bonds. Only intermolecular interactions are represented apart from the S(6) motifs. For computational economy phenyl groups were substituted by methyl groups.

The interaction energies, derived from QTAIM parameters, are also presented in Figure 13. The intramolecular NH···O hydrogen bond is particularly strong, which can be attributed to the short donor–acceptor distance and the partial cationic and anionic character of the donor and acceptor moieties, respectively. In contrast, the $R_{2}^{2}$ ­(8) motif involving the thiosemicarbazide moieties is significantly weaker (−3.6 kcal/mol) than the corresponding barbituric-based $R_{2}^{2}$ ­(8) motif (−9.0 kcal/mol). These results are in good agreement with the MEP surface analysis discussed in Figure 11, which reflects the relative electrostatic strengths of the involved donor and acceptor groups.

A similar QTAIM analysis was performed for the dimers of compounds **4**, **6**, **7**, and **9**, and the results are presented in Figure 14. The calculated interaction energies for the $R_{2}^{2}$ ­(8) motifs span from −8.8 kcal/mol in compound **9** to −12.1 kcal/mol in compound **7**. Notably, the strongest interactions are observed in compounds **6** and **7**, which are structurally similar and differ only in the identity of the cocrystallized solvent molecule (not included in the computational models).

**Figure 14:**
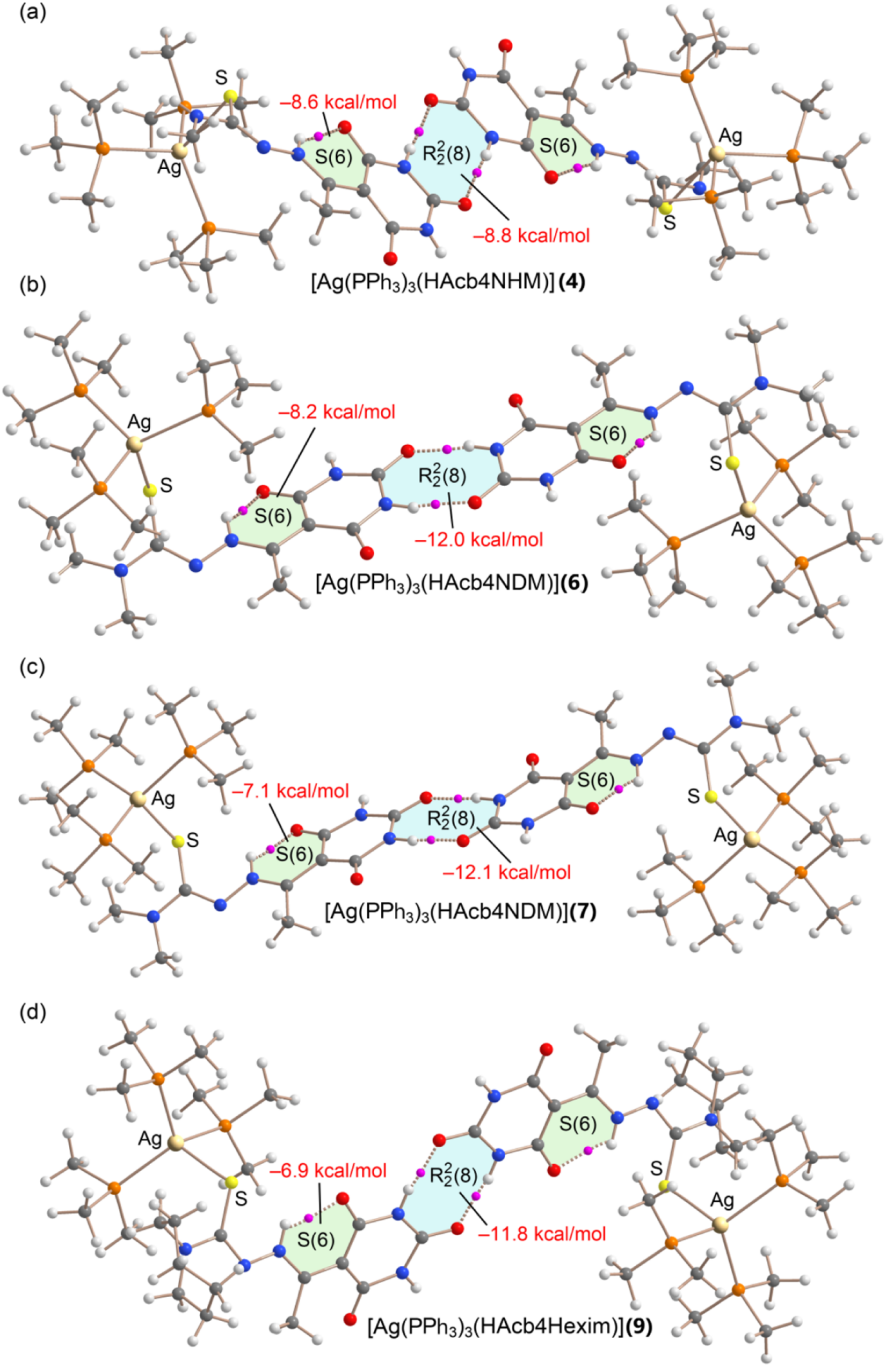
QTAIM analysis of NH···O $R_{2}^{2}$ ­(8) motifs of the dimeric assemblies of models of compounds **4** (a), **6** (b), **7** (c), and **9** (d). The strength of the H-bonds constituting the binding motifs are indicated. BCPs in pink and bond paths as dashed bonds. Only intermolecular interactions are represented apart from the *S*(6) motifs. For computational economy phenyl groups were substituted by methyl groups.

The energetic trends indicate that the stability of the $R_{2}^{2}$ ­(8) motifs is influenced by the nature of the hydrogen-bond donor site. Specifically, motifs involving the N5–H group exhibit stronger interaction energies than those involving N1–H, which could be attributed to differences in local electronic environments and hydrogen-bond geometries. Interestingly, this observation is not fully consistent with the MEP surface analysis, which revealed slightly higher positive electrostatic potentials at the N1–H sites. This discrepancy suggests that the strength of the $R_{2}^{2}$ ­(8) motifs may be more strongly governed by the hydrogen-bond acceptor properties rather than the donor, emphasizing the cooperative nature of the interaction.

Finally, a similar QTAIM analysis was carried out for compound **8** (see Figure 15), focusing on a comparison between the intermolecular and intramolecular $R_{2}^{2}$ ­(8) motifs. As expected, the intermolecular motif is stronger (−9.9 kcal/mol) than the intramolecular one (−8.6 kcal/mol), likely due to geometric constraints that prevent full coplanarity in the intramolecular arrangement. It is particularly informative to compare the intermolecular $R_{2}^{2}$ ­(8) motif in compound **8** with those observed in the mononuclear compounds **6** and **7**, where the same hydrogen-bond donor group (N5–H) is involved. In compound **8**, this interaction is approximately 2 kcal/mol weaker, which aligns well with the MEP surface analysis predicting lower electrostatic potential values at the donor site in the trinuclear system. In contrast, the strength of the intramolecular NH···O hydrogen bond forming the S(6) motif in compound **8** is comparable to that observed in compounds **6** and **7**, despite the structural complexity of the trinuclear system.

**Figure 15:**
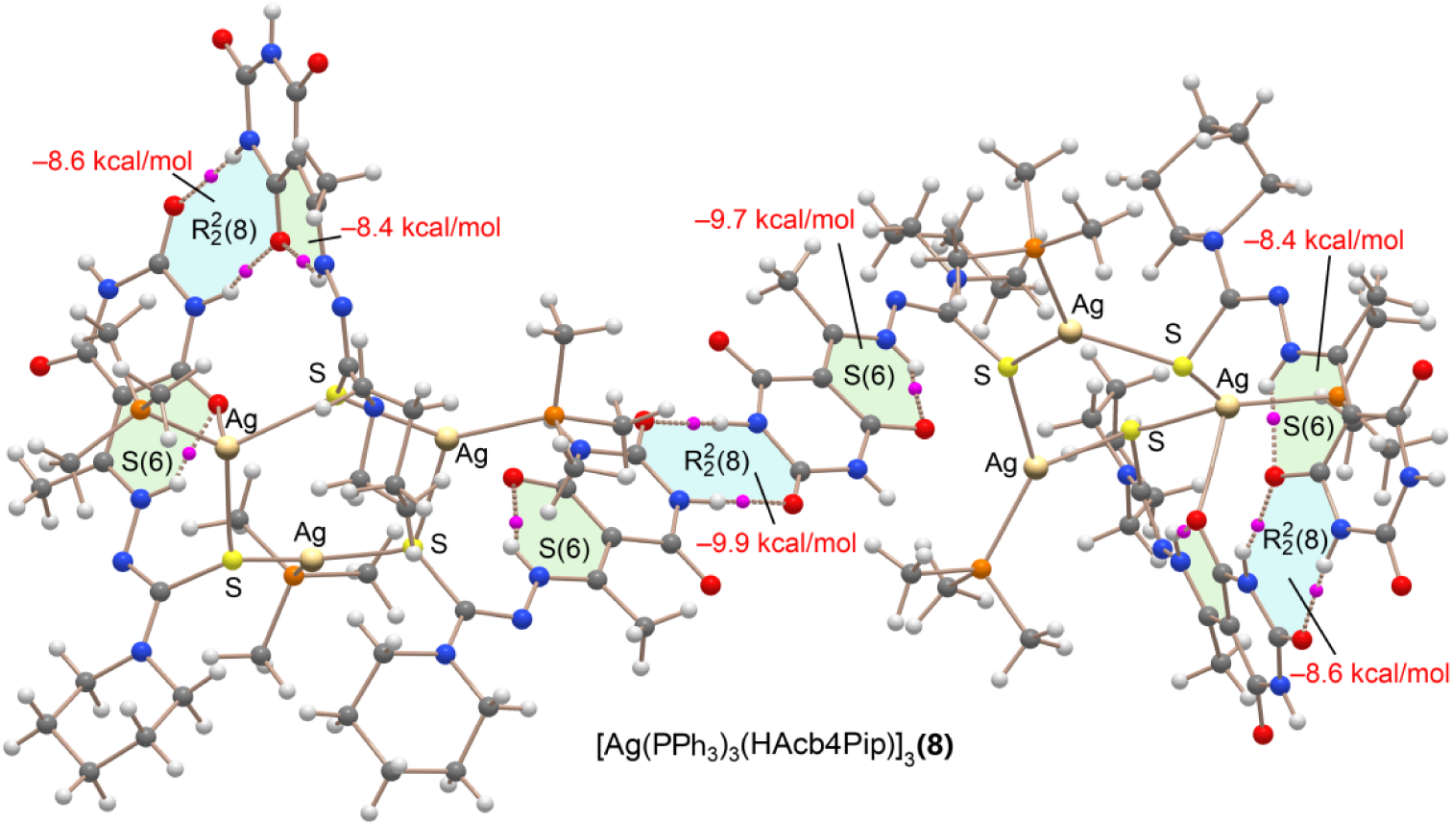
QTAIM analysis of NH···O $R_{2}^{2}$ ­(8) motifs of the dimeric assembly of compound **8**. The strength of the H-bonds constituting the binding motifs are indicated. BCPs in pink and bond paths as dashed bonds. Only intermolecular interactions are represented apart from the $R_{2}^{2}$ ­(8) and S(6) motifs. For computational economy phenyl groups were substituted by methyl groups.

## 4 Conclusions

This research describes the synthesis of nine new silver­(I) complexes formed using five 5-acetylbarbituric acid thiosemicarbazone derivatives as primary ligands and triphenylphosphine as an auxiliary ligand. Polycrystalline samples of complexes were characterized using infrared (IR) spectroscopy, elemental analysis, X-ray crystallography, and mass spectrometry (**4**, **6**, **8**, and **9**). When solubility permitted, nuclear magnetic resonance (NMR) spectroscopy was also employed (compounds **3**, **5**, **6**, **8**, and **9**). The synthesized complexes respond to Ag:PPh_3_:TSC stoichiometries with the following molar ratios: 1:1:1 (**3** and **8**); 1:2:1 (**1**); 1:3:1 (**2**, **4**, **6**, **7**, and **9**); and 1:1:2 (**5**). Two of the complexes are ionic (**3** and **5**), with NO_3_^–^ as the counterion, the rest are molecular. The complexes crystallized with a variable number of H_2_O, ethanol, or DMSO molecules. This study highlights the rich chemical and structural diversity and supramolecular complexity of silver­(I) complexes derived from 5-acetylbarbituric thiosemicarbazones, which are modulated by variations at the N4 position. Experimental analyses reveal various coordination modes, nuclearities. The 1:3:1 ratio complexes clearly show a well-defined steric effect in accommodating the PPh_3_ ligand, as evidenced by a comparison with other similar complexes reported in the literature. The thiosemicarbazones show the expected S-monodentate coordination mode in a monoanionic tiona-keto manner, while the silver­(I) ion has a tetrahedral coordination environment. The trinuclear Ag­(I) cluster exhibits varied coordination geometry around the Ag­(I) centers. The focus of this investigation also lies in the examination of packing architectures where hydrogen bonds, particularly through $R_{2}^{2}$ ­(8) motifs, plays a central role. Complementary DFT and QTAIM calculations provide quantitative insight into the energetics of these motifs. These calculations confirm the dominant role of barbituric oxygen atoms as hydrogen-bond acceptors and elucidate subtle variations in interaction strength due to donor group identity and molecular geometry. Together, these findings offer a comprehensive understanding of how electronic and steric effects influence molecular assembly and noncovalent stabilization in silver­(I) systems, contributing to the rational design of coordination-driven crystal engineering.

## Supplementary Material



## Notes

(PDF) The Supporting Information is available free of charge at https://pubs.acs.org/doi/10.1021/acsomega.5c05539.

ESI–MS (Figures S1 and S2), FT-IR spectra (Figures S3–S6), ^1^H (Figures S7 and S8), ^13^C­{^1^H} (Figures S9 and S10) and ^31^P­{^1^H} (Figures S11–S13) NMR spectra (PDF)
