# Supplementary material for: Reactivity of Acetylbarbituric Thiosemicarbazone Derivatives with Silver(I) Nitrate and the Influence of Substituents at Nitrogen Atom 4N on the Bonding and Nuclearity of the Resulting Silver(I) Complexes
Source: ACS Omega. 2025 Sep 12;10(37):42862–81. doi: 10.1021/acsomega.5c05539 (PMC12461326; doi:10.1021/acsomega.5c05539)
Supplement: Supplementary file 1 [file ao5c05539_si_001.pdf]

Supporting Information

Reactivity of acetylbarbituric thiosemicarbazone derivatives with silver(I) nitrate and the influence of substituents at nitrogen atom 4N on the bonding and nuclearity of the resulting silver(I) complexes.

Alfonso Castiñeiras[1]*, *Nuria Fernández-Hermida*[1], *Antonio Frontera*[2]*, *Isabel García-Santos*[1], *Lourdes Gómez-Rodríguez*[1]

[1] Department of Inorganic Chemistry, Faculty of Pharmacy, University of Santiago de Compostela, 15782 Santiago de Compostela, Spain

[2] Department de Química, Universitat de les Illes Balears, Crta. de Valldemossa km 7.5, 07122 Palma de Mallorca, Spain

Index

Table S1. Selected bond lengths and Angles data for **$\{[Ag(H_2O)_{14}](NO_3)\}_n$, $Ag(PPh_3)_4](NO_3)\cdot EtOH$** and **$AgCl(PPh_3)]_4$** 3

Figures S1. MALDI-TOF mass spectra of **4**, **6**, **8** and **9** 4

Figures S2. MALDI-TOF mass spectrum of **$[Ag(PPh_3)_4](NO_3)\cdot EtOH$** 6

Figures S3. FT-IR spectra of **1** to **6**, **8** and **9** (4000-400 cm^{-1}) 7

Figures S4. FT-IR spectra of **$[Ag(PPh_3)_4](NO_3)\cdot EtOH$** (4000-400 cm^{-1}) 15

Figures S5. FT-IR spectra of **3** to **6**, **8** and **9** (500-100 cm^{-1}) 16

Figure S6. FT-IR spectrum of **$[Ag(PPh_3)_4](NO_3)\cdot EtOH$** (500-100 cm^{-1}) 19

Figures S7. ^{1}H NMR spectra of **1**, **5**, **6**, **8** and **9** 20

Figure S8. ^{1}H NMR spectrum of **$[Ag(PPh_3)_4](NO_3)\cdot EtOH$** 22

Figures S9. ^{13}C NMR spectra of **6**, **8** and **9** 23

Figure S10. ^{13}C NMR spectrum of **$[Ag(PPh_3)_4](NO_3)\cdot EtOH$** 24

Figures S11. ^{31}P NMR spectra of **3**, **5**, **6**, **8** and **9** 25

Figure S12. ^{31}P NMR spectrum of $[Ag(PPh_3)_4](NO_3)\cdot EtOH$ (25°C) 28

Figure S13. ^{31}P NMR spectrum of $[Ag(PPh_3)_4](NO_3)\cdot EtOH$ (−60°C) 28

Table S1. Selected bond lengths and Angles data for cited compounds

**$\{[Ag(H_2O)_{14}](NO_3)\}_n$**		**$[Ag(PPh_3)_4](NO_3)\cdot EtOH$**		**$[AgCl(PPh_3)]_4$**	
Distances [Å]					
Ag(1)-O(3)	2.3513(15)	Ag(1)-P(2)	2.6001(12)	Ag(1)-P(1)	2.3823(11)
Ag(1)-O(2)[a]	2.3936(15)	Ag(1)-P(2)[a]	2.6002(12)	Ag(1)-Cl(2)[a]	2.5281(10)
Ag(1)-O(1)[a]	2.3990(15)	Ag(1)-P(1)[a]	2.6147(11)	Ag(1)-Cl(1)	2.6723(12)
Ag(1)-O(1)	2.4014(15)	Ag(1)-P(1)	2.6148(11)	Ag(1)-Cl(2)	2.7566(11)
Ag(1)-O(4)	2.4209(15)			Ag(2)-P(2)	2.3681(12)
Ag(1)-O(2)	2.4465(15)			Ag(2)-Cl(1)	2.5583(11)
Ag(2)-O(7)[b]	2.3591(15)			Ag(2)-Cl(1)[a]	2.6516(10)
Ag(2)-O(6)	2.3672(15)			Ag(2)-Cl(2)	2.7058(11)
Ag(2)-O(7)	2.4070(15)			Ag(1)-Ag(2)[a]	3.3798(5)
Ag(2)-O(4)	2.4165(15)				
Ag(2)-O(3)[c]	2.4641(16)				
Ag(2)-O(5)	2.4735(15)				
Ag(1)-Ag(1)[a]	3.5221(3)				
Ag(2)-Ag(2)[b]	3.3691(14)				
Ag(1)-Ag(2)	4.3262(10)				
Angles [°]					
O(3)-Ag(1)-O(2)[a]	94.70(5)	P(2)-Ag(1)-P(2)[a]	110.03(5)	P(1)-Ag(1)-Cl(2)[a]	137.68(4)
O(3)-Ag(1)-O(1)[a]	98.37(5)	P(2)-Ag(1)-P(1)[a]	110.07(4)	P(1)-Ag(1)-Cl(1)	116.61(4)
O(2)[a]-Ag(1)-O(1)[a]	86.73(5)	P(2)[a]-Ag(1)-P(1)[a]	108.12(4)	Cl(2)[a]-Ag(1)-Cl(1)	101.22(3)
O(3)-Ag(1)-O(1)	101.45(5)	P(2)-Ag(1)-P(1)	108.12(4)	P(1)-Ag(1)-Cl(2)	109.12(4)
O(2)[a]-Ag(1)-O(1)	163.69(6)	P(2)[a]-Ag(1)-P(1)	110.07(4)	Cl(2)[a]-Ag(1)-Cl(2)	91.00(3)
O(1)[a]-Ag(1)-O(1)	93.16(5)	P(1)[a]-Ag(1)-P(1)	110.45(5)	Cl(1)-Ag(1)-Cl(2)	85.86(3)
O(3)-Ag(1)-O(4)	177.85(6)			P(1)-Ag(1)-Ag(2)[a]	150.73(3)
O(2)[a]-Ag(1)-O(4)	83.16(5)			Cl(2)[a]-Ag(1)-Ag(2)[a]	52.12(3)
O(1)[a]-Ag(1)-O(4)	80.70(5)			Cl(1)-Ag(1)-Ag(2)[a]	50.32(2)
O(1)-Ag(1)-O(4)	90.16(5)			Cl(2)-Ag(1)-Ag(2)[a]	96.69(2)
O(3)-Ag(1)-O(2)	90.16(5)			P(2)-Ag(2)-Cl(1)	139.87(4)
O(2)[a]-Ag(1)-O(2)	92.19(5)			P(2)-Ag(2)-Cl(1)[a]	119.77(4)
O(1)[a]-Ag(1)-O(2)	171.46(6)			Cl(1)-Ag(2)-Cl(1)[a]	88.66(3)
O(1)-Ag(1)-O(2)	85.49(5)			P(2)-Ag(2)-Cl(2)	112.20(4)
O(4)-Ag(1)-O(2)	89.99(5)			Cl(1)-Ag(2)-Cl(2)	89.21(4)
O(7)[b]-Ag(2)-O(6)	91.35(5)			Cl(1)[a]-Ag(2)-Cl(2)	97.25(3)
O(7)[b]-Ag(2)-O(7)	90.04(5)				
O(6)-Ag(2)-O(7)	168.55(6)				
O(7)[b]-Ag(2)-O(4)	86.35(5)				
O(6)-Ag(2)-O(4)	87.16(5)				
O(7)[b]-Ag(2)-O(3)[c]	177.32(6)				
O(6)-Ag(2)-O(3)[c]	90.05(5)				
O(7)-Ag(2)-O(3)[c]	89.03(5)				
O(4)-Ag(2)-O(3)[c]	96.00(5)				
O(7)[b]-Ag(2)-O(5)	93.38(5)				
O(6)-Ag(2)-O(5)	96.00(5)				
O(7)-Ag(2)-O(5)	95.26(5)				
O(4)-Ag(2)-O(5)	176.83(6)				
O(3)[c]-Ag(2)-O(5)	84.21(5)				
Symmetry transformations					
a: x−1/2,y,−z+1/2		a: −x+1,y,−z+1/2		a: −x+1,y,−z+1/2	
b: −x+1,−y,−z					
c: x+1/2,y,−z+1/2					

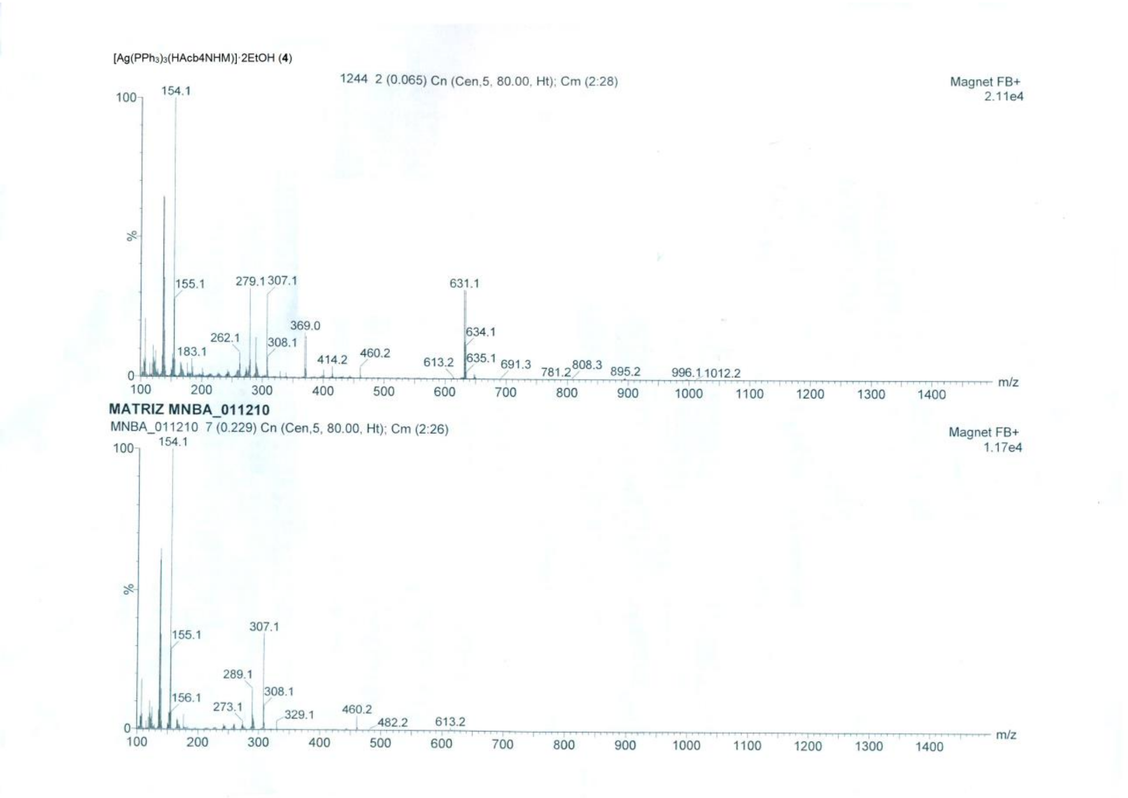

Figures S1.4. MALDI-TOF mass spectra of 4.

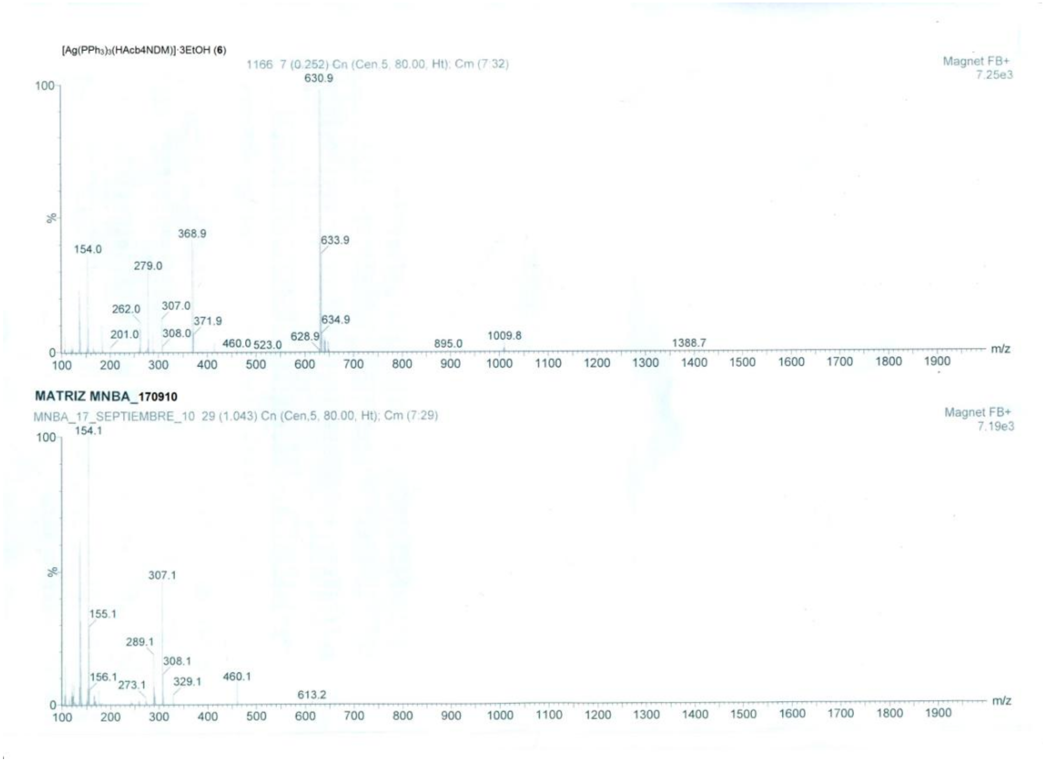

Figures S1.6. MALDI-TOF mass spectra of **6**.

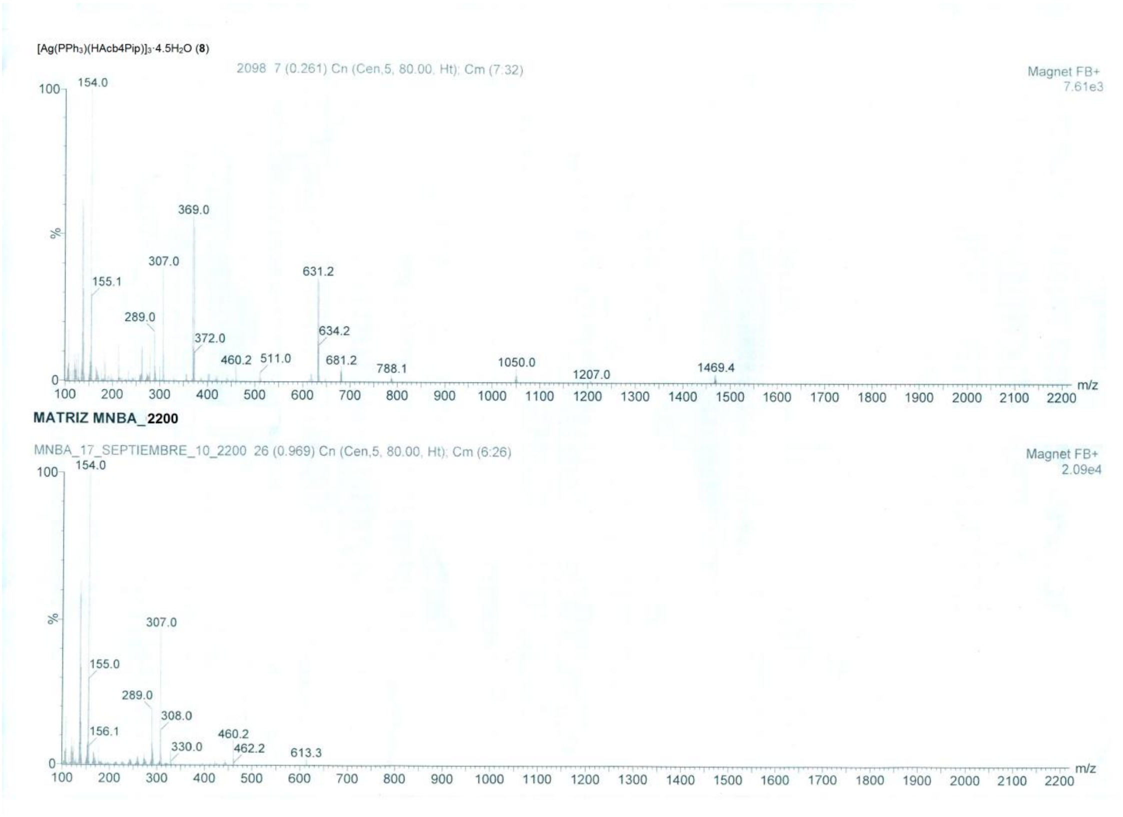

Figures S1.8. MALDI-TOF mass spectra of **8**.

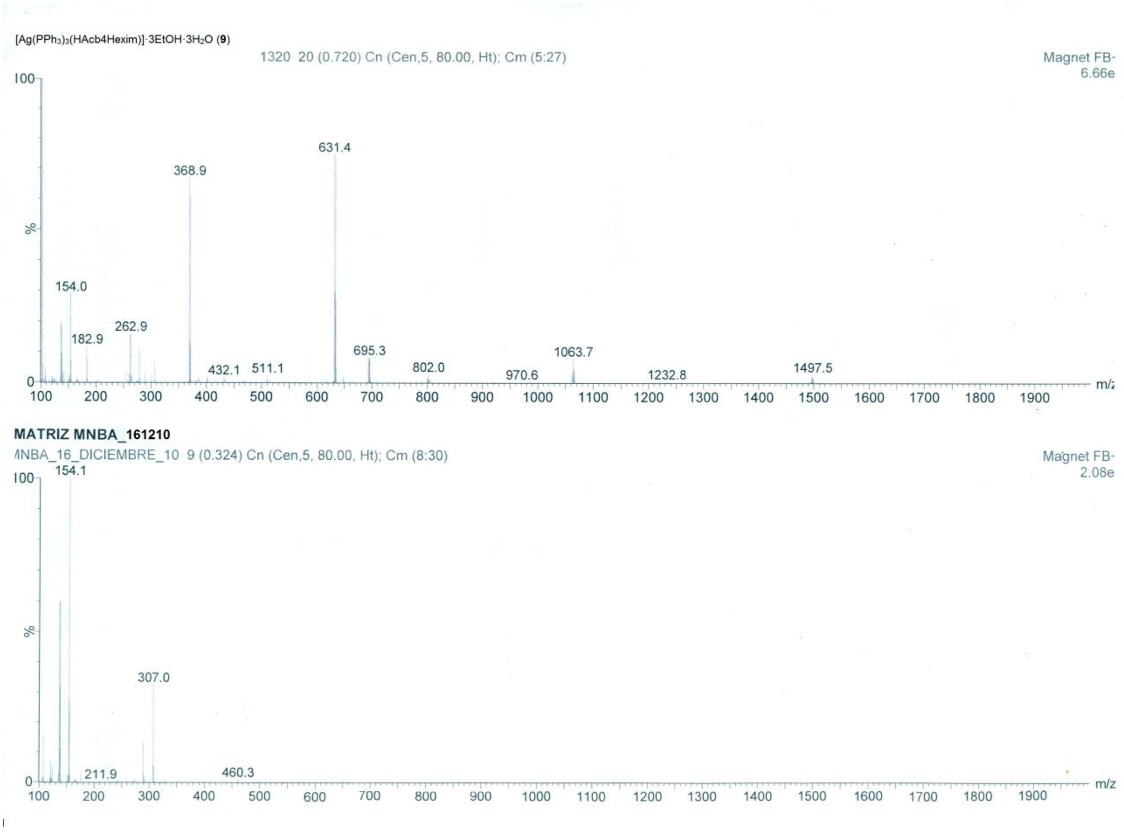

Figures S1.9. MALDI-TOF mass spectra of **9.**

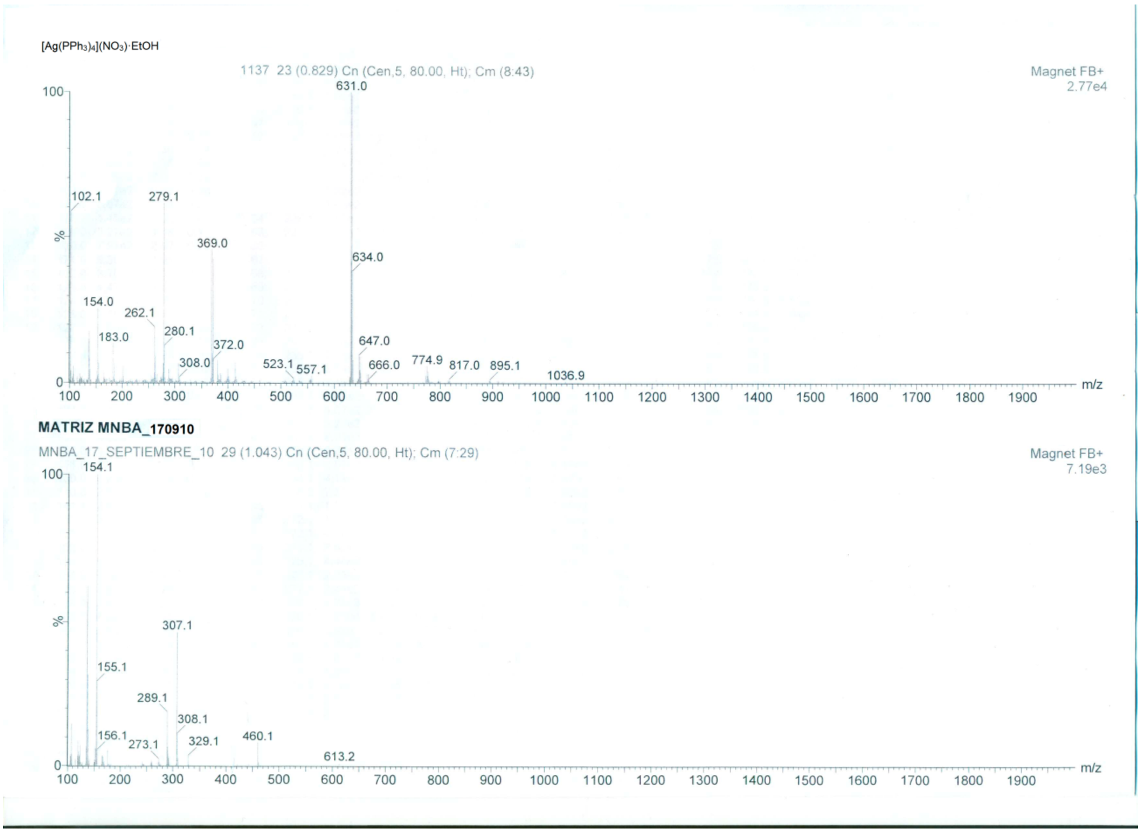

Figures S2. MALDI-TOF mass spectrum of **$[Ag(PPh_3)_4](NO_3)\cdot EtOH$.**

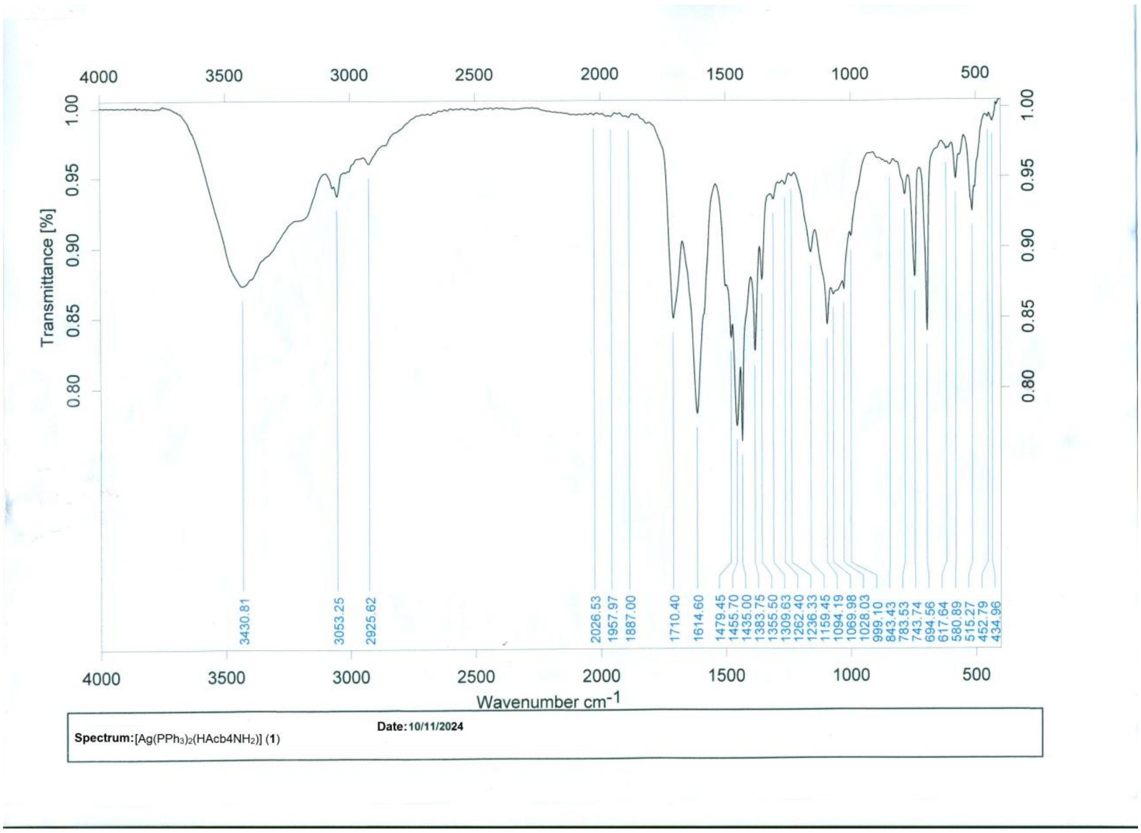

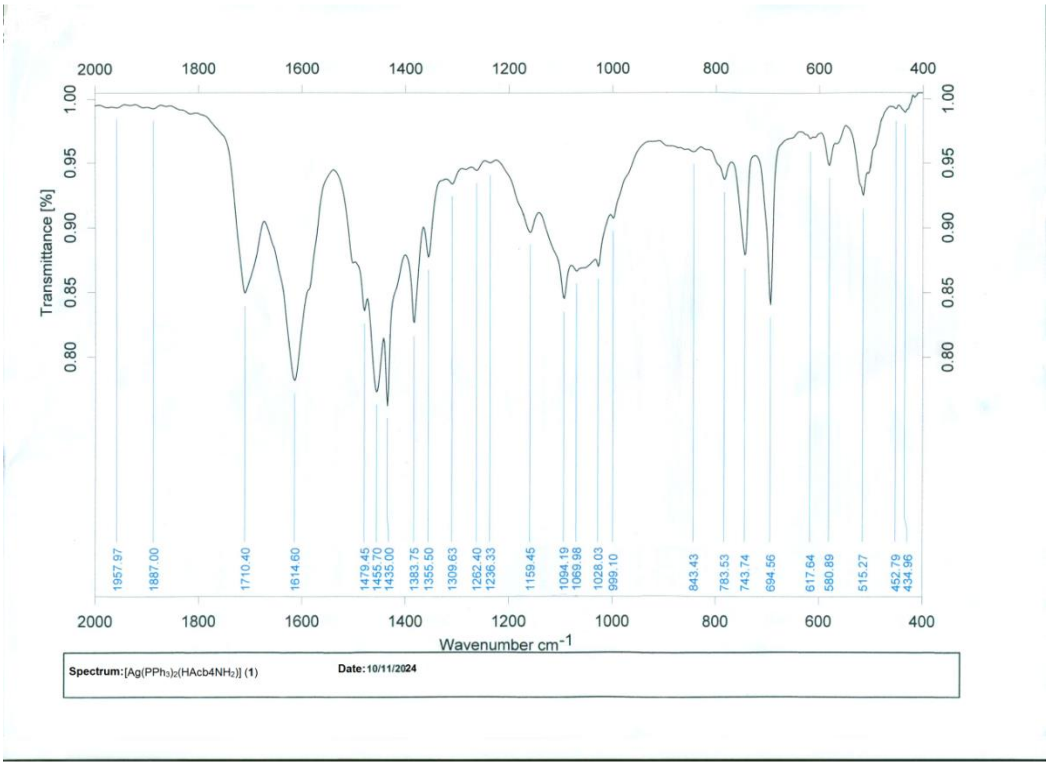

Figures S3.1. FT-IR spectra of **1** (4000-400 cm^{-1})

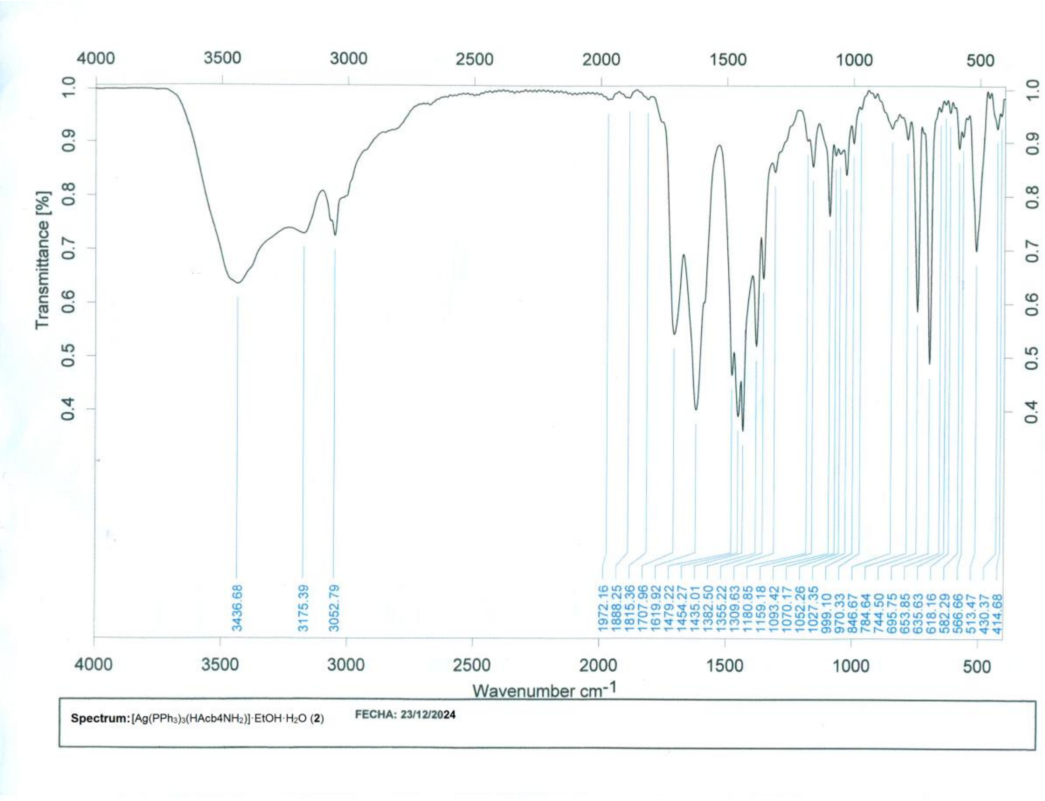

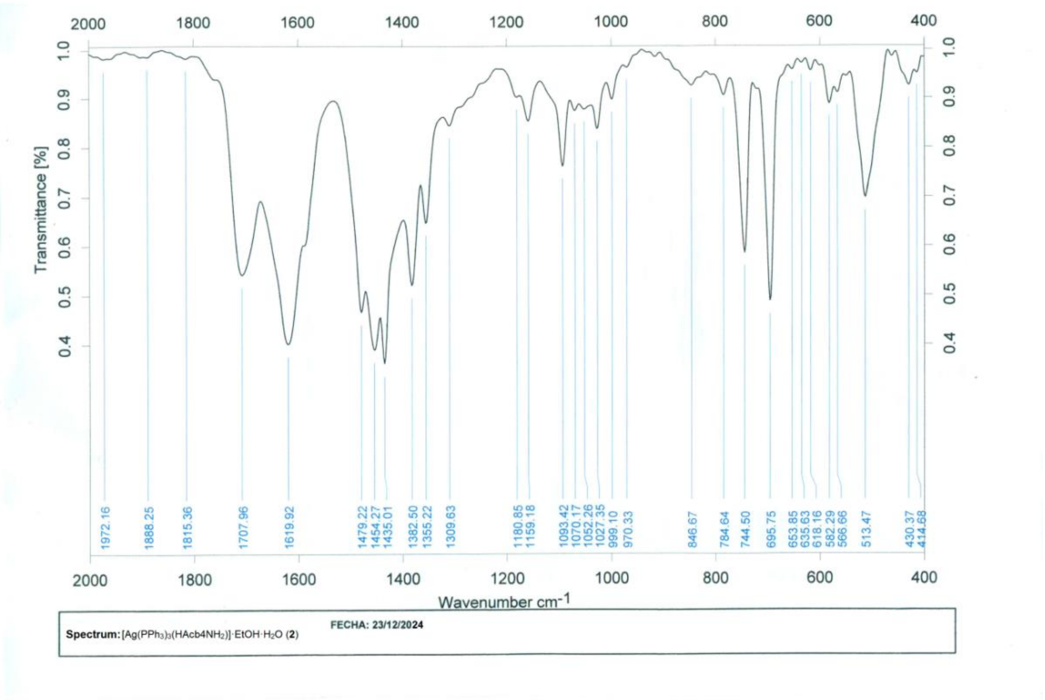

Figures S3.2. FT-IR spectra of **2** (4000-400 cm^{-1})

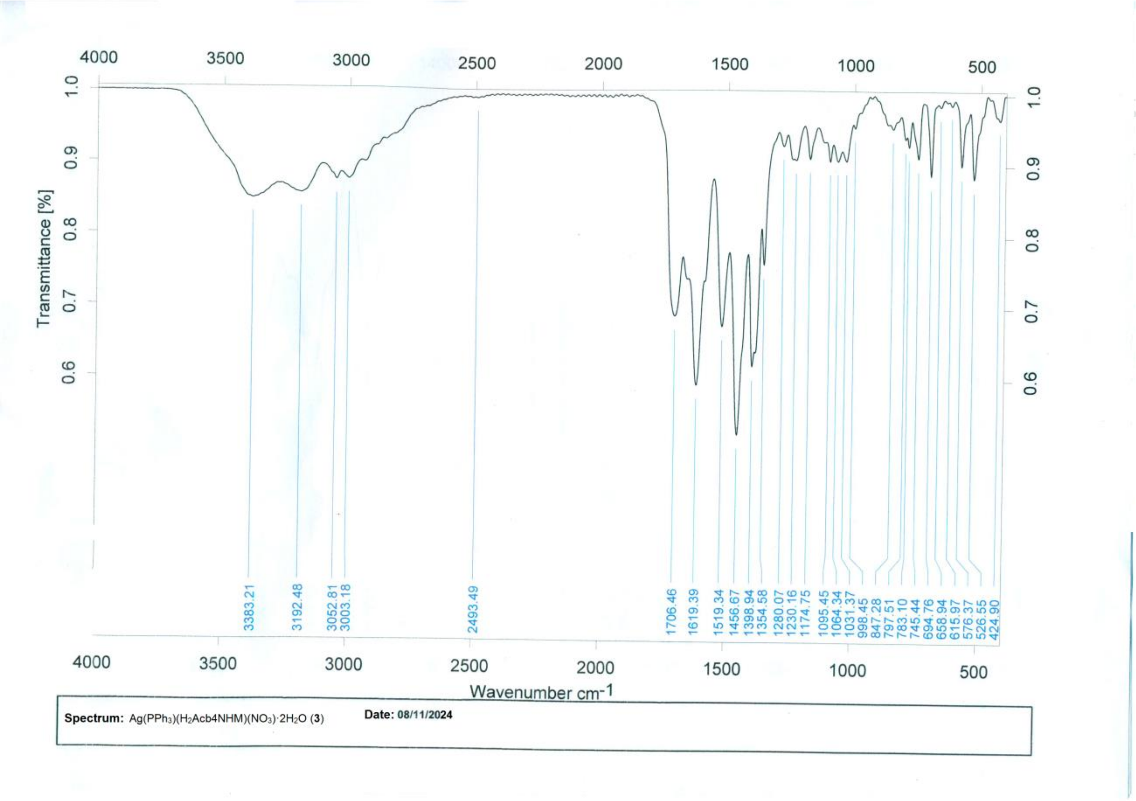

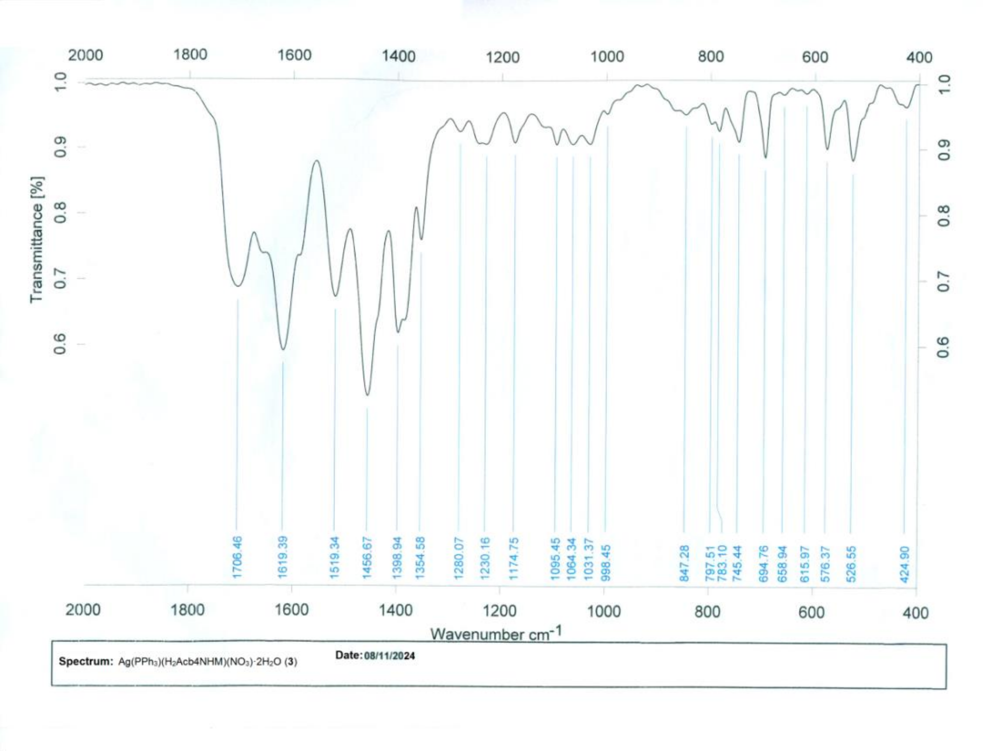

Figures S3.3. FT-IR spectra of **3** (4000-400 cm^{-1})

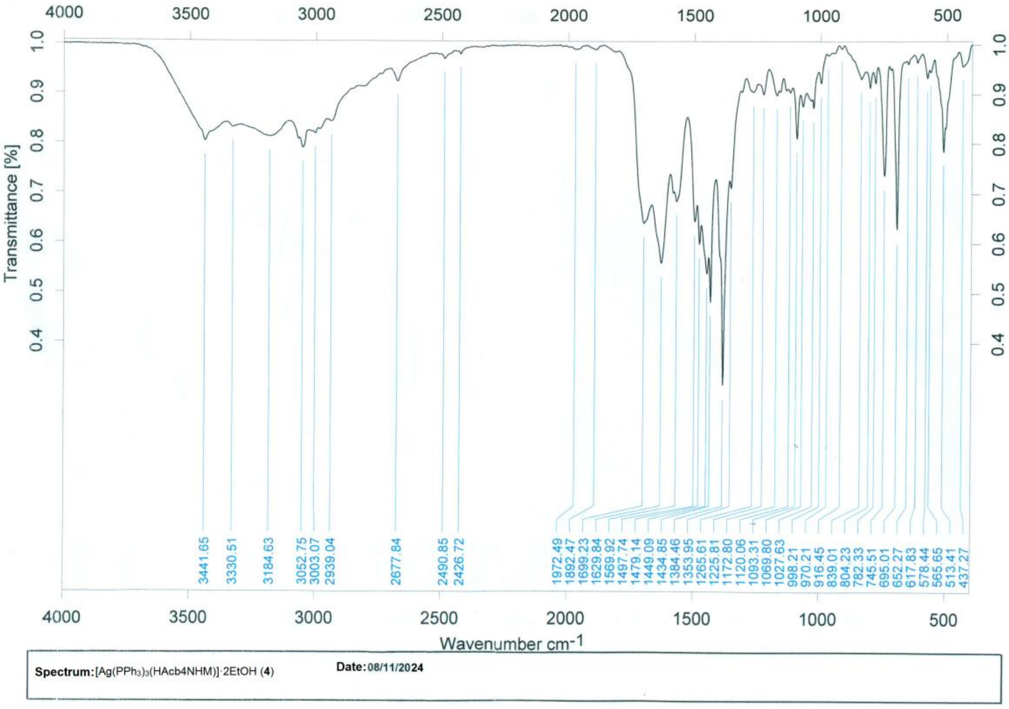

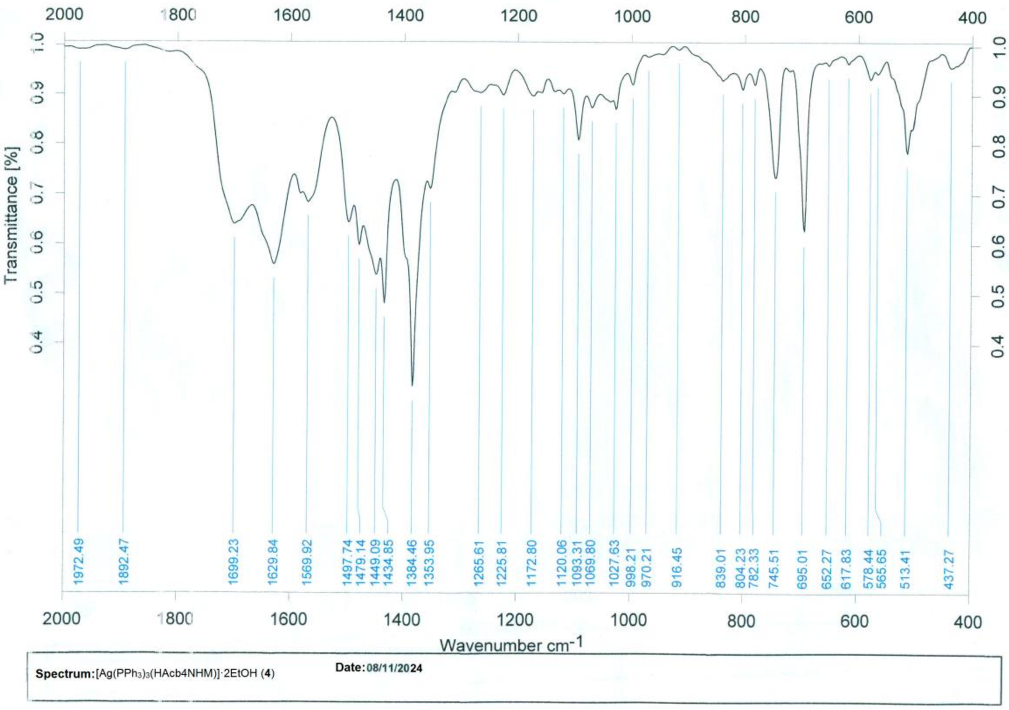

Figures S3.4. FT-IR spectra of **4** (4000-400 cm^{-1})

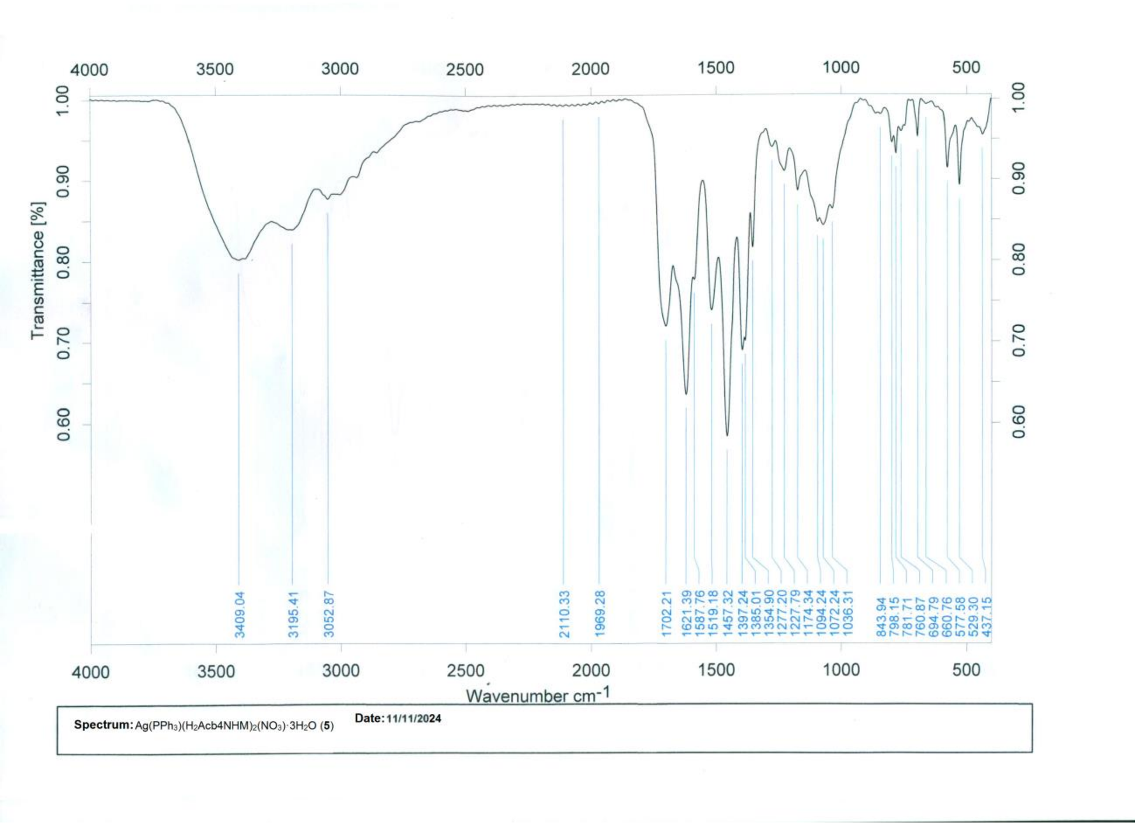

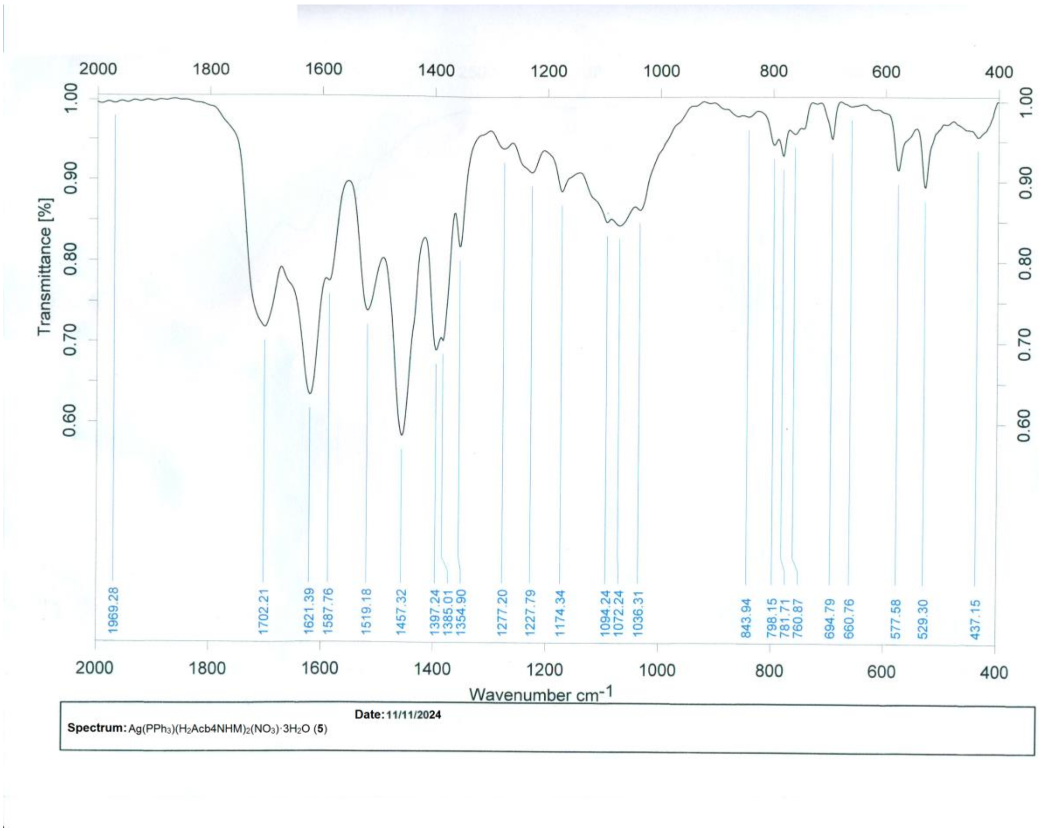

Figures S3.5. FT-IR spectra of **5** (4000-400 cm^{-1})

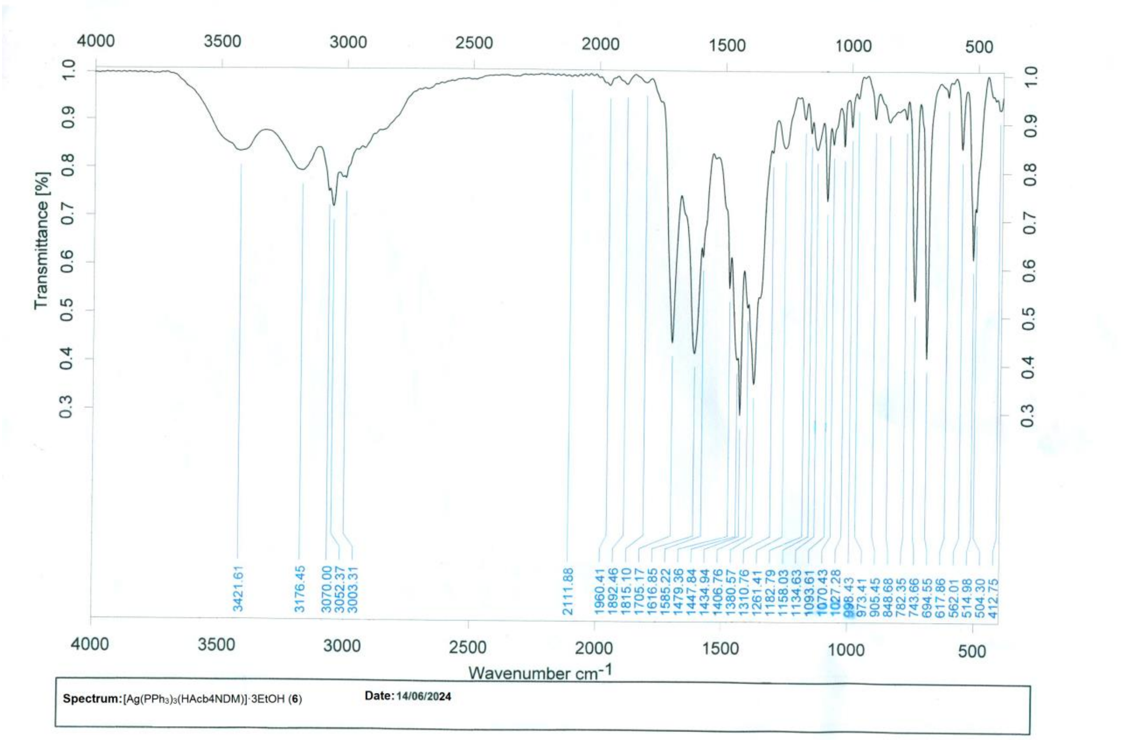

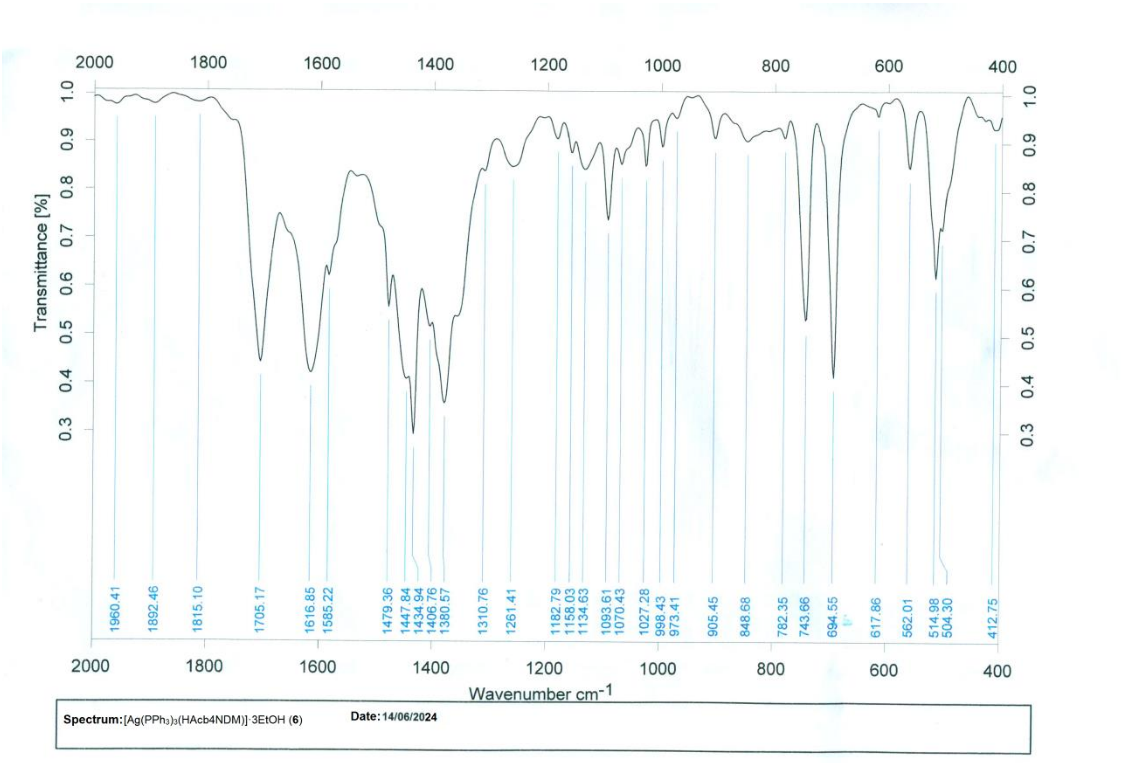

Figures S3.6. FT-IR spectra of **6** (4000-400 cm^{-1})

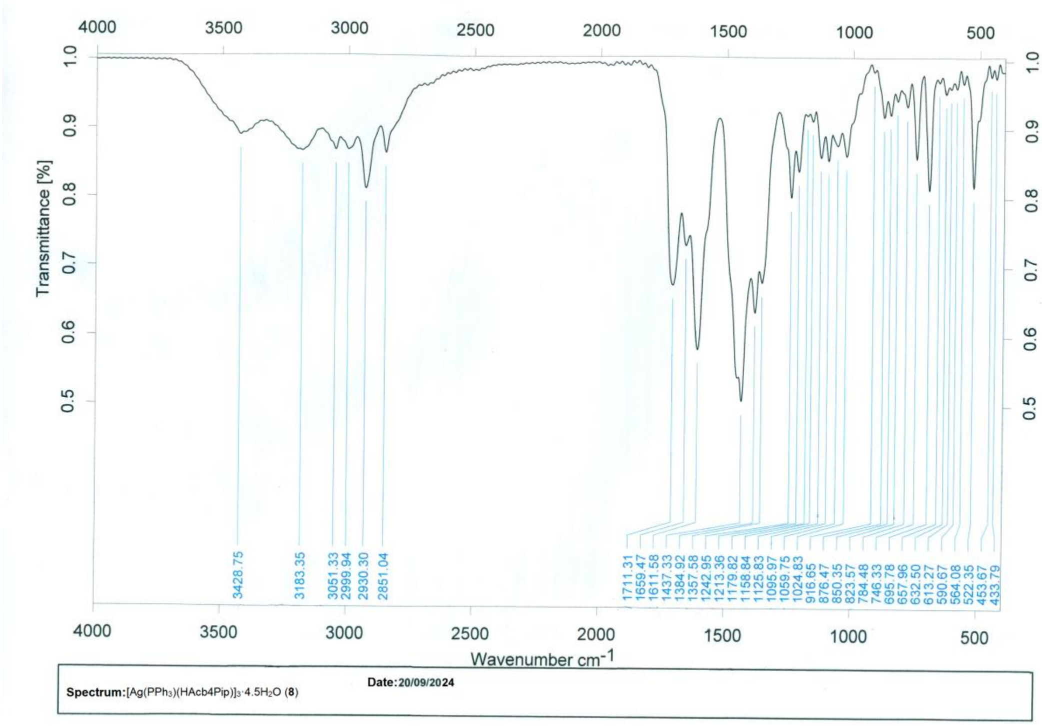

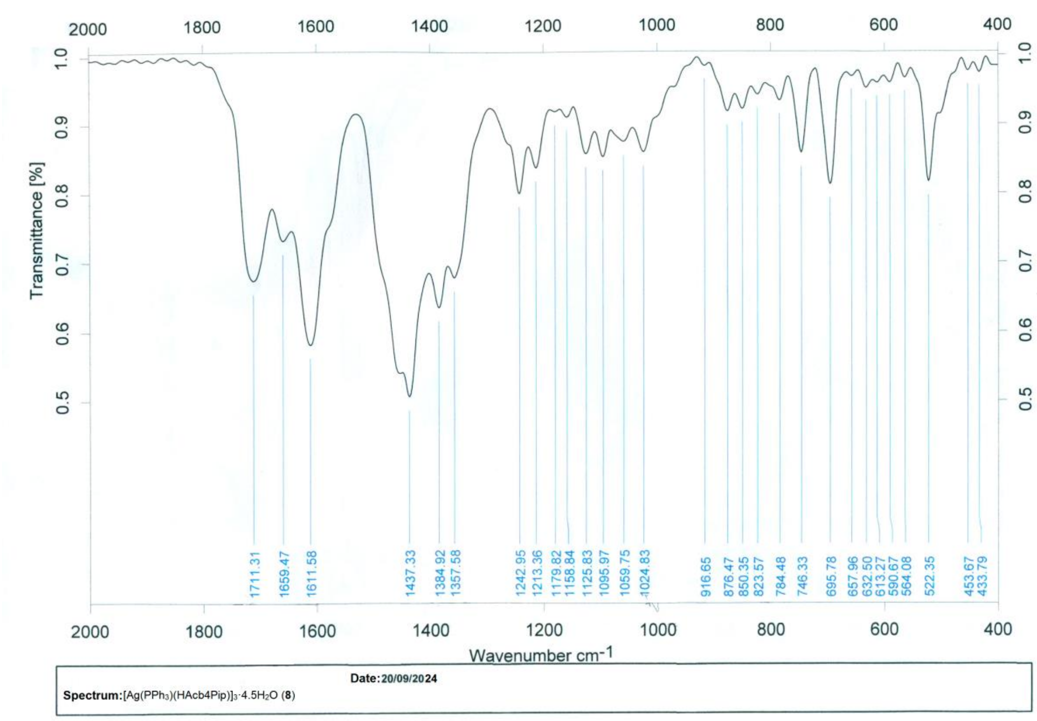

Figures S3.8. FT-IR spectra of **8** (4000-400 cm^{-1})

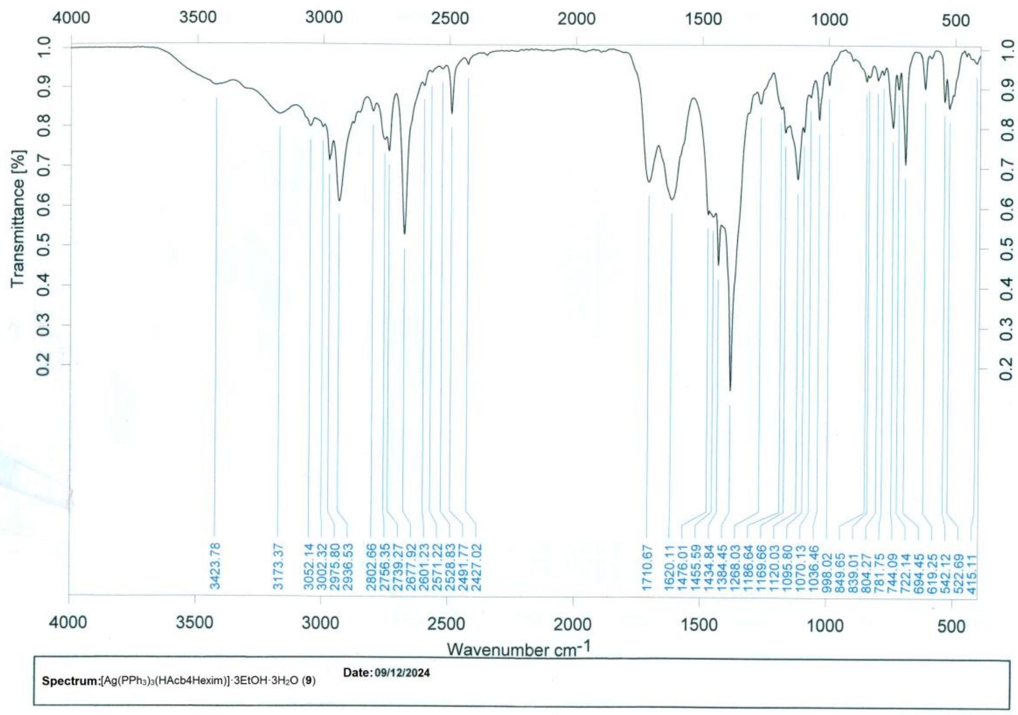

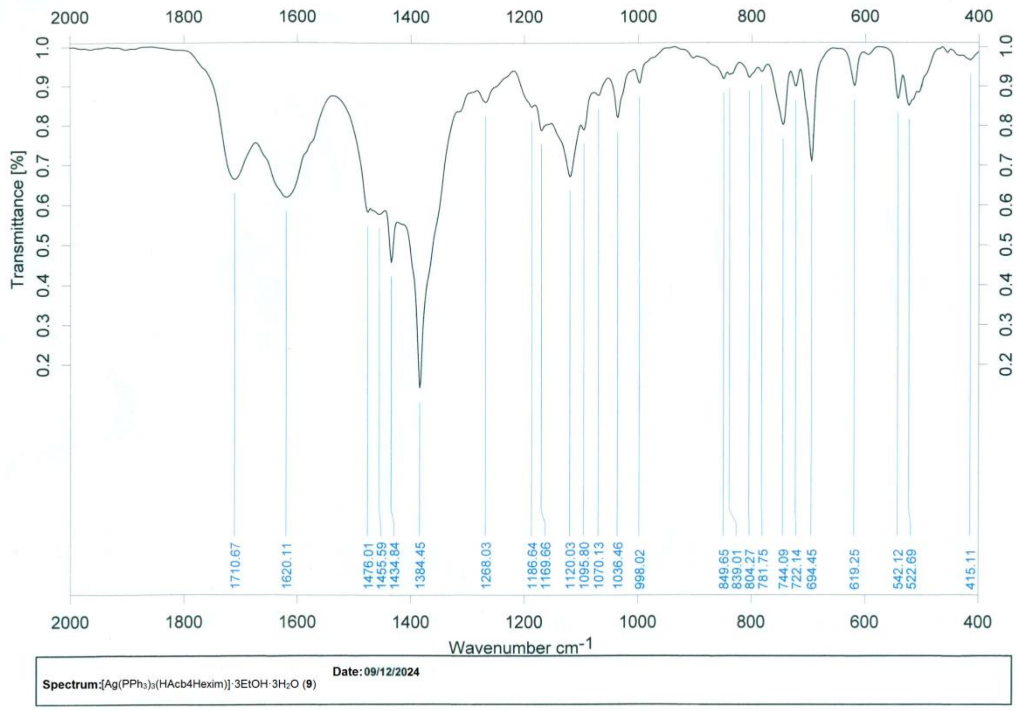

Figures S3.9. FT-IR spectra of **9** (4000-400 cm^{-1})

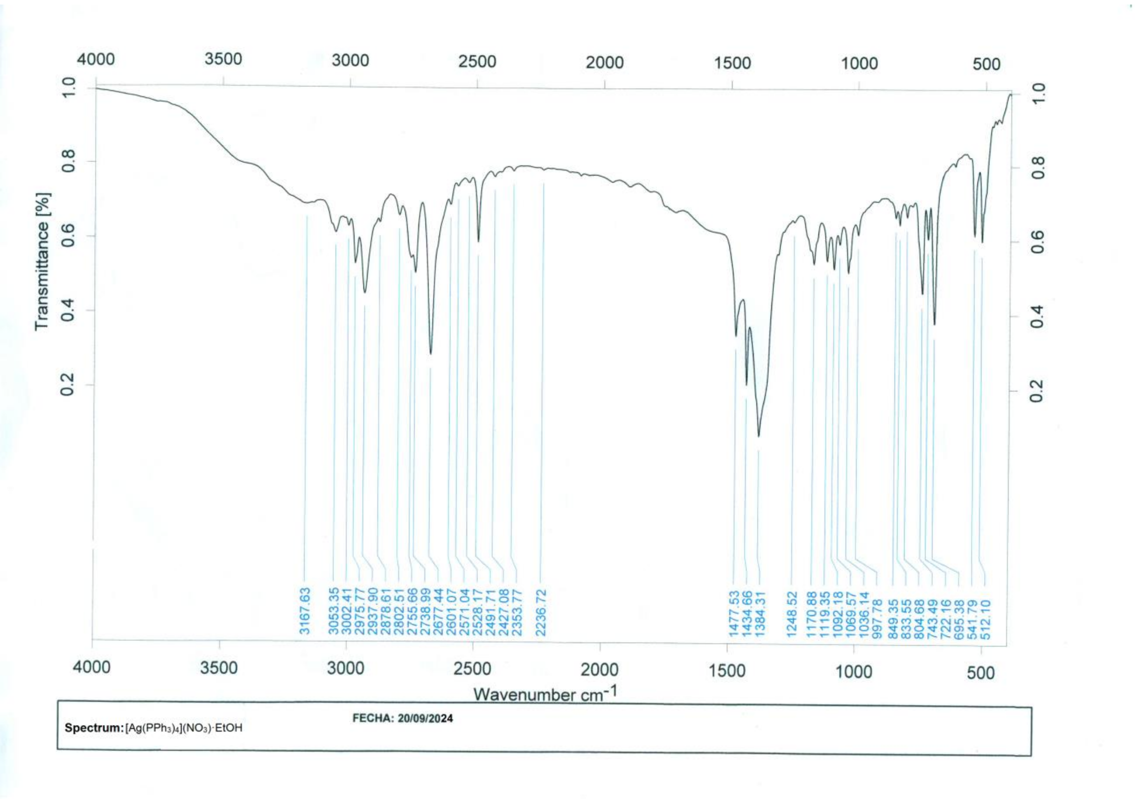

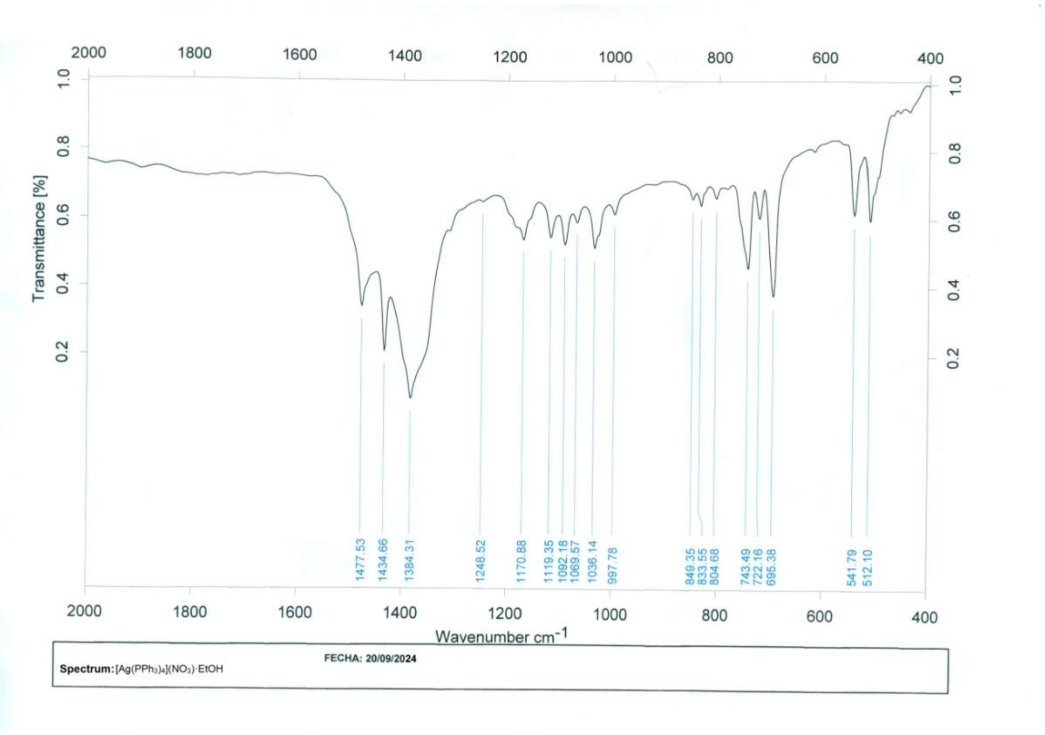

Figures S4. FT-IR spectra of **$[Ag(PPh_3)_4](NO_3)\cdot EtOH$** (4000-400 cm^{-1})

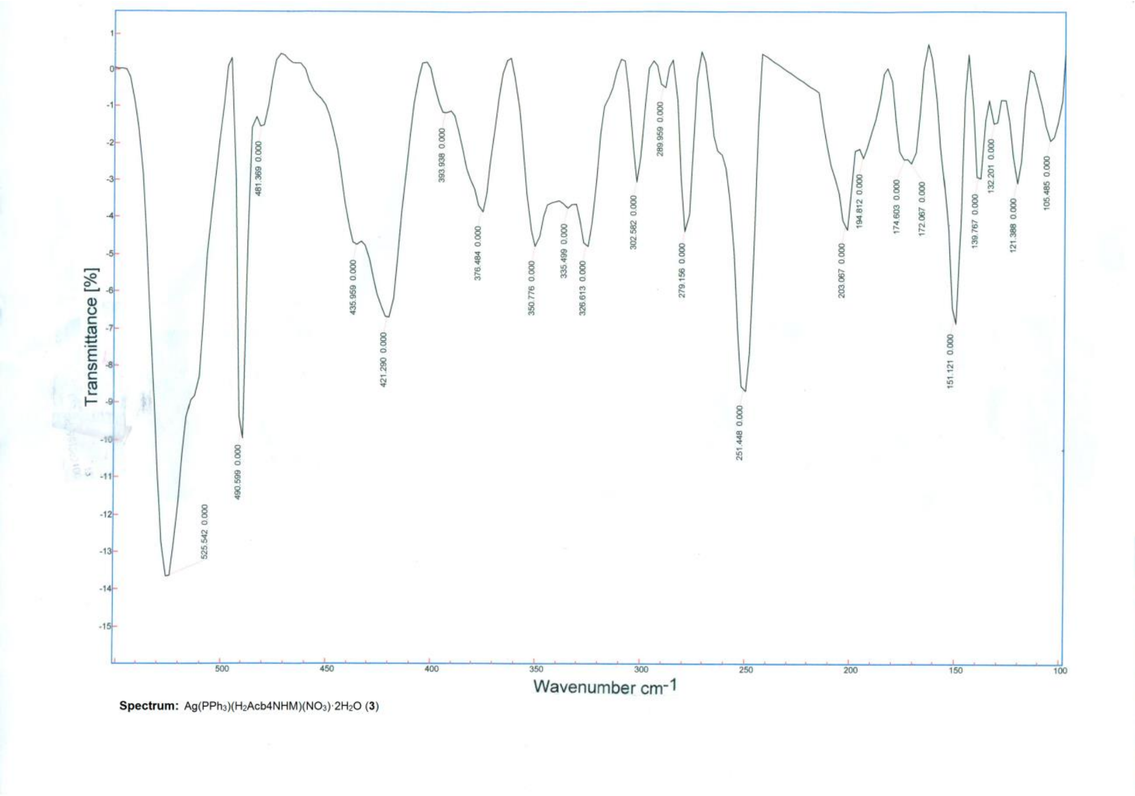

Figure S5.3. FT-IR spectrum of **3** (500-100 cm^{-1})

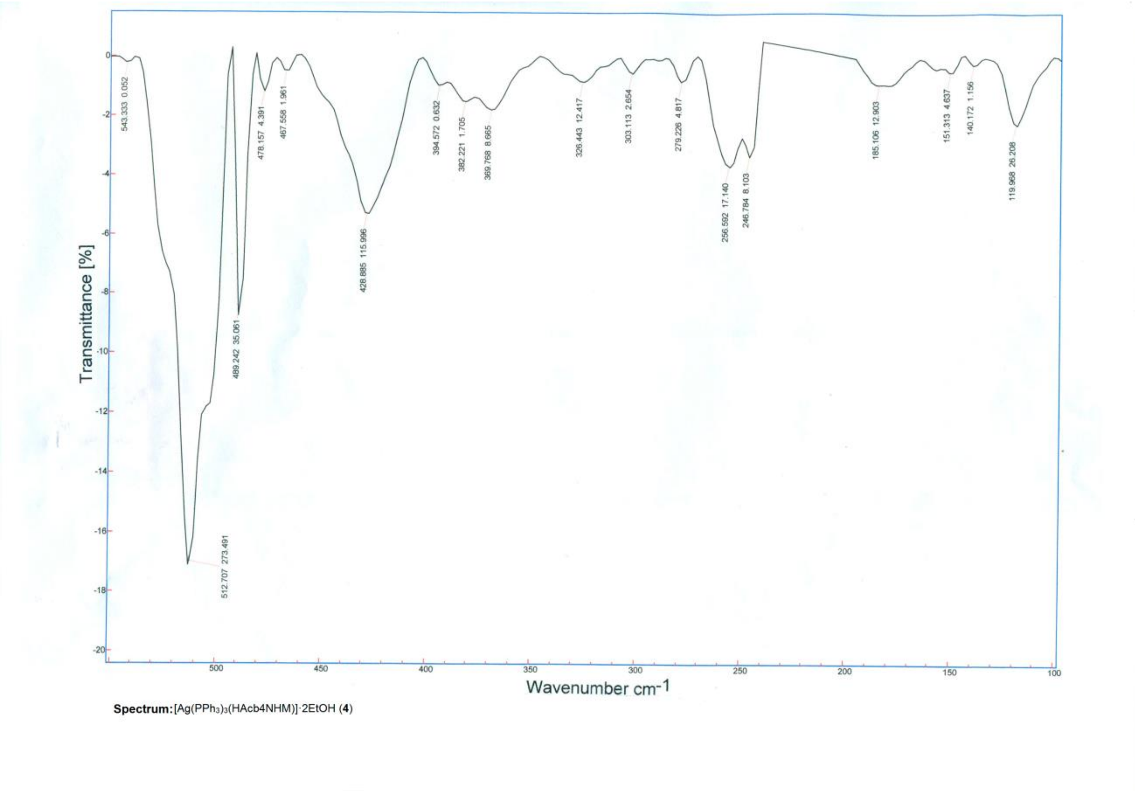

Figure S5.4. FT-IR spectrum of **4** (500-100 cm^{-1})

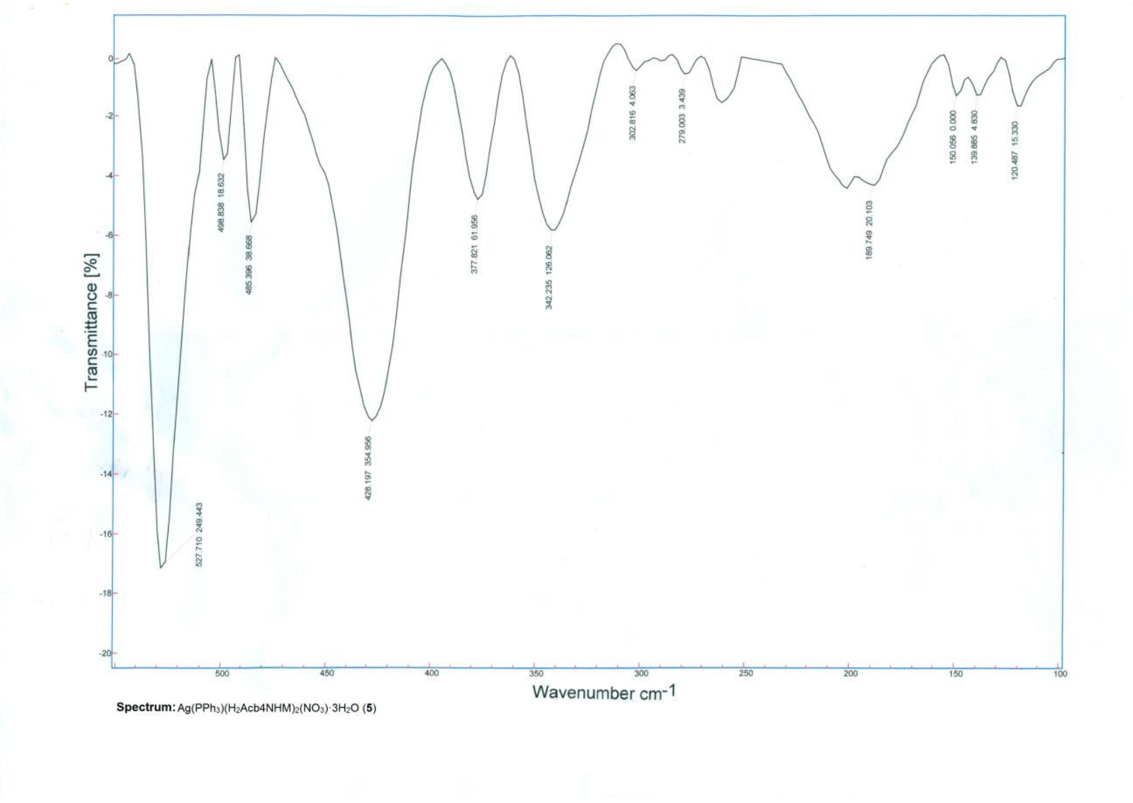

Figure S5.5. FT-IR spectrum of **5** (500-100 cm^{-1})

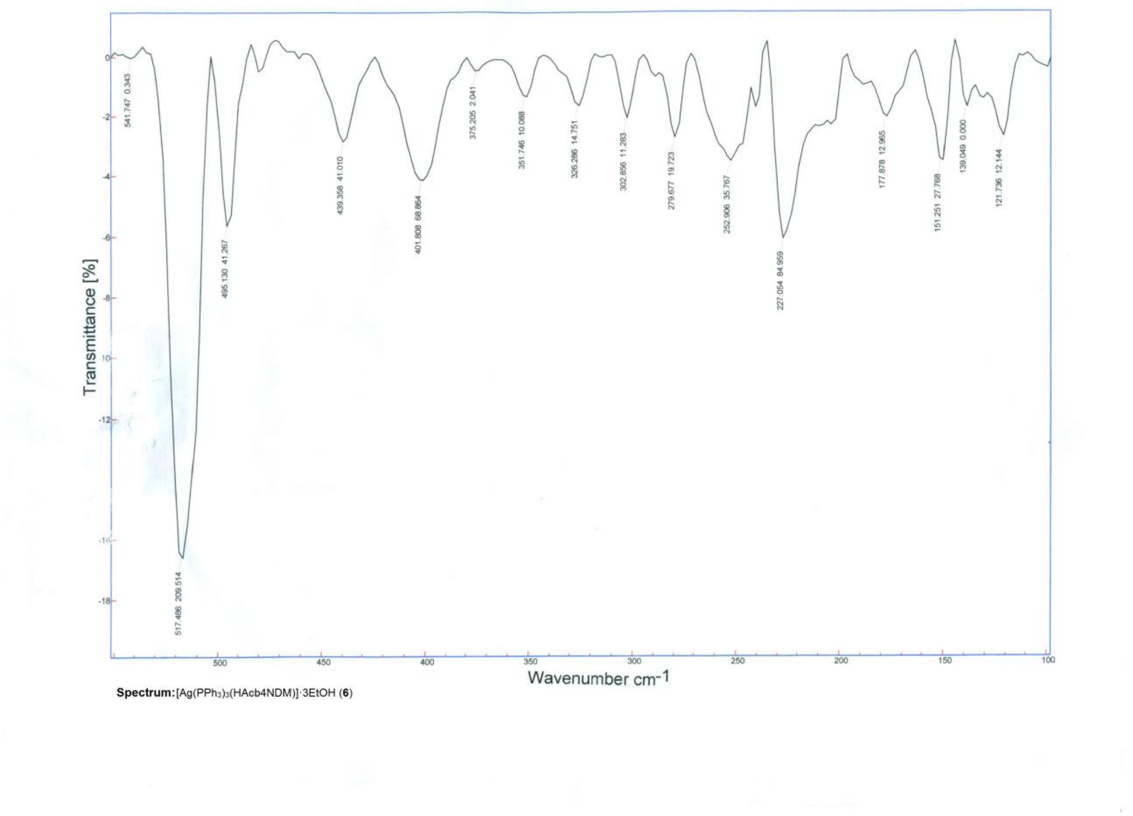

Figure S5.6. FT-IR spectrum of **6** (500-100 cm^{-1})

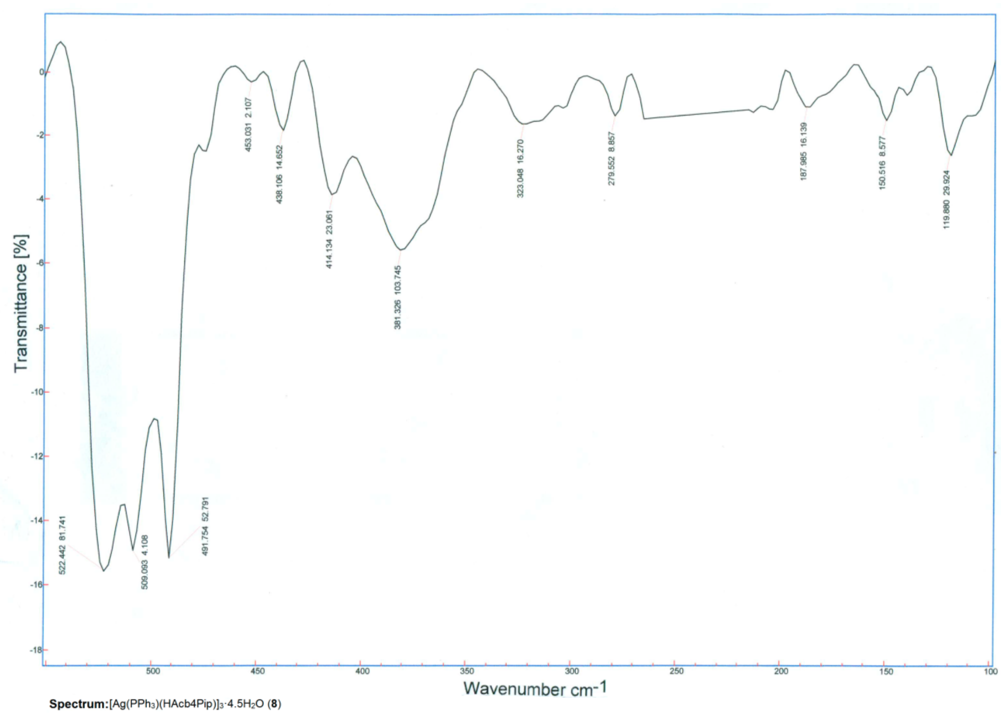

Figure S5.8. FT-IR spectrum of **8** (500-100 cm^{-1})

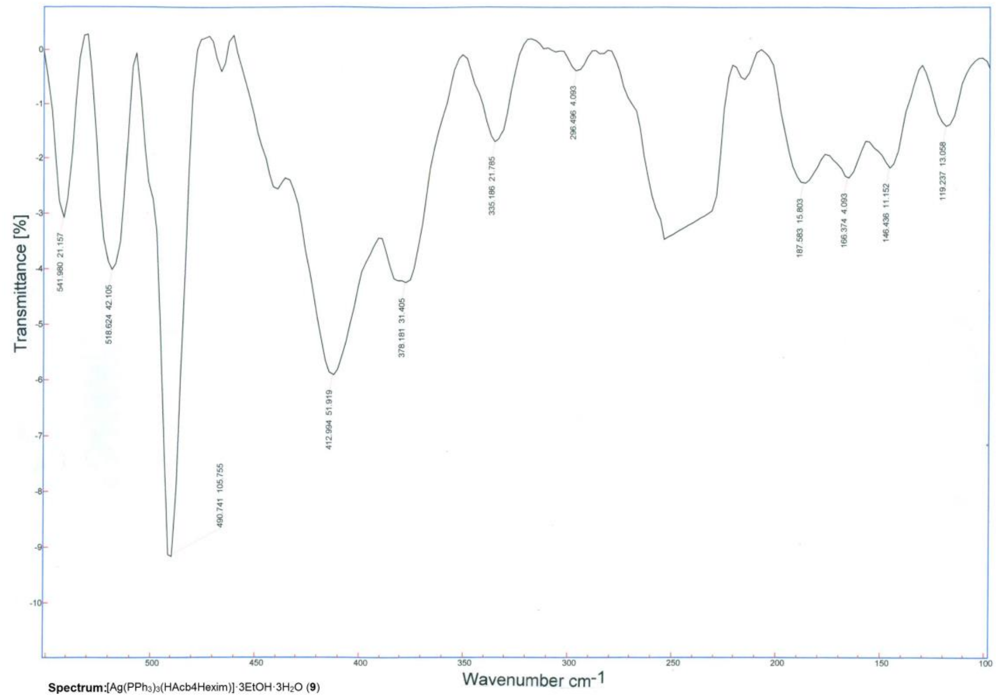

Figure S5.9. FT-IR spectrum of **9** (500-100 cm^{-1})

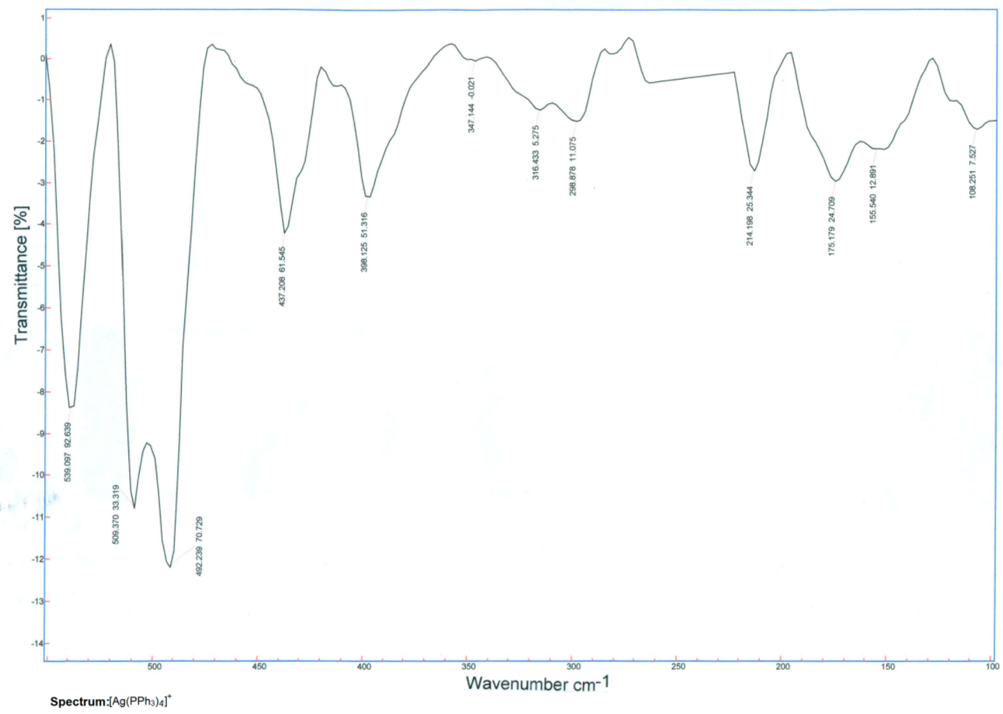

Figure S6. FT-IR spectrum of **[Ag(PPh$_3$)$_4$](NO$_3$)·EtOH** (500-100 cm^{-1})

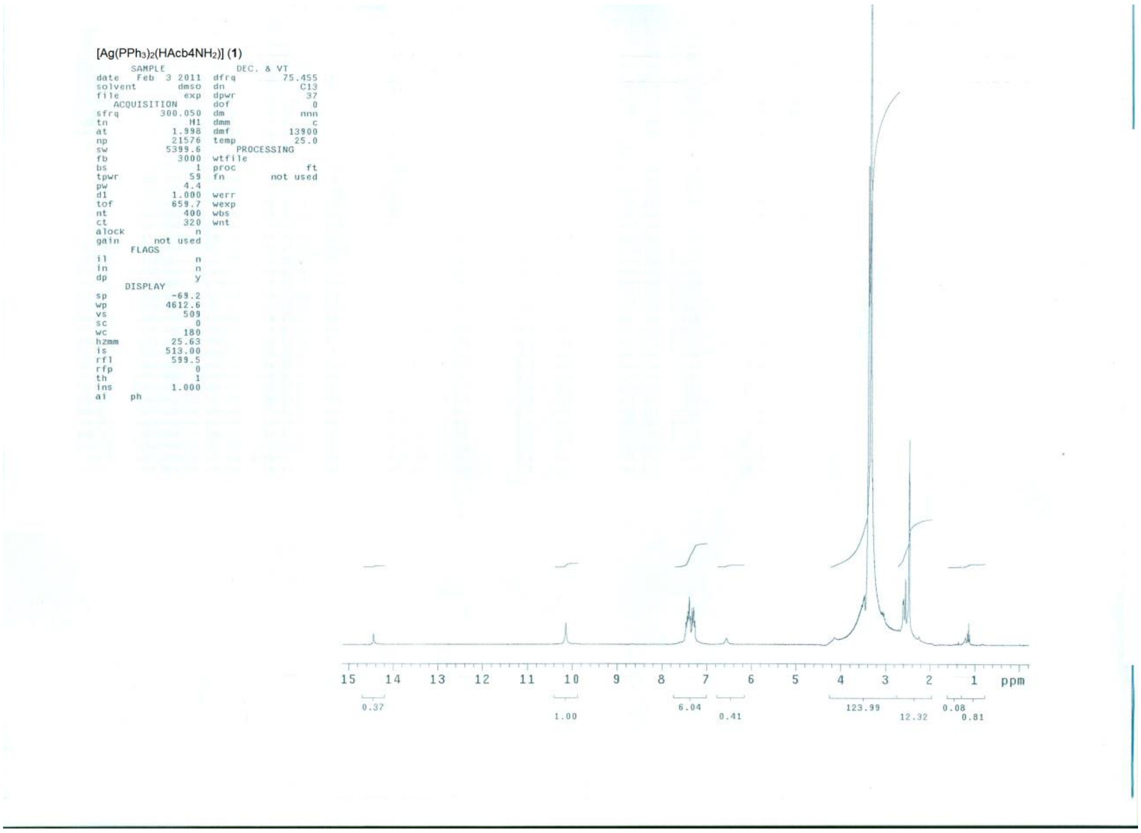

Figure S7.1. ^{1}H NMR spectrum of **1**.

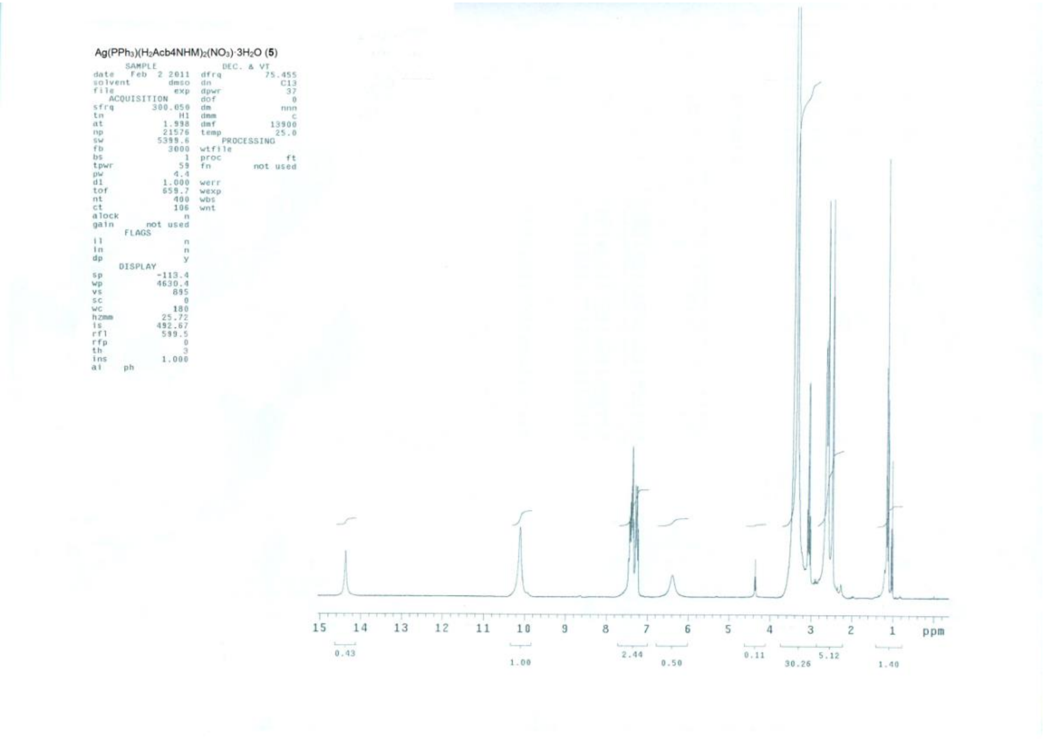

Figure S7.5. ^{1}H NMR spectrum of **5**.

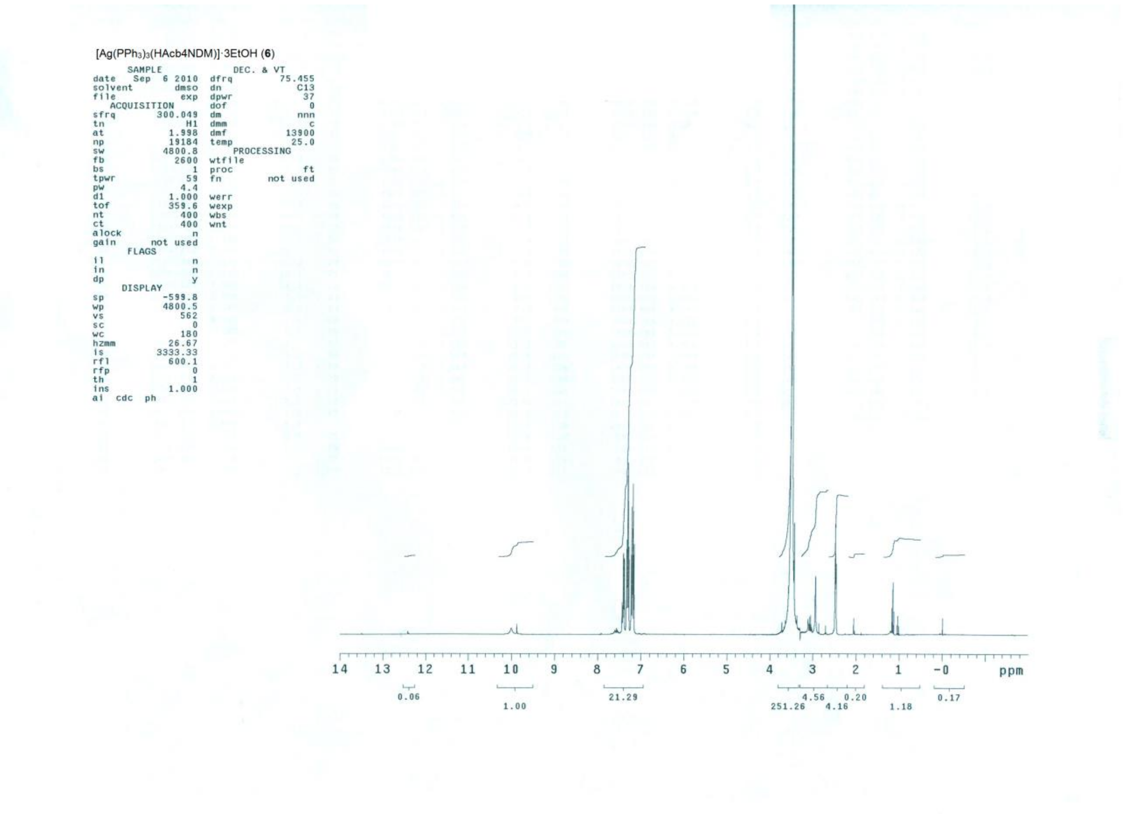

Figure S7.6. ^{1}H NMR spectrum of **6**.

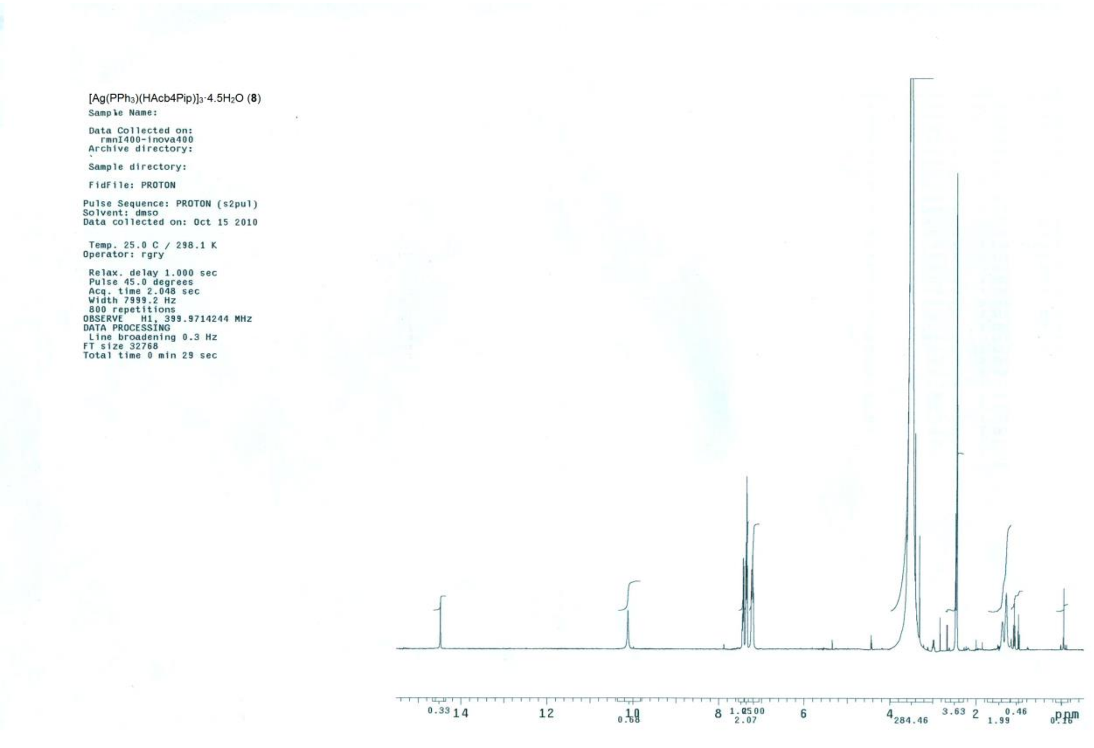

Figure S7.8. ^{1}H NMR spectrum of **8**.

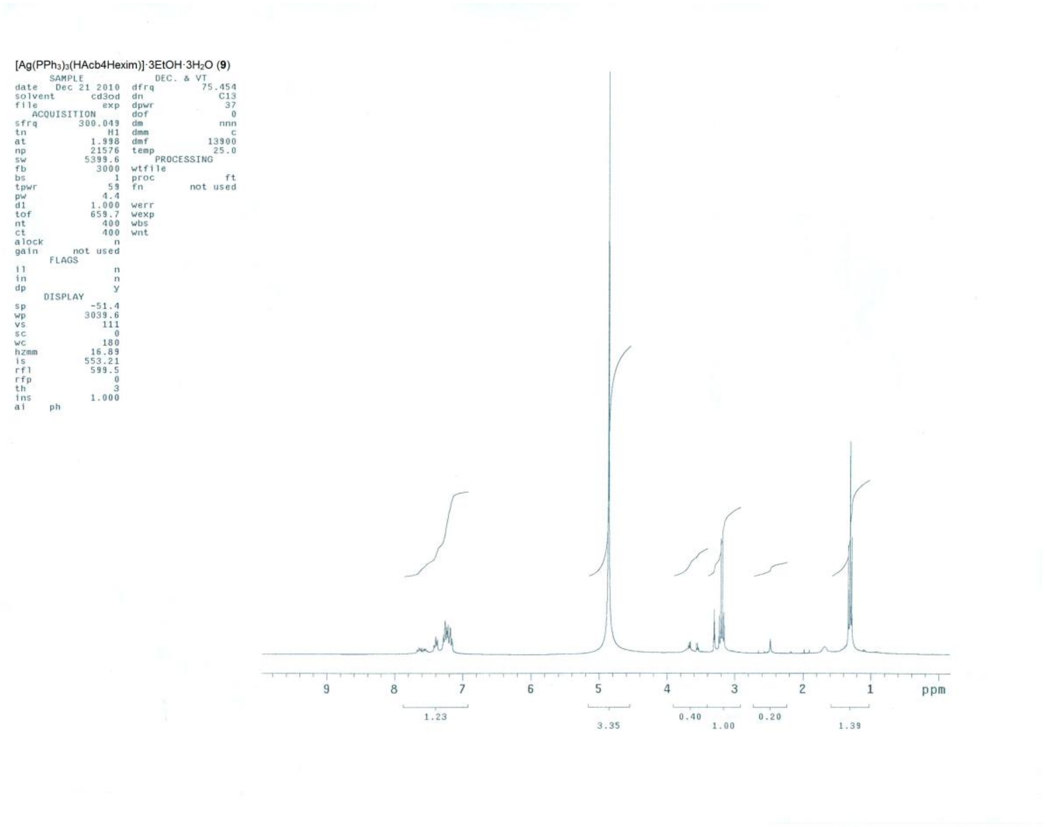

Figure S7.9. ^{1}H NMR spectrum of **9.**

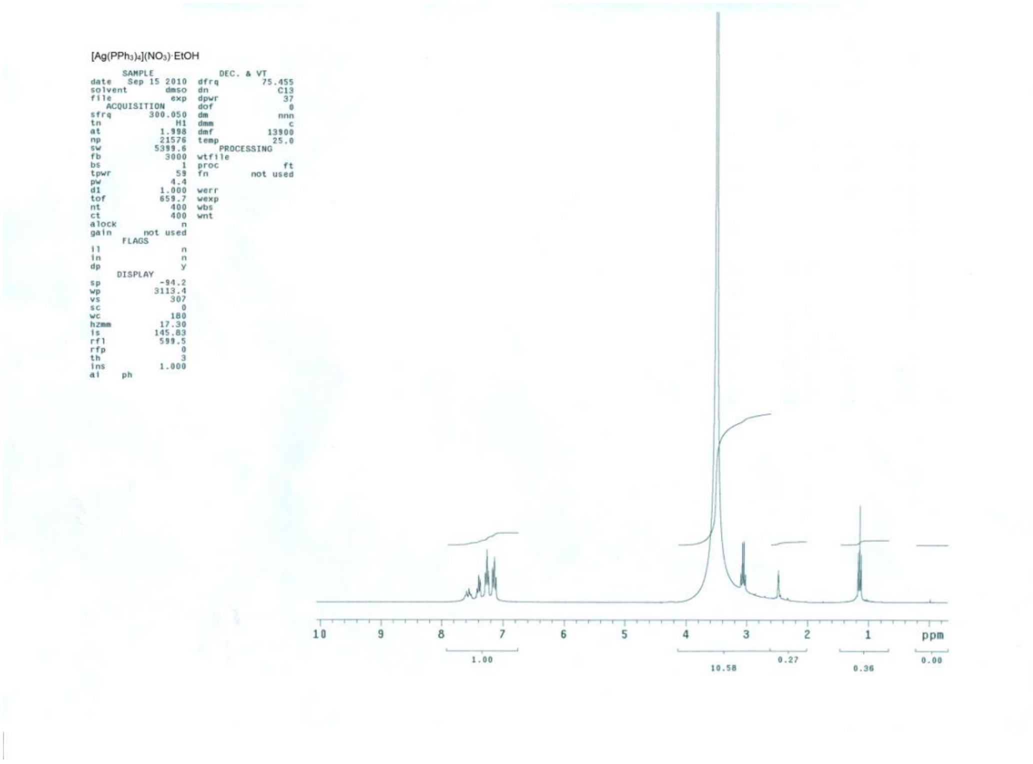

Figure S8. ^{1}H NMR spectrum of **$[Ag(PPh_3)_4](NO_3)·EtOH$**

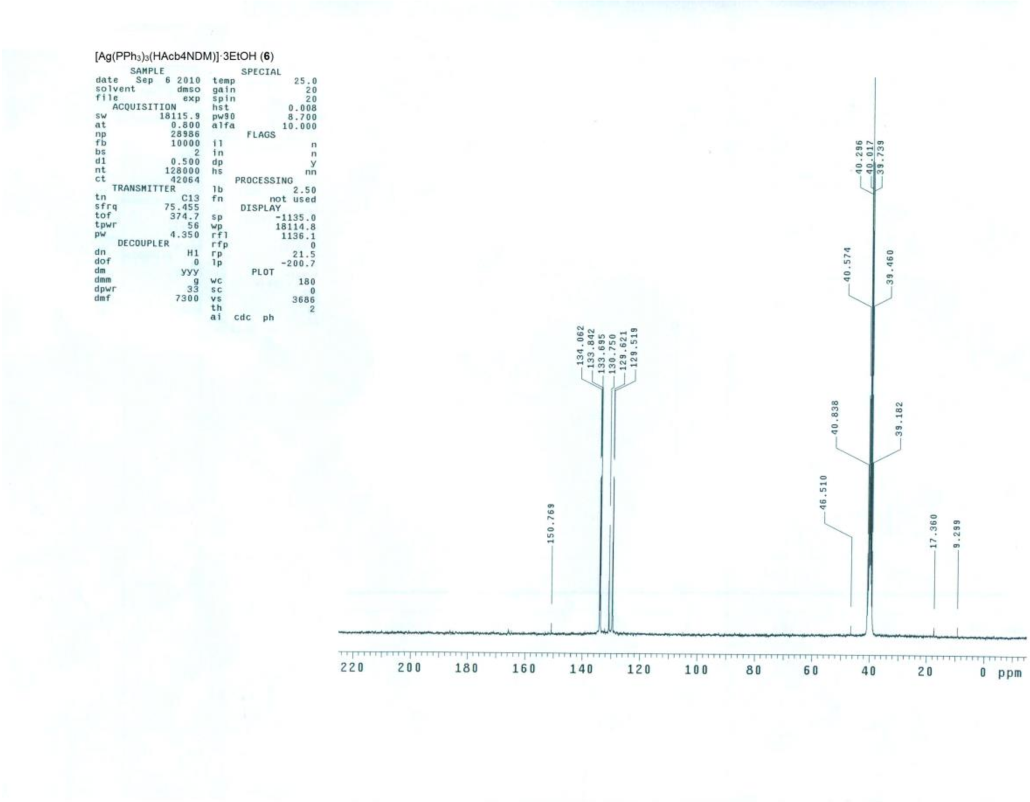

Figure S9.6. ^{13}C NMR spectrum of **6**.

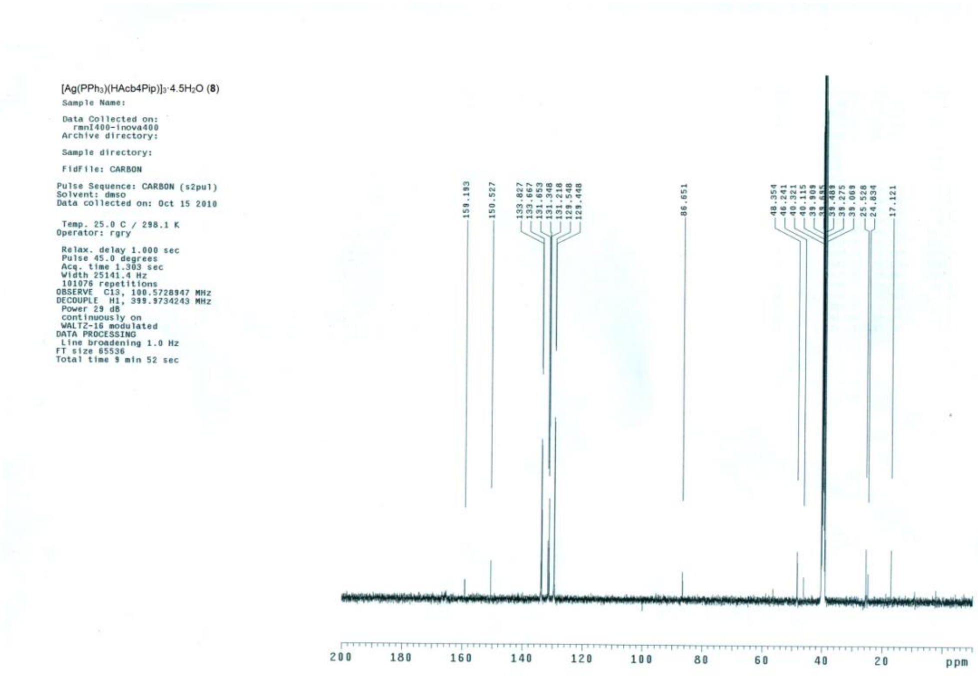

Figure S9.8. ^{13}C NMR spectrum of **8**.

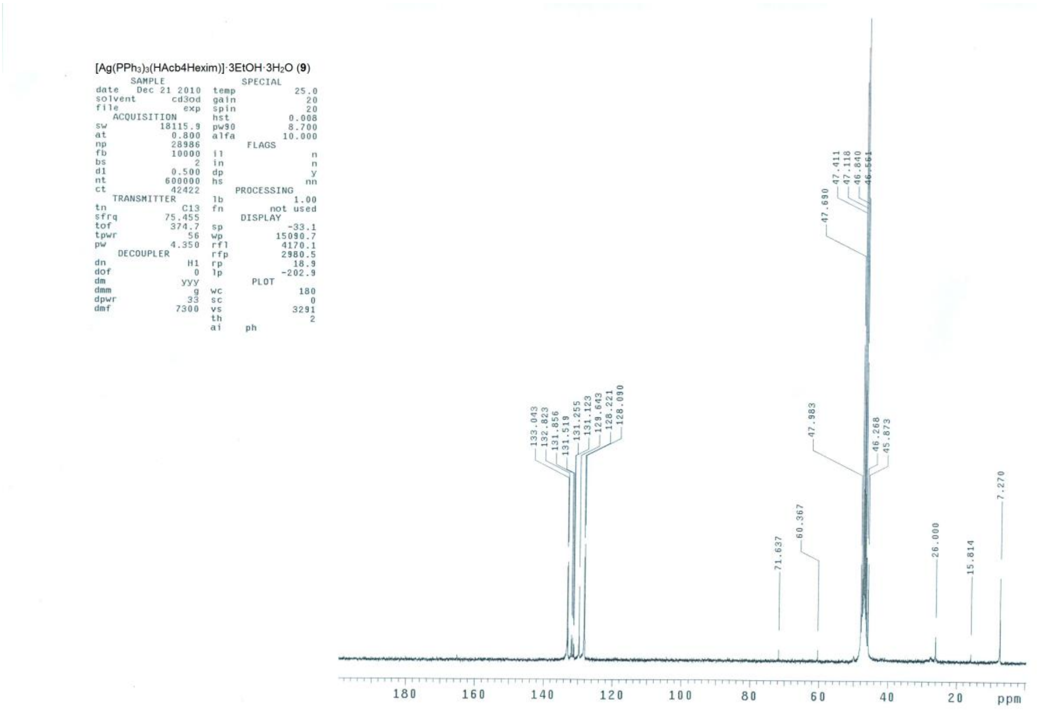

Figure S9.9. ^{13}C NMR spectrum of **9.**

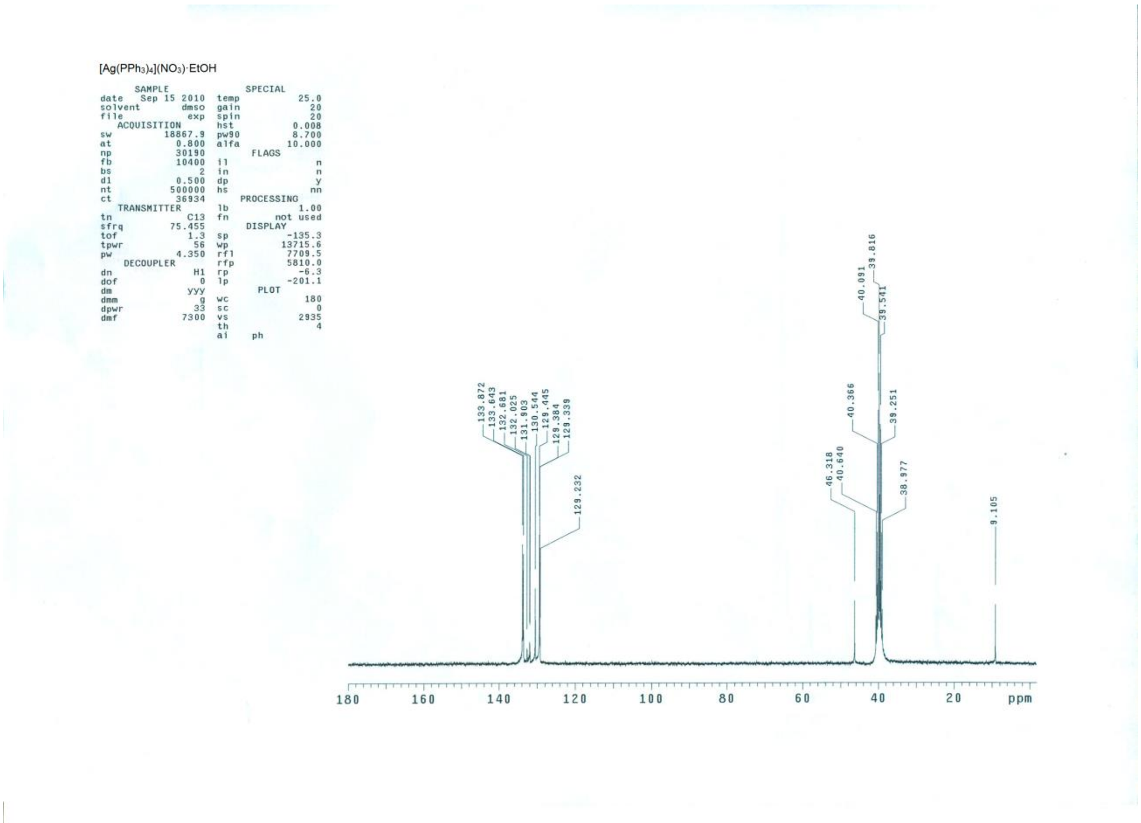

Figure S10. ^{13}C NMR spectrum of **$[Ag(PPh_3)_4](NO_3)\cdot EtOH$**

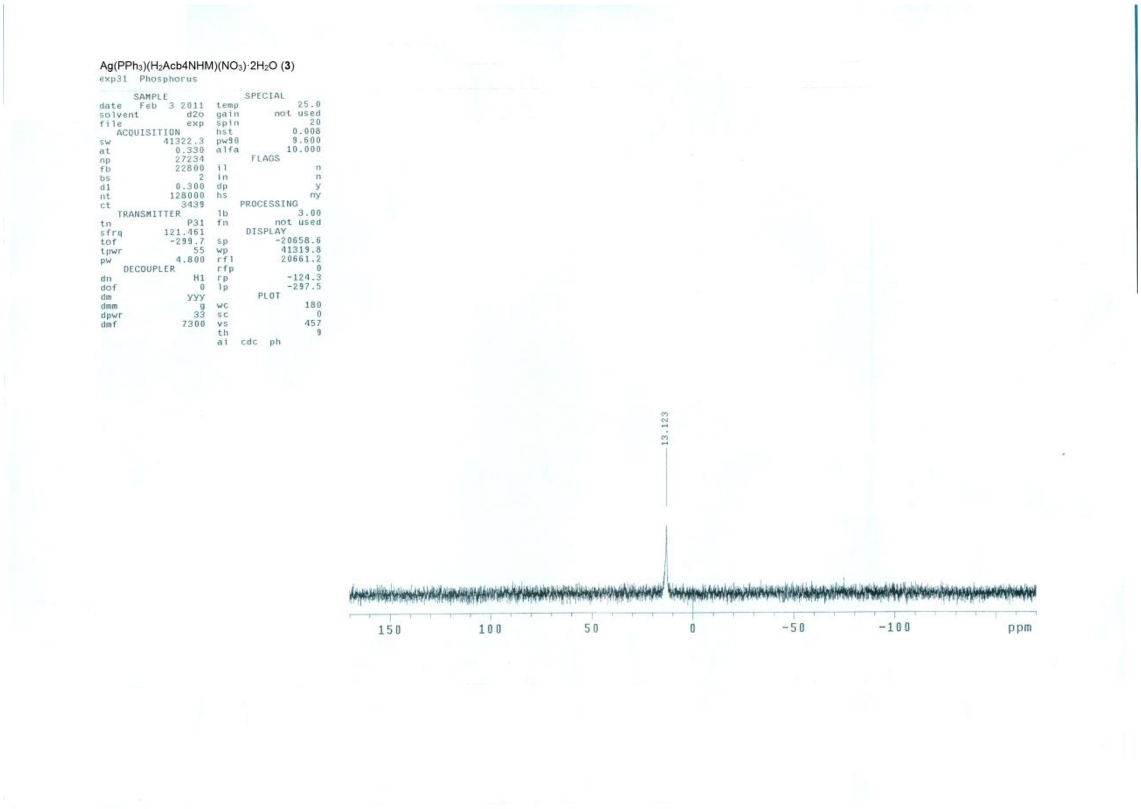

Figure S11.3. ^{31}P NMR spectrum of **3**.

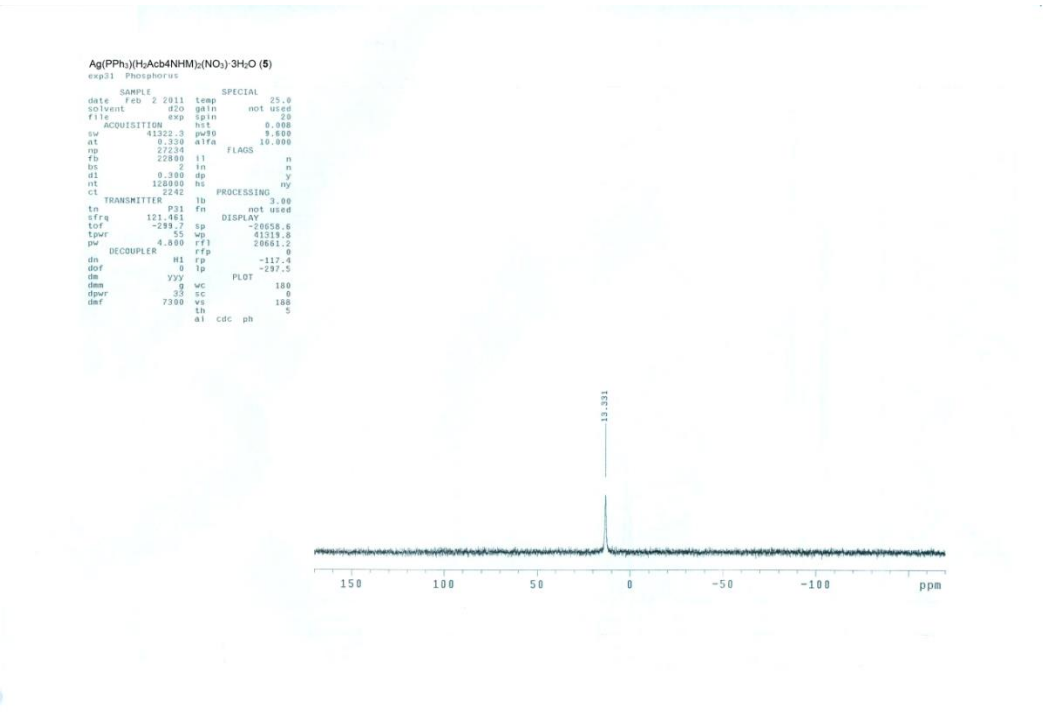

Figure S11.5. ^{31}P NMR spectrum of **5**.

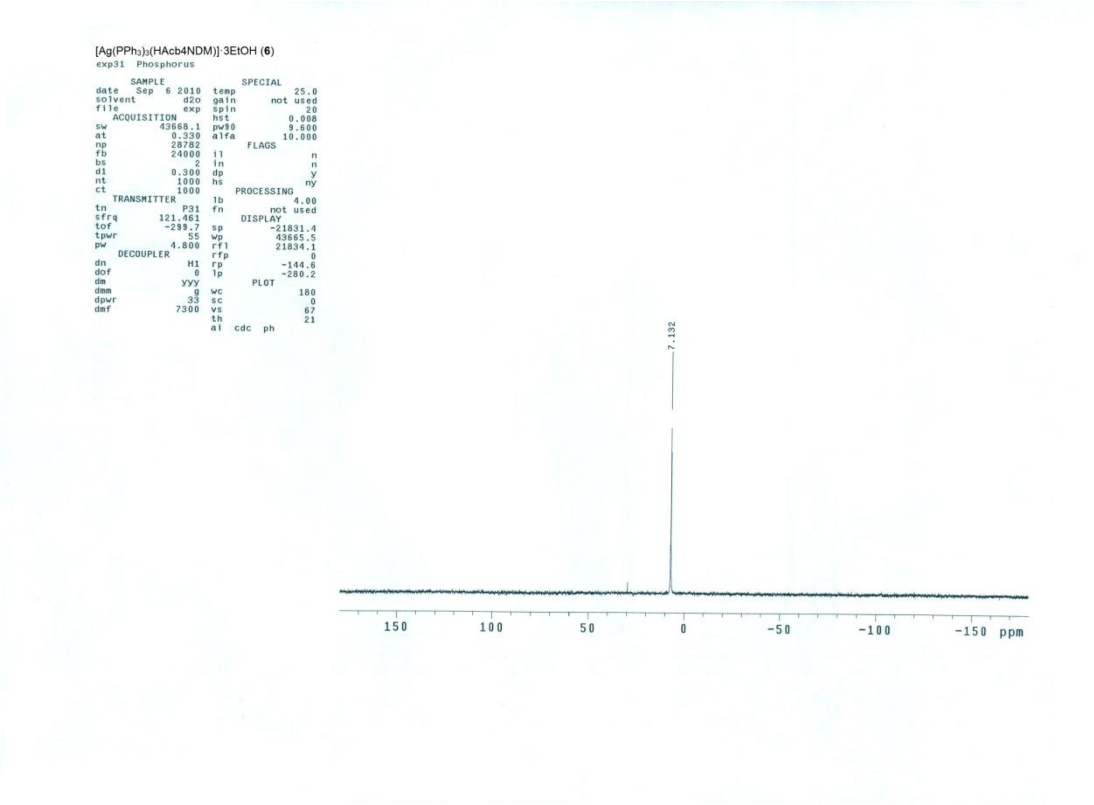

Figure S11.6. ^{31}P NMR spectrum of **6**.

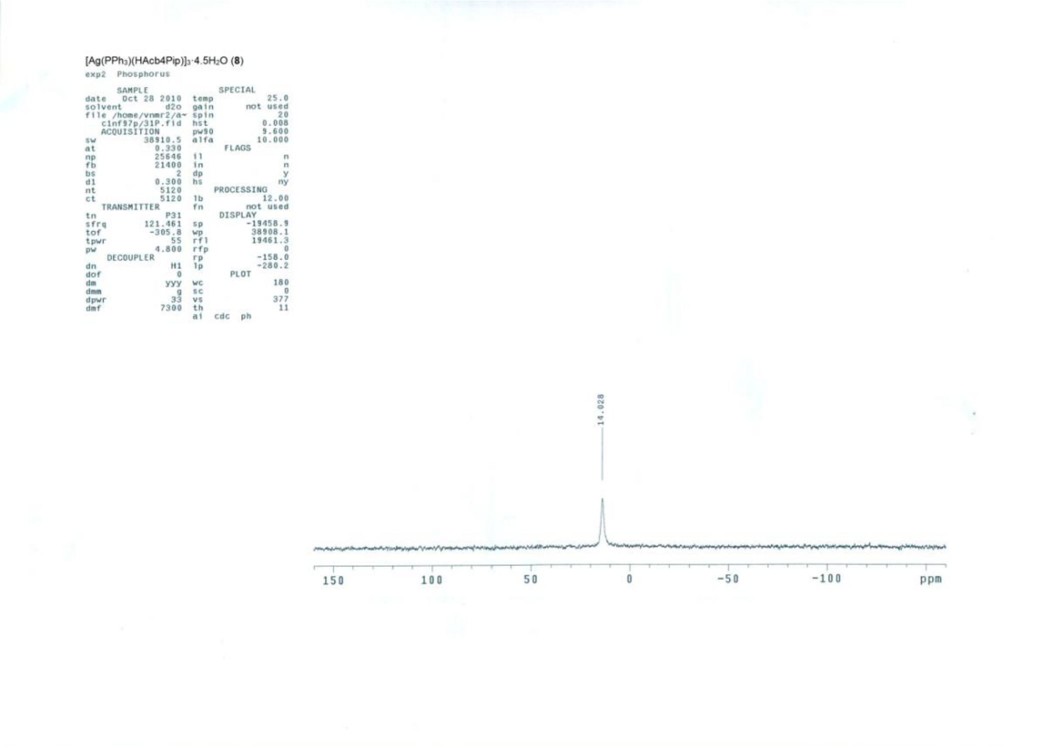

Figure S11.8. ^{31}P NMR spectrum of **8**.

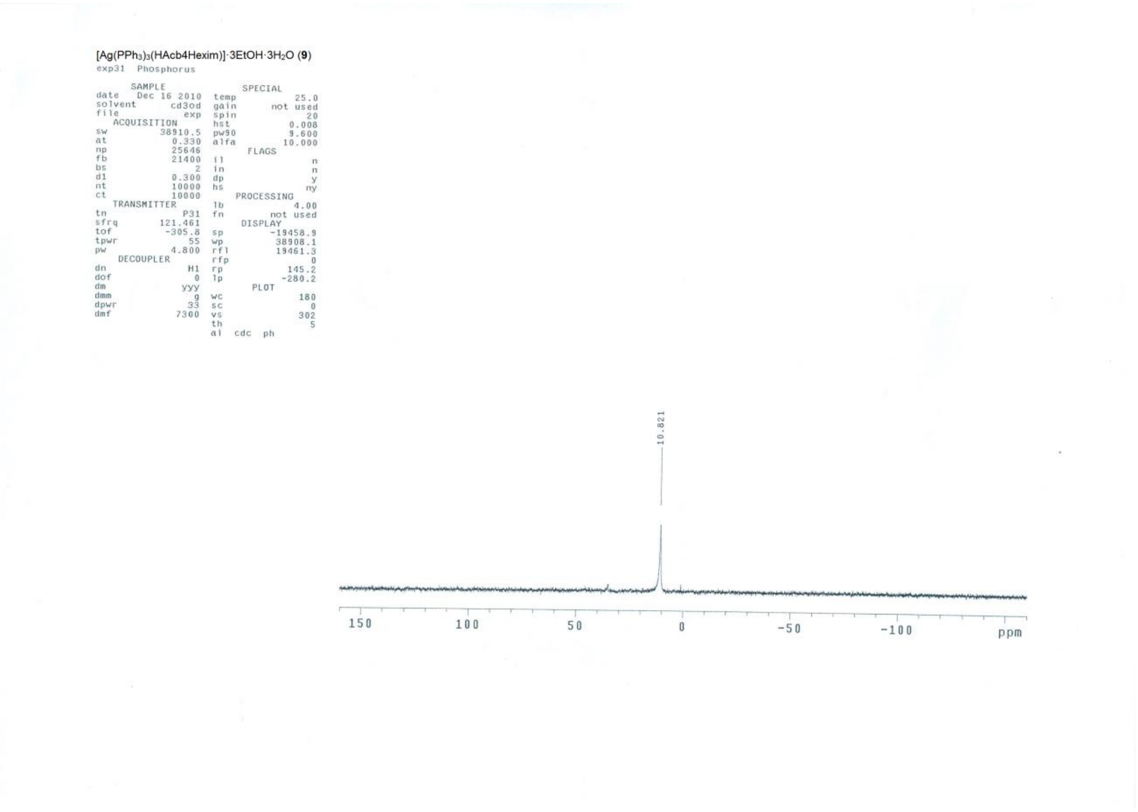

Figure S11.9. ^{31}P NMR spectrum of **9**

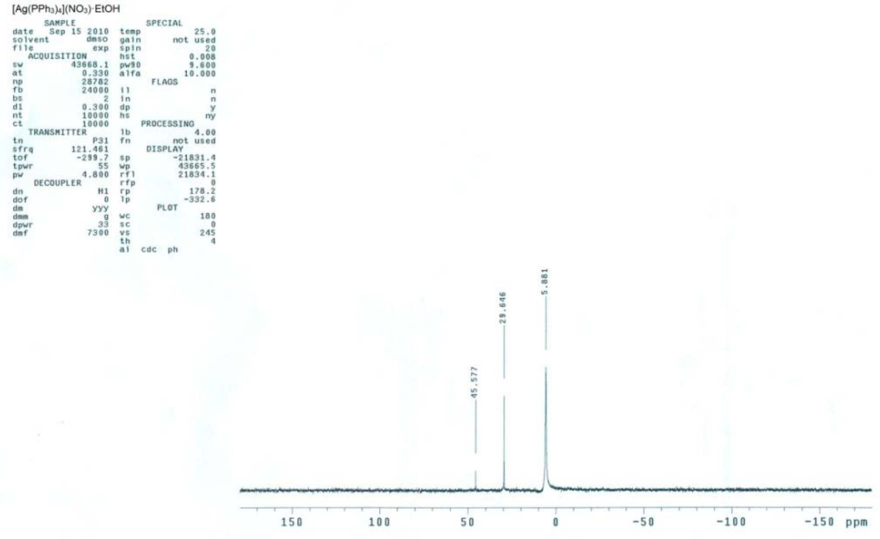

Figure S12. ^{31}P NMR spectrum of $[Ag(PPh_3)_4](NO_3)\cdot EtOH$ (25°C)

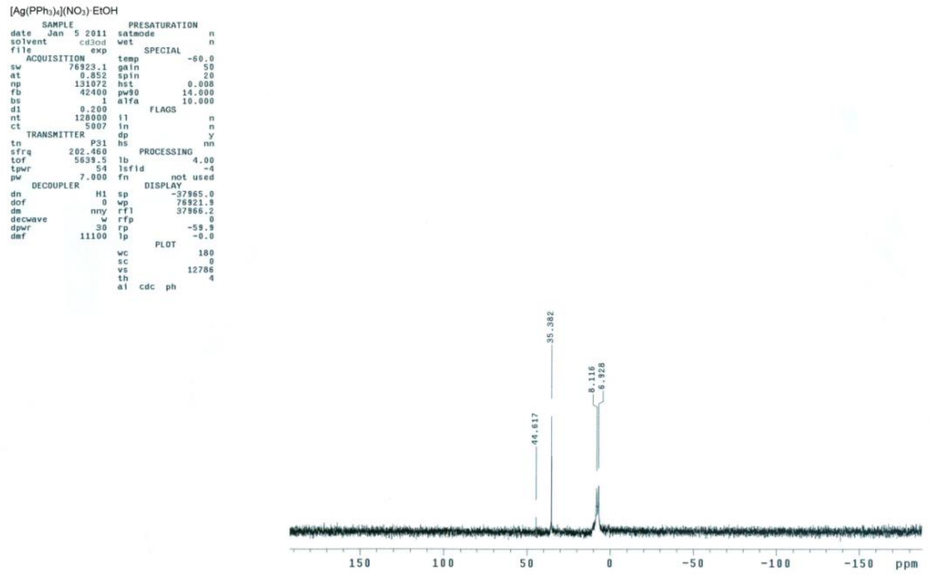

Figure S13. ^{31}P NMR spectrum of $[Ag(PPh_3)_4](NO_3)\cdot EtOH$ (-60°C)
